# Site‐Specific Multi‐Functionalization of the Carrier Protein CRM_197_ by Disulfide Rebridging for Conjugate Vaccine Development

**DOI:** 10.1002/cbic.202200408

**Published:** 2022-09-29

**Authors:** Nino Trattnig, Zeshi Li, Gerlof P. Bosman, Paul Kosma, Geert‐Jan Boons

**Affiliations:** ^1^ Department of Chemical Biology and Drug Discovery Utrecht Institute for Pharmaceutical Sciences Utrecht University 3584 CG Utrecht The Netherlands; ^2^ Department of Chemistry University of Natural Resources and Life Sciences Muthgasse 18 A-1190 Vienna Austria; ^3^ Complex Carbohydrate Research Center University of Georgia Athens GA 30602 USA; ^4^ Bijvoet Center for Biomolecular Research Utrecht University 3584 CH Utrecht The Netherlands; ^5^ Chemistry Department University of Georgia Athens GA 30602 USA

**Keywords:** antigens, bioconjugation, carbohydrates, protein modification, vaccines

## Abstract

Conjugation of an antigen to a carrier protein is widely used for vaccine development. To develop the next generation of conjugate vaccines, we describe here a method for the controlled multi‐functionalization of the widely employed carrier protein CRM_197_ with a carbohydrate‐based antigen and an immune potentiator. The approach is based on the selective reduction of one of the disulfides of CRM_197_ followed by disulfide rebridging employing an appropriately functionalized dibromopyridazinedione. Efficient protein modification required that the reduction and functionalization with a dibromopyridazinedione was performed as a one‐step procedure with control over the reaction temperature. Furthermore, ligations were most successful when dibromopyridazinediones were employed having a functional entity such as a TLR7/8 agonist and a cyclooctyne for further modification. Site‐specific conjugation avoids modification of T‐epitopes of the carrier protein and covalent attachment of an immune potentiator will ensure that cytokines are produced where the vaccine interacts with relevant immune cells resulting in efficient immune potentiation.

## Graphical abstract

**A method** for the controlled multi‐functionalization of the widely employed carrier protein CRM_197_ with a carbohydrate‐based antigen and an immune potentiator has been developed based on the selective reduction of one of the disulfides of CRM_197_ followed by disulfide rebridging employing an appropriately functionalized dibromopyridazinedione.

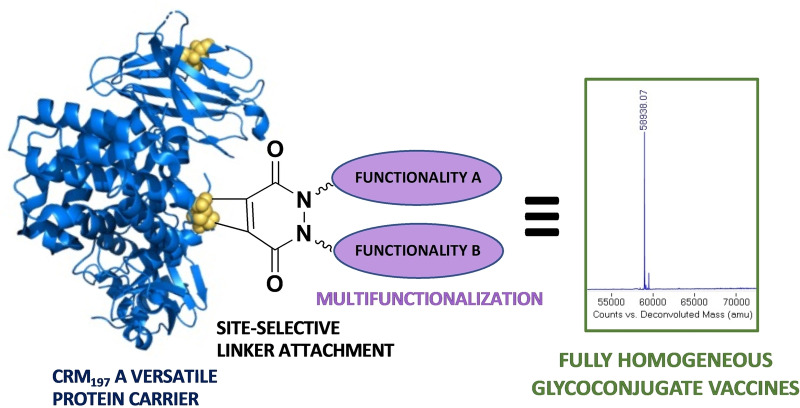

## Introduction

Subunit vaccines, in which only a microbial component is administered, have greatly contributed to vaccine safety.^[1]^ Microbial polysaccharide‐based vaccines offer a particularly important class of subunit vaccines.^[2]^ The first polysaccharide vaccine was introduced in 1983 and composed of capsular polysaccharides isolated from 14 pneumonia serotypes. In healthy adults, this type of vaccine induces appropriate protection. However, in high‐risk groups such as neonates, children under 2 years of age, and the elderly, these vaccines elicit poor antibody responses that do not provide proper protection. Polysaccharide‐based vaccines cannot induce T‐cell responses, and therefore only less effective IgM antibodies are elicited. A revolutionary technology that addresses this limitation is based on the conjugation of bacterial polysaccharides to a foreign carrier protein[^2^, ^3^] that provides helper T‐epitopes thereby overcoming T‐cell independent immune activation. Currently, glycoconjugate vaccines have been approved by the FDA for *Haemophilus influenzae*, *Neisseria meningitidis* and *Streptococcus pneumoniae*, and many others are in various stages of development. Experimental conjugate vaccines are also being developed for other antigen‐types, and for example a recombinant receptor binding domain of the SARS‐Cov‐2 spike protein conjugated to tetanus toxoid induced potent immune responses in laboratory animals.^[4]^

CRM_197_ is a widely employed carrier protein for clinically approved and experimental poly‐ and oligosaccharide conjugate vaccines.[^4^, ^5^] A large body of clinical data in different age groups support favorable immunogenicity, safety and tolerability. CRM_197_ is derived from *C. diphtheriae* toxin that has a single amino acid mutation (Gly^52^ to Glu) that greatly reduces its toxicity. The protein is composed of two domains that are covalently linked by a disulfide bridge. The A‐fragment of the native toxin inhibits protein biosynthesis by catalyzing the transfer of an ADP‐ribosyl moiety to a histidine residue of elongation factor 2 (EF‐2), which is essential for protein biosynthesis. The B‐fragment harbors a domain for binding to heparin‐binding epidermal growth factor (HB‐EGF) and another subdomain for translocation into eukaryotic cells, and is responsible for cellular uptake. The Gly^52^ to Glu mutation in fragment A results in an enzymatically inactive protein having 10^6^‐fold reduced toxicity.

An immuno‐adjuvant is commonly co‐administrated to improve the immunogenicity of glycoconjugate vaccines.^[6]^ Advances in the understanding of innate immune responses has provided opportunities to design better adjuvants.^[7]^ The innate immune system senses microbes through pattern‐recognition receptors, which include the Toll‐like receptors (TLRs) and C‐type lectin‐like receptors (CTRs) that are expressed by immune cells (*e. g*. dendritic cells (DCs)). Activation of these receptors leads to the production of cytokines that provide early defense during infection. Cytokines also regulate adaptive immunity by controlling the quantity and quality of B‐ and T‐cell activation, which in turn results in protective immune responses to pathogens. Ligands for TLRs and CTRs are attractive for the development of adjuvants.

Despite clinical successes, there are several problems associated with the use of conventional conjugate vaccines. Poly‐ and oligosaccharides are commonly conjugated to CRM_197_ and other carrier proteins by exploiting the nucleophilicity of the site chain of lysine (amine) or cysteine (sulfhydryl).^[2]^ These approaches result in heterogeneity that can compromise reproducible vaccine production. The conjugation can also mask helper T‐cell epitopes thereby reducing immunogenicity. Conventional adjuvants can improve immunogenicity, however, many exhibit toxicity resulting in adverse effects, and only a few adjuvants have been approved for clinical use.^[7]^

Covalent modification of an immunogen with an adjuvant can potentially result in more potent immune activation because cytokines will be produced locally at the site where the vaccine interacts with relevant immune cells, resulting in efficient maturation of these cells.^[8]^ It allows to reduce the adjuvant and antigen dose, thereby minimizing the risk of adverse effects. Fully synthetic immunogens composed of a peptide or glycopeptide modified by an in‐built adjuvant have been reported. Furthermore, fusion proteins of an antigen and a protein‐based immune‐potentiator such as flagellin and HSP70 have been described. However, robust methods for the controlled coupling of a small molecule adjuvant to a carrier protein are lacking.^[9]^

**Figure 1:**
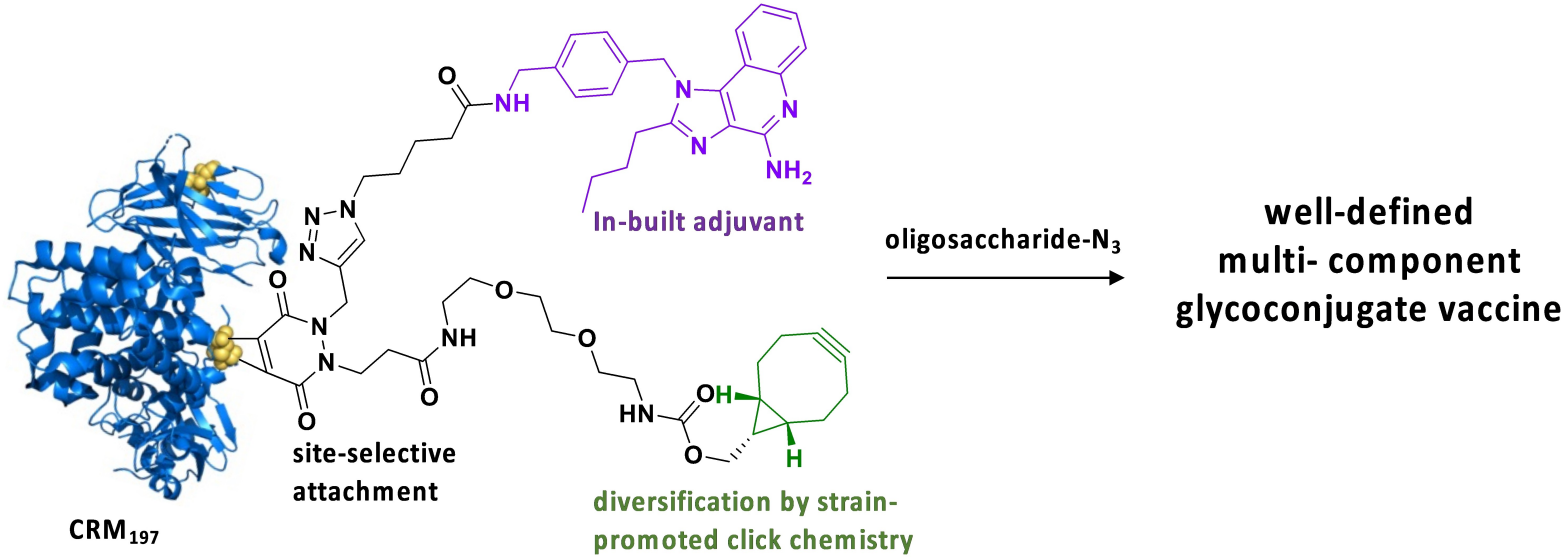
Schematic overview of controlled modification of the carrier protein CRM_197_ by pyridazinedione mediated ligation having one functional entity already installed and a second one introduced by strain promoted alkyne‐azide cycloaddition.

Here, we report a method for the controlled functionalization of CRM_197_ with an antigen and immune potentiator such as a TLR7/8 agonist or high mannoside. It is based on the selective reduction of a disulfide of CRM_197_ followed by disulfide rebridging employing a functionalized dibromopyridazinedione (Figure 1). It was found that the reduction and functionalization with a dibromopyridazinedione has to be performed by an *in‐situ* approach and is sensitive to reaction temperature and buffer choice. Various dibromopyridazinediones were investigated and it was found that a reagent having a functional entity such as a TLR7/8 agonist and a cyclooctyne for further modification is most attractive for the bi‐functionalization of CRM_197_. The use of a previously reported derivative having a cyclooctyne and alkyne gave difficulties and in particular further functionalization with Cu(I)‐catalyzed azide‐alkyne‐1,3‐dipolar‐Cycloaddition (CuAAC) resulted in degradation of the protein. Dibromopyridazinedione mediated modification of CRM_197_ appeared sensitive to steric hindrance and proceeded only partially when pre‐modified with two functional entities.

## Results and Discussion

Disulfide rebridging is an emerging approach to modify, in a controlled manner, proteins with a variety of functionalities.^[10]^ It is based on selective reduction of a disulfide bridge of a protein followed by reaction with an entity that reforms a bridge with concomitant introduction of a functional entity. An attractive approach is based on a double Michael addition of bromopyridazinedione derivatives with thiols derived from a reduced disulfide bridge.^[11]^ The two nitrogen atoms of dibromopyridazinedione can be modified by two different entities, and for example have been derivatized by a cyclooctyne for catalyst free strain‐promoted azide–alkyne cycloaddition (SPAAC)^[12]^ and a propargyl moiety for copper(I)‐catalyzed alkyne‐azide cycloaddition (CuAAC).^[13]^ The approach has been used to modify a monoclonal antibody with a fluorescent dye and a cytotoxic agent.^[14]^

**Figure 2:**
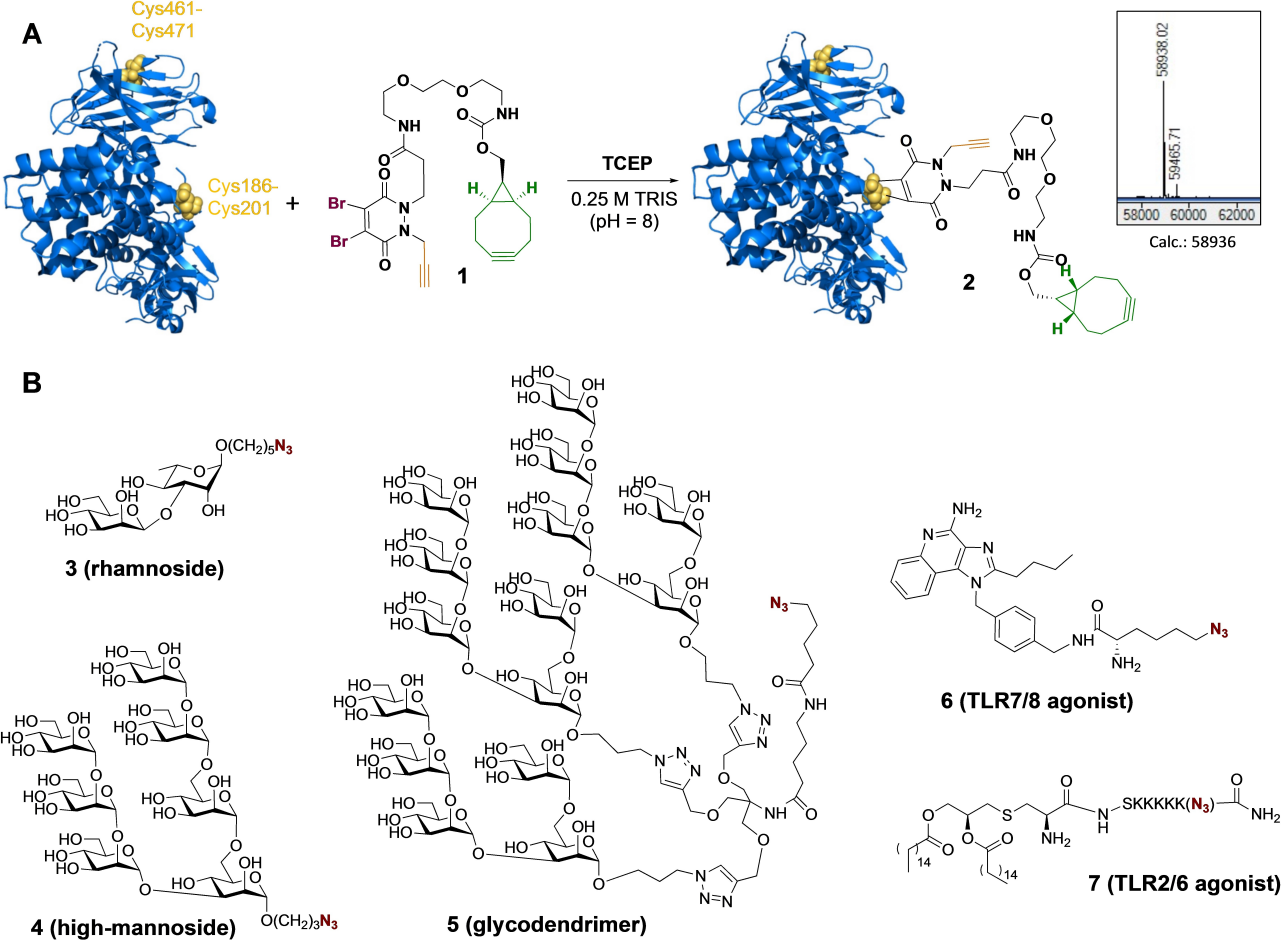
(A) Reaction of CRM_197_ (PDB 5I82, cysteine residues are marked in yellow) with pyridazinedione **1** to afford **2**. (B) Azide‐equipped glycans for conjugation to **2**.

We explored whether CRM_197_ can be modified with dibromopyridazinedione **1** for subsequent installation of an antigen and an adjuvant. CRM_197_ is composed of two subunits, which are covalently connected by disulfide Cys186−Cys201 (Figure 2 A). An additional and less accessible disulfide is present at Cys461−Cys471. It has been reported that Cys186−Cys201 can selectively be reduced by tris(2‐carboxyethyl)phosphine (TCEP) without impacting Cys461−Cys471.^[15]^ We anticipated that the resulting disulfide can be reacted with **1** to give a protein suitable for dual modification. Thus, CRM_197_ was subjected to dibromopyridazinedione modification as previously reported for IgG antibodies by first reduction with TCEP (3–10 eq.) in borate buffered saline (pH=8) for 3 h followed by buffer exchange to water by spin filtration and subsequent subjecting the resulting free thiols to **1** and further incubation for 18 h. Analysis of the reaction mixture by ESI‐QTOF mass spectrometry did not show any derivatization, and only unmodified protein was observed. A wide range of buffers, salt‐ and organic solvent additives, reagent amount, reaction times, and temperatures were screened (see Table S1). Strikingly, almost homogeneous conjugate **2** was obtained when a mixture of CRM_197_ and linker **1** were incubated in 0.25 M TRIS buffer (pH 8.0) at 0 °C, followed by the addition of TCEP and further incubation for 18 h at this temperature. Critical for an efficient transformation was *in situ* reduction of the disulfide bridge and the use of TRIS buffer. Dimerization of protein was observed when the conjugation was performed at a higher temperature than 0 °C.

Having established an efficient procedure for the modification of CRM_197_ with bifunctional linker **1**, attention was focused on dual functionalization by first performing SPAAC and then CuAAC using a range of functional molecules modified by an azide (Figure 2B). For this purpose, azide‐containing compounds **3**–**7** we prepared. Rhamnoside **3** is derived from the exopolysaccharide from *Pseudomonas aeruginosa*, and has garnered interest as a vaccine candidate.^[16]^ Furthermore, mannoside **4** has garnered interest as antigen for HIV vaccine development.^[17]^ Such a compound is also a ligand for DC‐SIGN which can facilitate uptake by dendritic cells for subsequent antigen presentation.^[18]^ Several studies have shown that the modification of tumor associated antigens with mannosides can enhance tumor immunity.^[19]^ Multivalent presentation of carbohydrate antigens is important for optimal binding to carbohydrate binding proteins such as DC‐SIGN and therefore, we prepared glycodendrimer **5** which displays three high mannosides on a trispropyne‐based scaffold (see Supporting Information for synthesis). This derivative made it also possible to examine whether bulky ligands can be installed by dibromopyridazinedione‐based bioconjugation. Compound **6** is a derivative of a small molecule agonist of TLR7/8,^[20]^ and lipopeptide **7** is an activator of TLR2^[21]^ and both compounds have attracted interest as immuno‐adjuvants.

**Figure 3:**
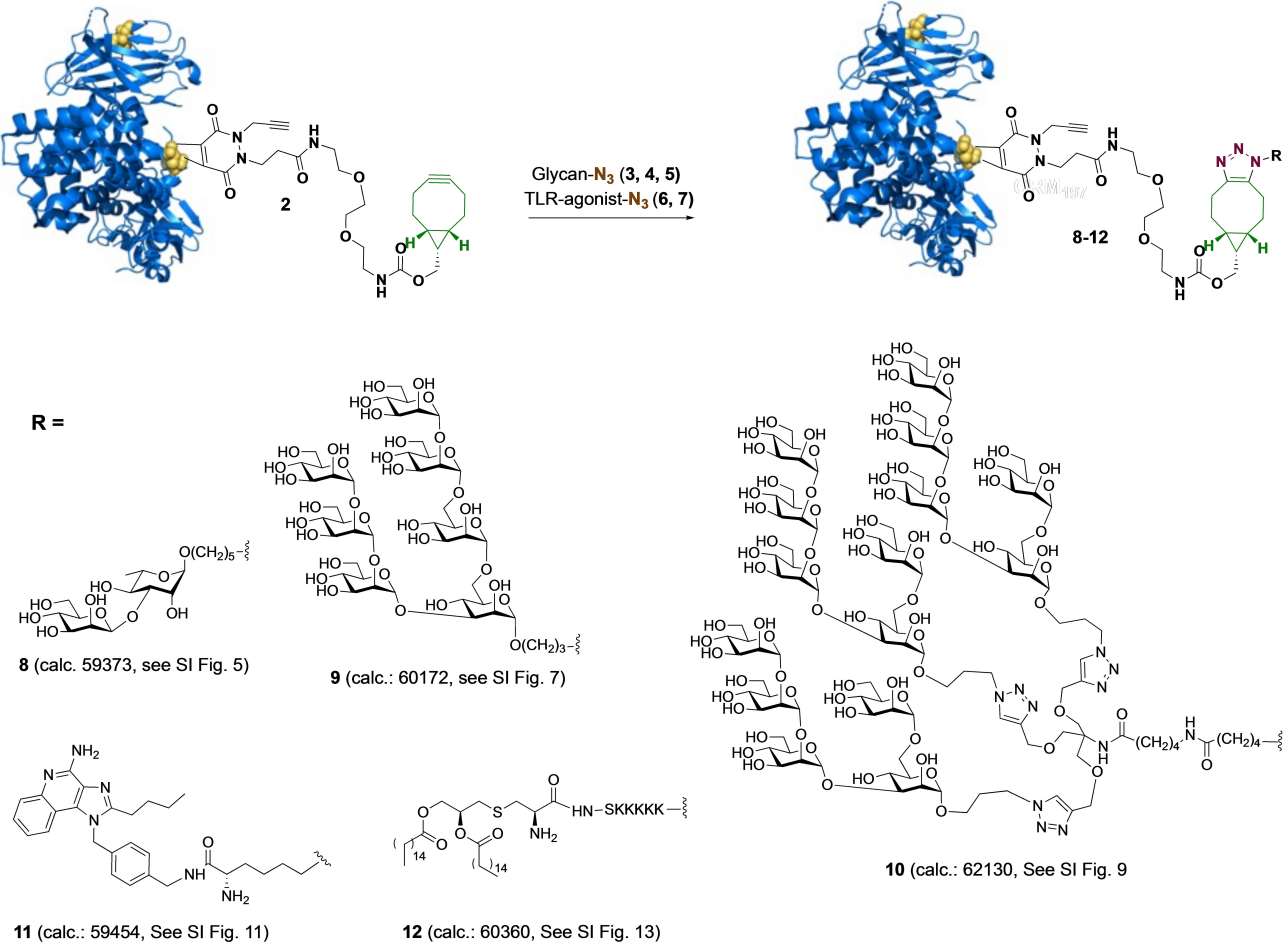
Reaction of pyridazinedione modified CRM_197_ **2** with azide‐equipped glycans **3**–**5** and TLR agonists **6** and **7**.

Relatively small azide‐bearing compounds such as disaccharide **3** and TLR‐agonist **6** readily underwent SPAAC with CRM_197_ derivative **2** in PBS buffer at 22 °C for 18 h to afford the corresponding conjugates **8** and **11** (Figure 3). In case of oligo‐mannoside **4** and dendritic mannoside **5**, the conjugation only went to completion when performed at 37 °C. TLR agonist **7** did not react under these reaction conditions which probably was due to low solubility and amphiphilic properties resulting in aggregation (see Table S2).^[14]^ The use of a mildly acidic buffer (MES, pH=6) improved the solubility of **7** and homogeneous **12** was obtained by incubation at 22 °C for 18 h.

Next, attempts were made to further functionalize conjugates **8**–**12** by CuAAC and although various reaction conditions were investigated such as Cu(II)SO_4_ in the presence of sodium ascorbate or hydroxylamine for *in‐situ* reduction to Cu(I) and various agents such as aminoguanidine and DMSO to stabilize Cu(I) or the use of CuBr, little‐ or no product formation was observed. It appeared that the employed Cu‐salts precipitated the protein, and furthermore led to the formation of various by‐products such as linker cleavage (see Table S3 for details of examined reaction conditions).

**Figure 4:**
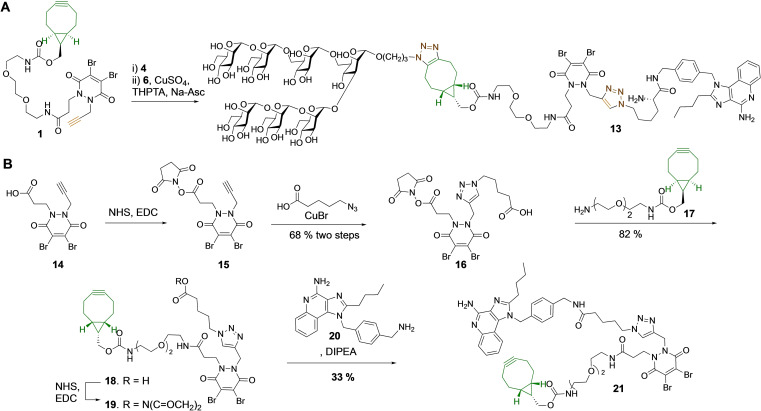
(A) Preparation of dibromopyridazinedione modified by a high mannoside and a TLR7/8 ligand. (B) Preparation of dibromopyridazinedione modified by a TLR7/8 ligand and cyclooctyne for further modification by SPAAC.

We explored two strategies to modify CRM_197_ in a controlled manner with two different functionalities avoiding a CuAAC step at the protein level. In one approach, dibromopyridazinedione **1** was sequentially subjected to SPAAC and CuAAC modification using azides **4** and **6**, respectively, to give a bifunctional linker **13** that was employed for CRM_197_ modification (Figure 4 A). In an alternative strategy, scaffold **21** was constructed that is functionalized with a TLR7/8 agonist and a bicyclo[6.1.0]nonyne (BCN) moiety for further SPAAC modification (Figure 4B).

Linker **13** could easily be prepared by first SPAAC‐mediated condensation of compound **1** with azide‐containing TLR7/8 agonist **4** followed further functionalization using azide‐modified mannoside **6** in the presence of CuBr (Figure 4A). Linker **21** was prepared by conversion of the carboxylic acid of compound **14** into an *N*‐hydroxysuccinimide (NHS)‐ester using NHS in the presence of 1‐ethyl‐3‐(−3‐dimethylaminopropyl) carbodiimide hydrochloride (EDC) to afford **15**, which was subjected to CuAAC with 5‐azidopentanoic acid in the presence of CuBr to provide, after purification by silica gel column chromatography, compound **16** in a yield of 68 % over two steps (Figure 4B). Next, the succinimide ester of **16** was conjugated to BCN‐amine **17** to give **18** in 82 % yield. Finally, the carboxylic acid of **18** was converted into an NHS‐ester (→**19)** that was conjugated to TLR7/8 agonist **20** to give dibromopyridazinedione derivative **21**.

**Figure 5:**
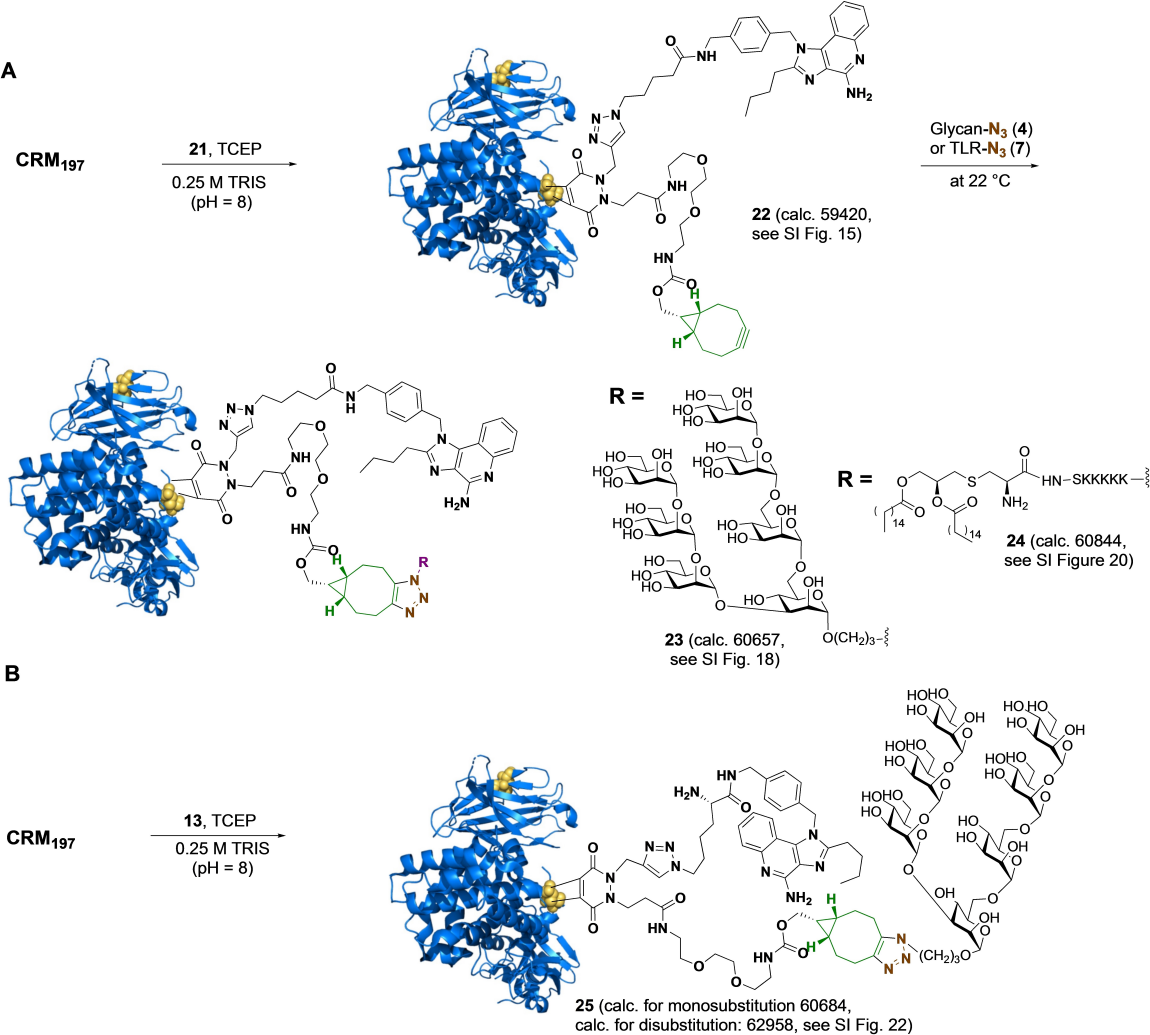
Conjugation of dibromopyridazinedione (A) **21** and (B) **13** with CRM_197_.

CRM_197_ was conjugated to dibromopyridazinediones **13** and **21** by *in situ* reduction of the disulfide bridge using TCEP followed by a tandem Michael addition using the optimized reaction conditions described above (Figure 5). In the case of **21**, almost complete mono‐substitution was achieved when the bioconjugation was performed at 22 °C for 18 h and in addition to product only a small amount of starting CRM_197_ (∼5 %) was observed (Figure 5A; Supporting Information Table 4). Interestingly, complete di‐substitution was achieved when the reaction was performed at 37 °C. It appears that at a higher temperature, Cys461−Cys471 is also reduced leading to its substitution. Conjugate **22** was subjected to SPAAC using oligomannoside **5** and TLR agonist **11**. In both cases, we observed complete conversion and conjugates **23** and **24** were obtained with high homogeneity.

Conjugation of bulkier linker **13** with CRM_197_ proceeded sluggishly when performed at 22 °C and after 18 h only ∼25 % conversion to monosubstituted conjugate **25** was observed (Figure 5B). The reaction could be accelerated by raising the reaction temperature to 37 °C, but this led also to some double substitution and the formation of other by‐products. These results indicate that a reagent such as compound **21**, having a functional entity and a cyclooctyne for further modification, is most attractive for the bis‐functionalization of CRM_197_.

**Figure 6:**
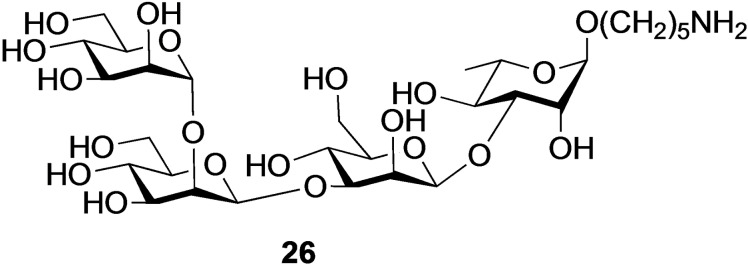
Carbohydrate antigen **26** derived from exo‐polysaccharide of *P. aeruginosa*.

We explored whether conjugate **23** can be further modified by a carbohydrate antigen such as **26** (Figure 6) by random lysine modification. Tetrasaccharide **26** is derived from the exopolysaccharide from *P. aeruginosa*, which is an attractive target for vaccine development.^[16]^ Our previous studies have shown that this tetrasaccharide can be recognized by a neutralizing monoclonal antibody, and thus may have the ability to elicit relevant antigenicity.^[16b]^ The anomeric aminopentyl moiety of compound **26** was activated by reaction with disuccinimido adipate, and was then exposed to CRM_197_ modified by a TLR7/8 agonist and a high mannoside. As a control, unmodified CRM_197_ was also subjected to random conjugation. After a reaction time of 16 h, the protein conjugates were purified by spin‐filtration and analyzed by MALDI‐TOF MS, which in each case revealed a glycan/protein ratio of ∼7 (Figure S23). It is the expectation that the mannoside of the multifunctional conjugate will facilitate uptake by dendritic cells and the TLR7/8 agonist will induce pro‐inflammatory cytokines which are expected to enhance the antigenicity of *P. aeruginosa* derived tetrasaccharide.

Finally, we assessed the stability of pyridazinedione‐modified CRM_197_ in the presence of 5 μM and 5 mM glutathione (GSH) resembling the concentration of this reducing agent in blood serum and endosomes, respectively.^[16]^ Thus, conjugate **22** was incubated in PBS buffer (pH=7.4) in the presence of 5 μM glutathione (GSH) at 37 °C to mimic bloodserum conditions. Gratifyingly, after an incubation time of 16 h analysis by mass spectrometry showed only the presence of the starting conjugate (Figure S24). On the other hand, treatment of conjugate **22** in PBS (pH=6.0) in the presence of 5 mM GSH led within a period of 5 h to complete linker cleavage and in this case MS analysis showed the presence of mainly unmodified CRM_197_ (58,410 kDa, Figure S25). These observations indicate that pyridazinedione‐modified CRM_197_ is stable in serum but after cell internalization readily sheds it ligands. The latter will facilitate release of the TLR7/8 ligand for interaction with its intracellular receptor and immune cell activation.

## Conclusions

In this study, we present a strategy for the site‐selective derivatization of the widely employed carrier protein CRM_197_ ^[5]^ by two different functionalities. It is based on the selective reduction of a disulfide bridge of CRM_197_ followed by reaction with appropriately functionalized dibromopyridazinediones. The latter type of reagent has been employed for the modification of peptides and IgG antibodies,[^14^, ^22^] and in this study we explored whether its utility can be extended to the modification of other proteins. Although CRM_197_ could be modified in a controlled manner by dibromopyridazinedione derivatives, it was critical to control the reaction conditions and required *in situ* reduction of one of the disulfide bridges of the protein in TRIS buffer at pH=8 with concomitant reaction with a dibromopyridazinedione. In this way, protein aggregation and other modifications such as the formation of TCEP conjugates could be avoided. Side product formation with free cysteines is a general problem of rebridging protein modification strategies,^[10a]^ and the *in situ* reduction approach described here may provide a general solution. The conjugation is sensitive to the bulkiness of the introduced functionalities and the most favorable approach is based on the use of a dibromopyridazinedione modified by one functional entity such as a TLR ligand and a cyclooctyne for further modification after reaction with CRM_197_. The approach makes it possible to functionalize CRM_197_ with both an antigen and an adjuvant. Future studies will focus on examining antigenicity of the functionalized CRM_197_ conjugates.

## Experimental Section

Experimental details can be found in the Supporting Information. The Supporting Information (PDF) contains synthetic procedures and general procedures for protein modification (including Tables S1–S3 and Figures S1–S25).

## Conflict of interest

The authors declare no conflict of interest.

1

## Supporting information

As a service to our authors and readers, this journal provides supporting information supplied by the authors. Such materials are peer reviewed and may be re‐organized for online delivery, but are not copy‐edited or typeset. Technical support issues arising from supporting information (other than missing files) should be addressed to the authors.

Supporting Information

## Data Availability

The data that support the findings of this study are available in the supplementary material of this article.

## References

[1] P. M. Moyle , I. Toth , ChemMedChem 2013, 8, 360–376. 23316023 10.1002/cmdc.201200487

[2a] F. Berti , R. Adamo , Chem. Soc. Rev. 2018, 47, 9015–9025; 30277489 10.1039/c8cs00495a

[2b] R. Rappuoli , Sci. Transl. Med. 2018, 10, eaat4615; 30158151 10.1126/scitranslmed.aat4615

[2c] S. Lang , X. Huang , Front. Chem. 2020, 8, 284. 32351942 10.3389/fchem.2020.00284 PMC7174737

[3] M. L. Hecht , P. Stallforth , D. V. Silva , A. Adibekian , P. H. Seeberger , Curr. Opin. Chem. Biol. 2009, 13, 354–359. 19560394 10.1016/j.cbpa.2009.05.127

[4] Y. Valdes-Balbin , D. Santana-Mederos , L. Quintero , S. Fernandez , L. Rodriguez , B. Sanchez Ramirez , R. Perez-Nicado , C. Acosta , Y. Mendez , M. G. Ricardo , T. Hernandez , G. Bergado , F. Pi , A. Valdes , T. Carmenate , U. Ramirez , R. Oliva , J. P. Soubal , R. Garrido , F. Cardoso , M. Landys , H. Gonzalez , M. Farinas , J. Enriquez , E. Noa , A. Suarez , C. Fang , L. A. Espinosa , Y. Ramos , L. J. Gonzalez , Y. Climent , G. Rojas , E. Relova-Hernandez , Y. Cabrera Infante , S. L. Losada , T. Boggiano , E. Ojito , K. Leon , F. Chiodo , F. Paquet , G. W. Chen , D. G. Rivera , D. Garcia-Rivera , V. Verez Bencomo , ACS Chem. Biol. 2021, 16, 1223–1233. 34219448 10.1021/acschembio.1c00272

[5] M. Broker , P. Costantino , L. DeTora , E. D. McIntosh , R. Rappuoli , Biologicals 2011, 39, 195–204. 21715186 10.1016/j.biologicals.2011.05.004

[6a] B. Pulendran , R. Ahmed , Nat. Immunol. 2011, 12, 509–517; 21739679 10.1038/ni.2039 PMC3253344

[6b] S. M. Levitz , D. T. Golenbock , Cell 2012, 148, 1284–1292. 22424235 10.1016/j.cell.2012.02.012 PMC3308125

[7] B. Pulendran , P. S. Arunachalam , D. T. O'Hagan , Nat. Rev. Drug Discovery 2021, 20, 454–475. 33824489 10.1038/s41573-021-00163-y PMC8023785

[8a] Q. Li , Z. Guo , Molecules 2018, 23, 1583; 10.3390/molecules23071583 PMC6100623 29966261

[8b] Z. Xu , P. M. Moyle , Bioconjugate Chem. 2018, 29, 572–586; 10.1021/acs.bioconjchem.7b00478 28891637

[8c] Y. Manabe , T. C. Chang , K. Fukase , Drug Discovery Today Technol. 2020, 37, 61–71. 10.1016/j.ddtec.2020.11.006 34895656

[9a] M. S. Duthie , H. P. Windish , C. B. Fox , S. G. Reed , Immunol. Rev. 2011, 239, 178–196; 21198672 10.1111/j.1600-065X.2010.00978.x PMC5872835

[9b] T. Y. Wu , M. Singh , A. T. Miller , E. De Gregorio , F. Doro , U. D'Oro , D. A. Skibinski , M. L. Mbow , S. Bufali , A. E. Herman , A. Cortez , Y. Li , B. P. Nayak , E. Tritto , C. M. Filippi , G. R. Otten , L. A. Brito , E. Monaci , C. Li , S. Aprea , S. Valentini , S. Calabromicron , D. Laera , B. Brunelli , E. Caproni , P. Malyala , R. G. Panchal , T. K. Warren , S. Bavari , D. T. O'Hagan , M. P. Cooke , N. M. Valiante , Sci. Transl. Med. 2014, 6, 263ra160. 10.1126/scitranslmed.3009980 25411473

[10a] S. L. Kuan , T. Wang , T. Weil , Chem. Eur. J. 2016, 22, 17112–17129; 27778400 10.1002/chem.201602298 PMC5600100

[10b] P. Ochtrop , C. P. R. Hackenberger , Curr. Opin. Chem. Biol. 2020, 58, 28–36. 32645576 10.1016/j.cbpa.2020.04.017

[11] V. Chudasama , M. E. Smith , F. F. Schumacher , D. Papaioannou , G. Waksman , J. R. Baker , S. Caddick , Chem. Commun. 2011, 47, 8781–8783. 10.1039/c1cc12807h PMC3379626 21738916

[12] J. Dommerholt , F. Rutjes , F. L. van Delft , Top. Curr. Chem. 2016, 374, 16. 10.1007/s41061-016-0016-4 PMC5480410 27573141

[13] V. K. Tiwari , B. B. Mishra , K. B. Mishra , N. Mishra , A. S. Singh , X. Chen , Chem. Rev. 2016, 116, 3086–3240. 26796328 10.1021/acs.chemrev.5b00408

[14] A. Maruani , M. E. Smith , E. Miranda , K. A. Chester , V. Chudasama , S. Caddick , Nat. Commun. 2015, 6, 6645. 25824906 10.1038/ncomms7645 PMC4389247

[15a] G. Stefanetti , Q. Y. Hu , A. Usera , Z. Robinson , M. Allan , A. Singh , H. Imase , J. Cobb , H. Zhai , D. Quinn , M. Lei , A. Saul , R. Adamo , C. A. MacLennan , F. Micoli , Angew. Chem. Int. Ed. 2015, 54, 13198–13203; 10.1002/anie.201506112 PMC4648054 26350581

[15b] F. Carboni , A. Kitowski , C. Sorieul , D. Veggi , M. C. Marques , D. Oldrini , E. Balducci , B. Brogioni , L. Del Bino , A. Corrado , F. Angiolini , L. Dello Iacono , I. Margarit , M. R. Romano , G. J. L. Bernardes , R. Adamo , Chem. Sci. 2022, 13, 2440–2449. 35310500 10.1039/d1sc01928g PMC8864718

[16a] A. Digiandomenico , P. Warrener , M. Hamilton , S. Guillard , P. Ravn , R. Minter , M. M. Camara , V. Venkatraman , R. S. Macgill , J. Lin , Q. Wang , A. E. Keller , J. C. Bonnell , M. Tomich , L. Jermutus , M. P. McCarthy , D. A. Melnick , J. A. Suzich , C. K. Stover , J. Exp. Med. 2012, 209, 1273–1287; 22734046 10.1084/jem.20120033 PMC3405507

[16b] H. Li , K. F. Mo , Q. Wang , C. K. Stover , A. Digiandomenico , G. J. Boons , Chem. Eur. J. 2013, 19, 17425–17431. 24248772 10.1002/chem.201302916

[17a] C. N. Scanlan , J. Offer , N. Zitzmann , R. A. Dwek , Nature 2007, 446, 1038–1045; 17460665 10.1038/nature05818

[17b] L. X. Wang , Curr. Opin. Chem. Biol. 2013, 17, 997–1005; 24466581 10.1016/j.cbpa.2013.10.001 PMC4100479

[17c] R. Pantophlet , N. Trattnig , S. Murrell , N. Lu , D. Chau , C. Rempel , I. A. Wilson , P. Kosma , Nat. Commun. 2017, 8, 1601; 29150603 10.1038/s41467-017-01640-y PMC5693931

[17d] G. E. Seabright , K. J. Doores , D. R. Burton , M. Crispin , J. Mol. Biol. 2019, 431, 2223–2247. 31028779 10.1016/j.jmb.2019.04.016 PMC6556556

[18] B. Lepenies , J. Lee , S. Sonkaria , Adv. Drug Delivery Rev. 2013, 65, 1271–1281. 10.1016/j.addr.2013.05.007 23727341

[19] S. Duinkerken , R. E. Li , F. J. van Haften , T. D. de Gruijl , F. Chiodo , S. T. T. Schetters , Y. van Kooyk , Curr. Opin. Chem. Biol. 2019, 53, 167–172. 31678713 10.1016/j.cbpa.2019.10.001

[20] L. Ganapathi , S. Van Haren , D. J. Dowling , I. Bergelson , N. M. Shukla , S. S. Malladi , R. Balakrishna , H. Tanji , U. Ohto , T. Shimizu , S. A. David , O. Levy , PLoS One 2015, 10, e0134640. 26274907 10.1371/journal.pone.0134640 PMC4537157

[21] V. Lakshminarayanan , P. Thompson , M. A. Wolfert , T. Buskas , J. M. Bradley , L. B. Pathangey , C. S. Madsen , P. A. Cohen , S. J. Gendler , G. J. Boons , Proc. Natl. Acad. Sci. USA 2012, 109, 261–266. 22171012 10.1073/pnas.1115166109 PMC3252914

[22] C. Bahou , D. A. Richards , A. Maruani , E. A. Love , F. Javaid , S. Caddick , J. R. Baker , V. Chudasama , Org. Biomol. Chem. 2018, 16, 1359–1366. 29405223 10.1039/c7ob03138f PMC6058253

