# Supplementary material for: Site‐Specific Multi‐Functionalization of the Carrier Protein CRM197 by Disulfide Rebridging for Conjugate Vaccine Development
Source: Chembiochem. 2022 Sep 29:e202200408. Online ahead of print. doi: 10.1002/cbic.202200408 (PMC9538913; doi:10.1002/cbic.202200408)
Supplement: Supplementary file 1 — Supporting Information [file CBIC-9999-0-s001.pdf]

ChemBioChem

Supporting Information

Site-Specific Multi-Functionalization of the Carrier Protein CRM_{197} by Disulfide Rebridging for Conjugate Vaccine Development

Nino Trattnig, Zeshi Li, Gerlof P. Bosman, Paul Kosma, and Geert-Jan Boons*

Contents

1. General methods 2
2. Synthetic procedures 2
2.1. Synthesis of clickable probes 2
2.2. Synthesis of Monofunctionalized linker 7
2.3. Synthesis of bifunctionalized linker 11
3. Analyctical data of azide and linker compounds 13
4. Site-selective protein conjugation 29
4.1. Conjugation of CRM_{197} with linker 1 29
4.1.1. Installation of linker 1 on CRM_{197} 29
4.1.2. Synthesis of conjugate 8 33
4.1.3. Synthesis of conjugate 9 34
4.1.4. Synthesis of conjugate 10 35
4.1.5. Synthesis of conjugate 11 37
4.1.6. Synthesis of conjugate 12 38
4.1.7. Attempts with CuAAc and scaffold 11 40
4.2. Conjugation of CRM_{197} with monofunctionalized linker 21 42
4.2.1. Installation of the monofunctionalized linker 21 on CRM_{197} 42
4.2.2. Synthesis of conjugate 23 44
4.2.3. Synthesis of conjugate 24 45
4.3. Conjugation of CRM_{197} with difunctionalized linker 13 47
5. Random conjugation 49
6. Stability studies with conjugate 22 50
7. References 52

1. General methods

All purchased chemicals were used without further purification unless stated otherwise. CRM_{197} was purchased from Scarab Genomics. Solvents were dried over activated 4 Å molecular sieves. Aqueous solutions of salts were saturated unless stated otherwise. Concentration of organic solutions was performed under reduced pressure at 40 °C. Thin layer chromatography (TLC) was performed on silica gel $60F_{254}$ with detection by UV light (254 nm) and staining by p-anisaldehyde solution, followed by heating for visualization. Column chromatography was performed on silica gel G60 (0.040 – 0.063 mm). Analytical HPLC was performed on a Shimadzu HPLC system coupled to a ELSD detector (Alltech3300) using ZIC-HILIC (SeQuant, 5 µm), C5 (Phenomenex Jupiter, 5 µm) or a C18 column (Zorbax Eclipse XDB, 5 µm). Preparative HPLC was performed on a Puriflash4125 using HILIC, C4 or C18 columns. NMR spectra were recorded on a Bruker Avance III 600 instrument (600.22 MHz for ^{1}H, 150.93 MHz for ^{13}C) using standard Bruker NMR software. Chemical shifts are reported in parts per million (ppm). NMR data is presented as: chemical shift, multiplicity (where s = singlet, d = doublet, t = triplet, dd = doublet of doublets, m = multiplet), the coupling constant in Hertz (Hz), and allocation. ^{1}H spectra were referenced 7.26 ppm ($CDCl_3$), 3.31 (MeOD), 2.05 [$(CD_3)_2CO$] and 0.00 (D_2O, external calibration to 2,2-dimethyl-2-silapentane-5-sulfonic acid) ppm. ^{13}C spectra were referenced to 77.16 ($CDCl_3$), 49.00 (MeOD), 29.8 [$(CD_3)_2CO$] and 67.40 (D_2O, external calibration to 1,4-dioxane) ppm. Assignments were based on COSY, HSQC and HMBC data. HRMS data were obtained on a Micromass Q-TOF Ultima Global instrument. Protein mass determination was performed on an Agilent 6560 drift tube IMS (DTIMS)- quadrupole time-off-flight (Q-TOF) MS instrument using a UPLC column (Waters Bioresolve -RP.mAb, 2.7 µm, 2.1×50 mm). MALDI-TOF measurements were performed on a Bruker Autoflex and concentration of Proteins was determined with a DeNovix Nanodrop spectrometer, using for CRM_{197}-derivatives an E1 % value of 10.7.

2. Synthetic procedures

2.1. Synthesis of clickable probes

5-Azido-1-pentyl β-D-mannopyranosyl-(1→3)-α-L-rhamnopyranose (3)

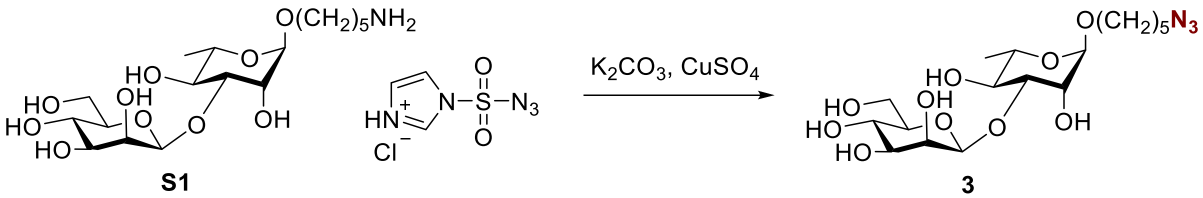

S1 **3**

S1 (4.0 mg; 9.7 µmol), K_2CO_3 (13.4 mg; 97 µmol) and imidazole-1-sulfonyl azide hydrochloride[1] (8.2 mg; 39 µmol) were dissolved in a mixture of MeOH/water (1 mL; 7/3), followed by the addition of $CuSO_4 \times 5\ H_2O$ (0.2 mg; 1 µmol). It was stirred at 37 °C for 18 h and the solvent was then evaporated *in vacuo*. The crude product was purified by HPLC (semi-preparative ZIC-HILIC column; MeCN/25 mM aqu. NH_4COO (9/1 → 4/6) to obtain compound **3** (1.8 mg; 42 %) as colorless amorphous solid. ^{1}H NMR (600 MHz, MeOD): δ = 4.81 (d, *J* = 0.8 Hz, 1 H, H-1'), 4.69 (d, *J* = 1.6 Hz, 1 H, H-1), 4.09 (dd, *J* = 3.2, 1.6 Hz, 1 H, H-2), 4.01 (dd, *J* = 3.4, 0.8 Hz, 1 H, H-2'), 3.87 (dd, *J* = 11.8, 2.4 Hz, 1 H, H-6a'), 3.78 (dd, *J* = 9.5, 3.3 Hz, 1 H, H-3), 3.75 (dd, *J* = 11.8, 5.4 Hz, 1 H, H-6b'), 3.71 (dt, *J* = 9.5, 6.4 Hz, 1 H, OCH_aCH_2), 3.67-3.61 (m, 2 H, H-5, H-4'), 3.51 (t, *J* = 9.5 Hz, 1 H, H-4), 3.47-3.42 (m, 2 H, H-3'/OCH_bCH_2), 3.33-3.31 (m, 2 H, CH_2N_3), 3.24 (ddd, *J* =9.6, 5.4, 2.5 Hz, 1 H, H-5'), 1.68-1.61 (m, 4 H, $OCH_2CH_2CH_2CH_2$), 1.53-1.46 (m, 2 H, $OCH_2CH_2CH_2$), 1.28 (d, *J* = 6.5 Hz, 3 H, H-6); ^{13}C NMR (150 MHz, MeOD): δ = 103.1 (C-1'), 101.2 (C-1), 81.6 (C-3), 78.1 (C-5'), 75.2 (C-3'), 73.0 (C-4), 72.5 (C-2'), 72.2 (C-2), 69.7 (C-5), 68.4 (OCH_2CH_2), 68.3 (C-4'), 62.7 (C-6'), 52.4 (CH_2N_3), 30.1 ($OCH_2CH_2CH_2$), 29.7 ($CH_2CH_2N_3$), 24.1 ($OCH_2CH_2CH_2$), 18.0 (C-6); HRMS (ESI-TOF) m/z: $[M+NH_4]^+$ Calcd for $C_{17}H_{35}N_4O_{10}$ 455.2348 Found: 455.2358.

Dendrimer 5

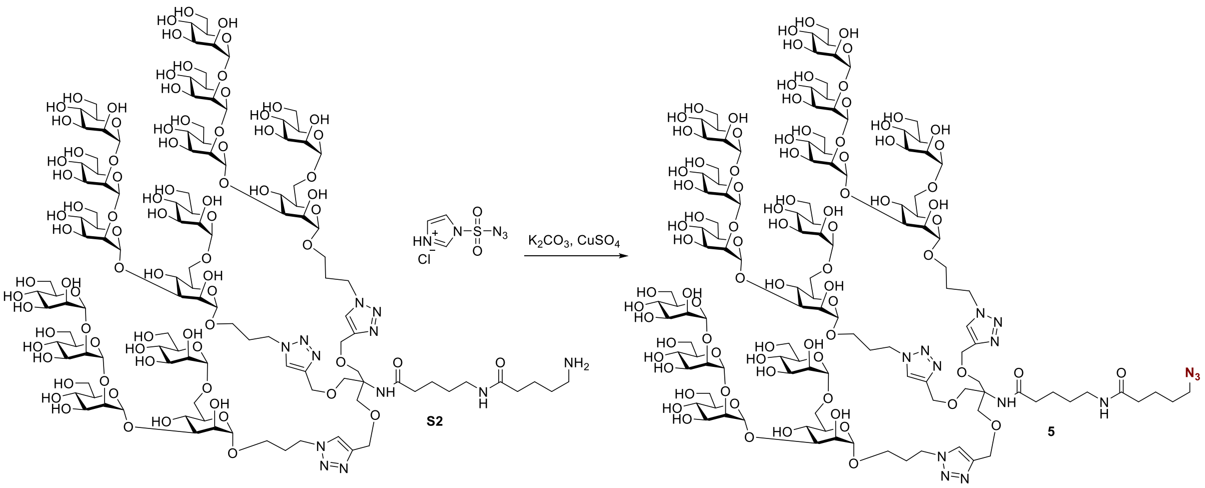

S2 (1.0 mg; 0.3 µmol), K_2CO_3 (3.5 mg; 25 µmol) and imidazole-1-sulfonyl azide hydrochloride[1] (2.0 mg; 9.5 µmol) were dissolved in a mixture of MeOH/water (0.4 mL; 1/1) followed by the addition of $CuSO_4 \times 5\ H_2O$ (0.01 mg; 0.04 µmol). It was stirred at 22 °C for 18 h and the solvent was then removed *in vacuo*. The crude compound was purified with HPLC (10 sequential runs on analytical ZIC-HILIC; H_2O/MeCN with 0.1 % AcOH (2/8 →7/3) to obtain compound **5** (1.0 mg; quant.) as colorless amorphous solid. ^{1}H NMR (600 MHz, D_2O): δ = 7.98 (s, 3 H, triazole H), 5.34, 5.30, 5.04, 4.81, 4-74 (5 × s, 5 × 3 H, anomeric H), 4.57 (s, 2 H), 4.54-4.48 (m, 2 H), 4.13-4.04 (m, 12 H), 4.00-3.58 (m, 98 H), 3.45-3.39 (m, 3 H), 3.29 (t, *J* = 6.3 Hz, 2 H), 3.10 (t, *J* = 6.6 Hz, 2 H), 2.24-2.13 (m, 10 H), 1.64-1.37 (m, 12 H)

ppm; ^{13}C NMR (Anomeric carbons taken from HSQC, D_2O): δ = 102.2, 100.8, 100.4, 99.8, 99.3; HRMS (ESI-TOF) m/z: $[M+2H]^{2+}$ Calcd for $C_{122}H_{206}N_{14}O_{83}$ 1597.6159 Found: 1597.6118

Synthesis of TLR7/8 probe

(9H-fluoren-9-yl)methyl (S)-(1-((4-((4-amino-2-butyl-1H-imidazo[4,5-c]quinolin-1-yl)methyl)benzyl)amino)-6-azido-1-oxohexan-2-yl)carbamate (S4)

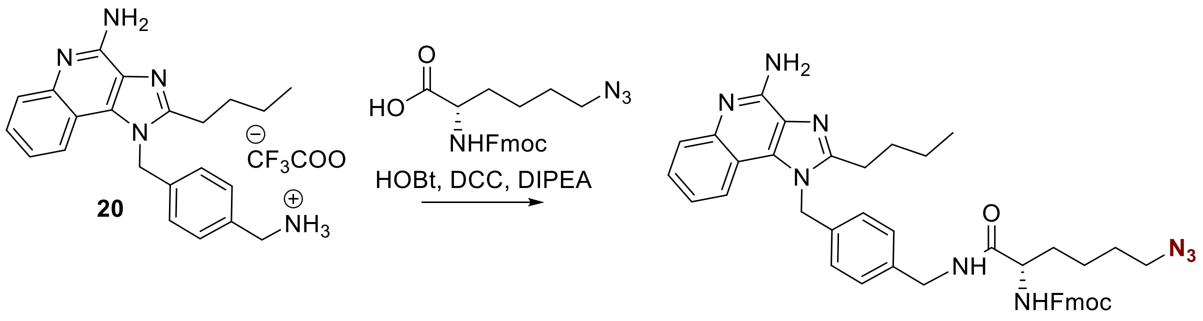

S3

6-azido *L*-(Fmoc)-Lysine (22 mg; 56 µmol) was dissolved in dry DMF (1 mL) under Argon followed by the addition of DCC (11 mg; 56 µmol) and HOBt (8 mg; 56 µmol). It was stirred for 10 min at 22 °C before TLR-agonist **S3**[2] (20 mg; 51 µmol) and DIPEA (28 µL; 167 µmol) were added. Stirring was continued at that temperature for 20 h until TLC indicated full conversion of **20**. The solvent was removed *in vacuo* and the crude product was purified with silica flash chromatography (DCM/MeOH/NEt_3 100/3/0.2 → 100/7/0.2) and compound **S3** (15 mg; 40 %) was obtained as colorless amorphous solid. R_f = 0.54 (DCM/MeOH/NEt_3=10/1/0.1); ^{1}H-NMR (600 MHz; $CDCl_3$): δ 7.80 (d, *J* = 8.2 Hz, 1 H, Ar), 7.74 (d, *J* = 3.4 Hz, 2 H, Ar), 7.66 (d, *J* = 8.2 Hz, 1 H, Ar), 7.53 (d, *J* = 10.7 Hz, 2 H, Ar), 7.42 (ddd, *J* = 8.1, 7.2, 1.0 Hz, 1 H, Ar), 7.37 (t, *J* = 7.4 Hz, 2 H, Ar), 7.33-7.25 (m, 2 H, Ar), 7.19 (bd, *J* = 7.4 Hz, 2 H, Ar), 7.11 (ddd, *J* = 8.1, 1.0, 7.2 Hz, 1 H, Ar), 6.97 (d, *J* = 8.2 Hz, 2 H, Ar), 6.33 (bs, 1 H, NH), 5.67 (s, 2 H, CH_2NHCO), 5.29 (bs, 1 H, NH), 4.46-4.34 (m, 4 H, NCH_2Ar, CH_2 from Fmoc), 4.17 (t, *J* = 6.7 Hz, 1 H, CH from Fmoc), 4.12 (dd, *J* = 12.8, 6.7 Hz, 1 H, *CH*NHFmoc), 3.30-3.18 (m, 2 H, CH_2N_3), 2.84 (t, *J* = 7.8 Hz, 2 H, CH_2-Propyl), 1.92-1.85 (m, 1 H, NHFmocCHCH_a), 1.78 (dt, *J* = 15.4, 7.7 Hz, 2 H, $CH_3CH_2CH_2$), 1.69-1.50 (m, 3 H, $N_3CH_2CH_2$/NHFmocCHCH_a), 1.46-1.35 (m, 4 H, CH_3CH_2/$CH_2CH_2CH_2N_3$), 0.91 (t, *J* = 7.2 Hz, 3 H, CH_3) ppm; ^{13}C-NMR (150 MHz; $CDCl_3$): δ 171.5 (NH*CO*CH), 156.3 ($NHCO_2$), 153.9, 151.1, 144.5, 143.7, 143.6, 141.3, 137.8, 134.7, 133.9, 128.4, 127.8, 127.1, 126.8, 126.7, 125.9, 125.0, 124.9, 122.2, 120.0, 119.6, 115.1 (Ar), 67.0 (CH_2 from Fmoc), 54.9 (*CH*NHFmoc), 51.1 (CH_2N_3), 48.5 (CH_2NHCO), 47.1 (*CH* from Fmoc), 43.0 (NCH_2Ar), 31.9 (NHFmocCHCH_2), 29.9 ($CH_2CH_2CH_3$), 28.4 ($CH_2CH_2N_3$), 27.1 (CH_2Pr), 22.7, 22.5 (2 C, CH_3CH_2/$CH_2CH_2CH_2N_3$), 13.8 (CH_3) ppm; HRMS (ESI-TOF): m/z $[M+H]^+$ calcd. for $C_{43}H_{46}N_9O_3$: 736.3718; found 736.3724

(S)-(1-((4-((4-amino-2-butyl-1H-imidazo[4,5-c]quinolin-1-yl)methyl)benzyl)amino)-6-azido-1-oxohexan-2-yl)carbamate (6)

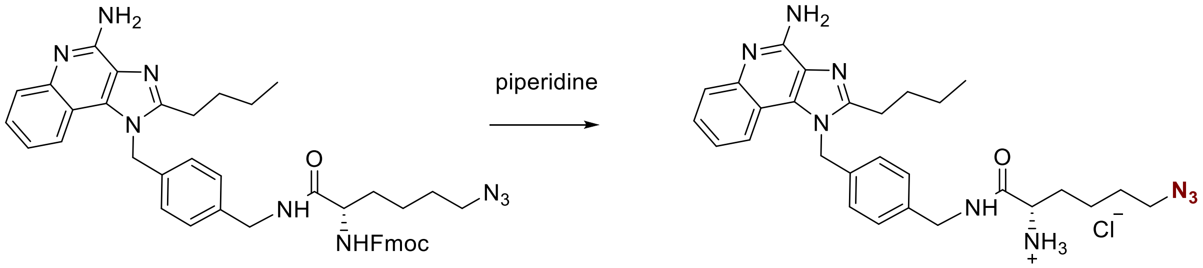

Compound **S3** (7.0 mg; 9.5 µmol) was dissolved in a mixture of DMF/piperidine (1 mL; 4/1) and after 3 h stirring at 22 °C the solvent was removed *in vacuo*. Then, it was coevaporated with toluene two times and the remaining oil was dissolved in dry EtOH (1 mL) followed by the addition of HCl (80 µL; 1 M in EtOH). It was stirred for one more hour to form the hydrochloride and the solvent was removed once more *in vacuo*. The crude product was purified by preparative HPLC (C4 column; water/MeCN = 9/1 → 1/9) to obtain product **6** (4.0 mg; 76 %) as colorless amorphous oil. R_f = 0.14 (DCM/MeOH/NEt_3=20/1/0.1); ^{1}H-NMR (600 MHz; MeOD): δ 7.88 (dd, *J* = 8.3, 1 Hz, 1 H, Ar), 7.70 (dd, *J* = 8.3, 0.5 Hz, 1 H, Ar), 7.52 (ddd, *J* = 8.1, 7.3, 0.9 Hz, 1 H, Ar), 7.31 (d, *J* = 8.2 Hz, 2 H, Ar), 7.23 (t, *J* = 7.4 Hz, 1 H, Ar), 7.07 (d, *J* = 10.0 Hz, 2 H, Ar), 5.92 (s, 2 H, CH_2NHCO), 4.46 (d, *J* = 15.6 Hz, 1 H, NCH_aAr), 4.33 (d, *J* = 15.6 Hz, 1 H, NCH_aAr), 3.71 (t, *J* = 6.2 Hz, 1 H, *CH*NH_2), 3.21 (t, *J* = 6.7, 2 H, CH_2N_3), 2.99 (t, *J* = 7.6 Hz, 2 H, CH_2-Propyl), 1.88-1.68 (m, 4 H, NH_2CHCH_2/$CH_3CH_2CH_2$), 1.56-1.34 (m, 6 H, CH_3CH_2/$CH_2CH_2CH_2N_3$), 0.95 (t, *J* = 7.2 Hz, 3 H, CH_3) ppm; ^{13}C-NMR (150 MHz; MeOD): δ 169.9 (NH*CO*CH), 158.9, 150.5, 139.7, 137.5, 135.6, 131.7, 130.9, 129.8, 126.9, 126.3, 125.9, 122.9, 119.8, 114.3 (Ar), 54.3 (*CH*NH_2), 52.0 (CH_2N_3), 49.8 (CH_2NHCO), 43.7 (NCH_2Ar), 32.2 (NH_2CHCH_2), 30.3 ($CH_2CH_2CH_3$), 29.3 ($CH_2CH_2N_3$), 27.7 (CH_2Pr), 23.3, 23.0 (2 C, CH_3CH_2/$CH_2CH_2CH_2N_3$), 14.0 (CH_3) ppm; HRMS (ESI-TOF): m/z $[M+2H]^{2+}$ calcd. for $C_{28}H_{35}N_9O$: 257.6555; found 257.6561

Synthesis of TLR1/2 probe

Automated solid-phase peptide synthesis

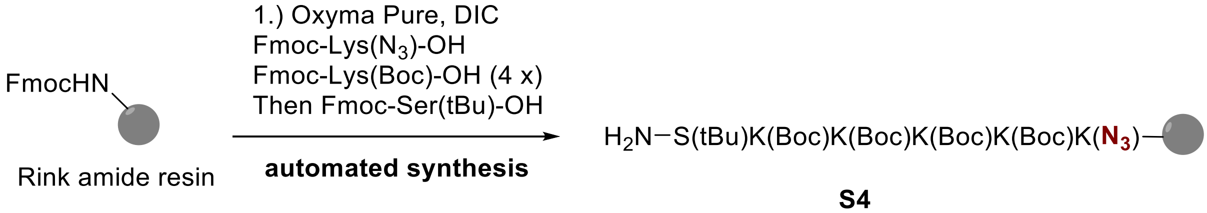

The azide equipped hexapeptide was synthesized *via* Fmoc-solid-phase Peptide Synthesis (SPPS) with a microwave-assisted peptide synthesizer (CEM HT12 Liberty Blue). Rink Amide AM resin (25 µmol)

was swollen in 10 mL of a 1:1 mixture of DMF/DCM for 5 min, drained, and then treated with piperidine (20 % in DMF; 10 mL) for 65 seconds at 90 °C, followed by draining, and washing with DMF (3 × 5 mL). The resin was then treated with the Fmoc protected Amino acid (0.2 mol/L, 0.625 mL, 5 eq), DIC (1 mol/L, 0.25 mL, 10 eq) and Oxyma Pure (1 mol/L, 0.125 mL, 5 eq) in DMF (4 mL) at 76 °C for 15 s before the temperature was increased to 90 °C for an additional 110 s following by draining of the solvent. Fmoc was cleaved as described above and the resin was treated with the next amino acid. After full assembly of the hexapeptide, a small amount of the resin was treated with 1 drop of TFA in MeCN/water (1/1), it was stirred for 1 h and MALDI-TOF analysis of the supernatant revealed full conversion to the desired peptide.

Manual assembly of the TLR1/2 agonist

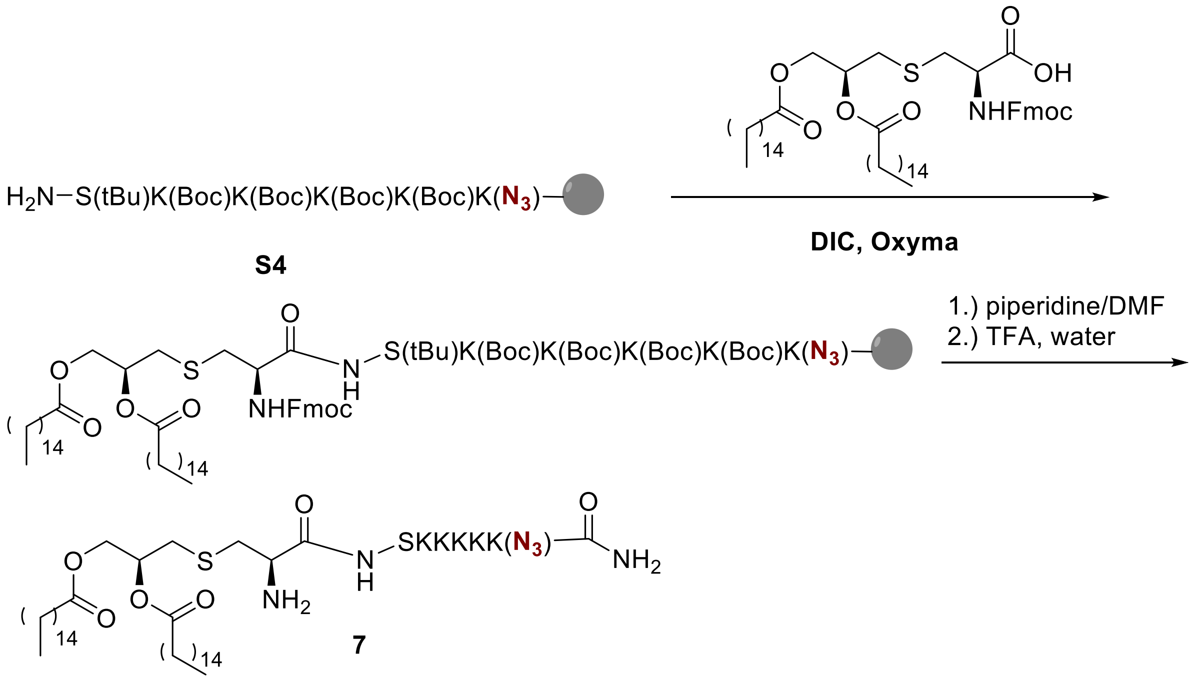

Commercially available Pam$_2$Cys (28 mg; 31 µmol) was dissolved in dry DMF (1 mL) followed by the addition of Oxyma Pure (4.4 mg; 31 µmol) and DIC (7.2 µL; 47 µmol) and it was stirred for 3 min at 22 °C. Subsequently, the resin bound peptide (ca. 16 µmol) was added and it was stirred for 16 h more. Then, the resin was filtered, washed with DMF (5 times) and stirred in 20 % piperidine (1 mL; in DMF) for 1 h, in order to cleave the Fmoc group. The resin was filtered once more, washed with DMF (5 times) and DCM and further dried *in vacuo*. To cleave the peptide and remove all Boc groups the resin was swollen in DCM and a mixture of TFA/TIS/water (1 mL; 95/2.5/2.5) was added. After 2 h of stirring it was filtered, the resin was washed with TFA (0.5 mL) and the filtrate, containing the cleaved peptide, was dried *in vacuo*. The crude product was purified by size exclusion chromatography (LH-20, DCM/MeOH = 1/1) to obtain product **7** (15 mg; 68 %) as colorless oil. ^{1}H-NMR (600 MHz; MeOD): δ

5.20 (ddd, J = 13.3, 6.8, 2.7 Hz, 1 H, CHCO$_2$), 4.44 (dd, J = 11.8, 3.1 Hz, 1 H, CH_aCO$_2$), 4.39 (t, J = 5.4 Hz, 1 H, CH(α) of serine), 4.33-4.23 (m, 5 H, CH(α) of lysine), 4.13 (dd, J = 11.8, 6.9 Hz, 1 H, CH_bCO$_2$), 3.95 (dd, J = 11.0, 4.9 Hz, 1 H, CH_aOH), 3.81 (dd, J = 11.0, 5.9 Hz, 1 H, CH_bOH), 3.64-3.61 (m, 1 H, CHNH$_2$), 3.32-3.29 (m, 2 H, CH_2N$_3$), 3.01-3.91 (m, 9 H, 4 × CH_2NH$_2$/SCH_aCH$_2$NH$_2$), 2.86-2.75 (m, 3 H, OCHCH_2S/SCH_aCH$_2$NH$_2$), 2.37-2.30 (m, 4 H, COCH_2), 1.96-1.23 (m, 82 H, CH$_2$ of fatty acids and lysines), 0.90 (t, J = 6.4 Hz, 6 H, 2 × CH$_3$) ppm; ^{13}C-NMR (selected signals from HSQC; MeOD): δ 71.1 (CHCO$_2$), 64.2 (CH_2CO$_2$), 62.3 (CH$_2$OH), 56.3 (CH(α) of Serine), 54.9-53.6 (5 × CH(α) of lysines and CH$_2$NH$_2$), 51.6 (CH_2N$_3$), 39.8 (4 × CH$_2$NH$_2$), 37.6 (SCH_2CH$_2$NH$_2$), 34.1 (2 × C(=O)CH_2), 32.9 (OCHCH_2S), 32.4-22.5 (CH$_2$ of lysines and fatty acids), 13.8 (2 × CH$_3$); HRMS (ESI-TOF): m/z [M+H]$^+$ calcd. for $C_{171}H_{138}N_{15}O_{12}S$: 1425.0365; found 1425.0384

2.2. Synthesis of Monofunctionalized linker

2,5-Dioxopyrrolidin-1-yl 3-{4,5-dibromo-3,6-dioxo-2-[5-(1H-1,2,3-triazol-4-methyl-4-yl) pentanoic acid]-2,3-dihydropyridazin-1(6H)-yl) propanoate (16)

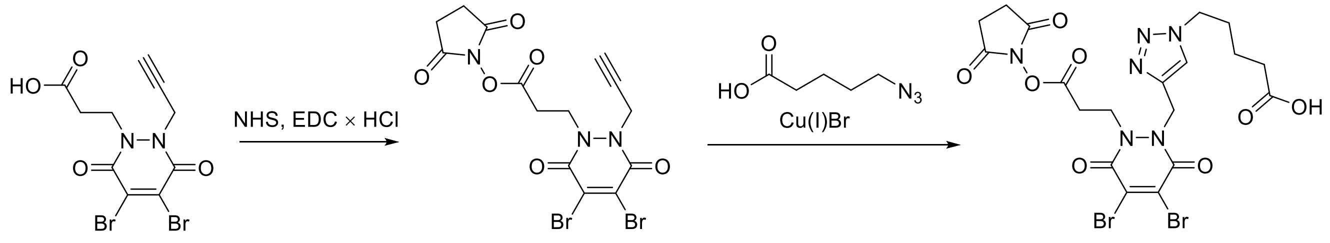

14 **15** **16**

Compound **14**[3] (100 mg; 0.26 mmol) was dissolved in dry DCM (5 mL) under Argon followed by the addition of NHS (45 mg; 0.40 mmol) and EDC × HCl (75 mg; 0.40 mmol). The reaction mixture was stirred for 16 h and was then washed with aqu. satd. NH$_4$Cl and twice with water. The organic phase was dried over Na$_2$SO$_4$, the solvent was removed *in vacuo* and the crude product **15** (114 mg containing 10 w % NHS) was used in the next step without further purification.

The crude product **15** (114 mg; containing approx. 103 mg of desired intermediate) and 5-azido valeric acid (40 mg; 0.28 mmol) were dissolved in degassed dry DMF (2 mL) under Argon followed by the addition of CuBr (4 mg; 0.03 mmol). It was stirred at 22 °C for 16 h until all **15** was consumed and the solvent was then removed *in vacuo*. The crude product was purified by silica gel chromatography (DCM/MeOH/AcOH = 100/0/0.1 → 100/5/0.1) and product **16** (111 mg; 68 % over two steps) was obtained as a colorless amorphous solid. R$_f$: 0.42 (DCM/MeOH/AcOH = 100/5/0.1); ^{1}H NMR (600 MHz, (CD$_3$)$_2$CO): δ = 8.05 (s, 1 H, COOH), 8.03 (s, 1 H, CH of triazole), 5.49 (s, 2 H, N-CH_2-triazole), 4.69 (t, J = 7.2 Hz, 2 H, NCH_2CH$_2$COOR), 4.45 (t, J = 7.1 Hz, 2 H, NCH_2CH$_2$CH$_2$), 3.25 (t, J = 7.2, 2 H, NCH$_2$$CH_2$COOR),

2.90 (s, 4 H, CH_2 of pyrrolidine), 2.35 (t, *J* = 3.4 Hz, 2 H, HOOCCH_2), 2.0-1.93 (m, 2 H, $NCH_2CH_2CH_2$), 1.64-1.58 (m, 2 H, $NCH_2CH_2CH_2$) ppm; ^{13}C NMR (150 MHz, $(CD_3)_2CO$): 174.2, 170.2, 167.4, 154.7, 154.0, 141.9, 136.7, 136.6 (quaternary carbons), 124.6 (CH of triazole), 50.5 ($NCH_2CH_2CH_2$), 43.8 (N-CH_2-triazole), 43.6 (NCH_2CH_2COOR), 33.3 (HOOCCH_2), 29.6 ($NCH_2CH_2CH_2$), 28.3 (NCH_2CH_2COOR), 26.3 (CH_2 of pyrrolidine), 22.5 ($NCH_2CH_2CH_2$) ppm; HRMS (ESI-TOF): m/z $[M+H]^+$ calcd. for $C_{19}H_{21}Br_2N_6O_8$: 618.9782; found 618.9771.

5-(4-((2-(1-((1R,8S,9s)-bicyclo[6.1.0]non-4-yn-9-yl)-3,14-dioxo-2,7,10-trioxa-4,13-diazahexadecan-16-yl)-4,5-dibromo-3,6-dioxo-3,6-dihydropyridazin-1(2H)-yl)methyl)-1H-1,2,3-triazol-1-yl)pentanoic acid

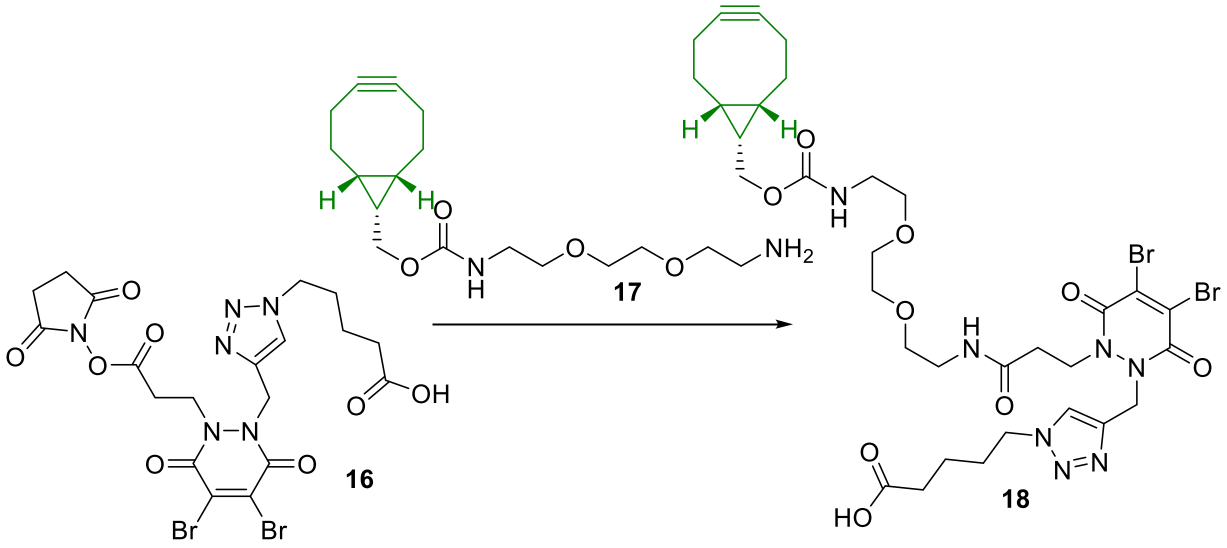

16 (70 mg; 113 µmol) was dissolved in MeCN (1 mL) under Argon before commercially available BCN-amine **17** (40 mg; 124 µmol) dissolved in dry DCM (2 mL) was added dropwise. After complete addition it was stirred at 22 °C for 18 h and then, the solvent was removed *in vacuo*. The crude product was purified with silica gel chromatography (DCM/MeOH/AcOH = 100/0/0.5 → 100/10/0.5) which afforded **18** (77 mg; 82 %), a colorless oil, as a mixture of rotamers. R_f : 0.11 (DCM/MeOH/AcOH = 100/5/1); ^{1}H NMR (600 MHz, $CDCl_3$): δ = 7.75 (s, 1 H, CH of triazole), 7.52 (bs, 0.09 H, NH), 6.75 (bs, 0.38 H, NH), 6.07 (bs, 0.37 H, NH), 5.46 (s, 2 H, N-CH_2-triazole), 5.34 (bs, 0.45 H, NH), 4.64 (bs, 2 H, NCH_2CH_2COOR), 4.36 (t, *J* = 6.4 Hz, 2 H, $NCH_2CH_2CH_2$), 4.22-4.11 (m, 2 H, CH_2OCONHR), 3.66-3.52 (m, 8 H, $CH_2O(CH_2)_2OCH_2$), 3.45-3.32 (m, 4 H, 2 × OCH_2CH_2N), 2.63 (bt, *J* = 7.4 Hz, 2 H, NH(C=O)CH_2), 2.36 (t, *J* = 6.9 Hz, 2 H, HCOOCH_2), 2.33-2.16 (m, 4 H, $CH_2C{\equiv}CCH_2$), 2.00-1.92 (m, 2 H, $HCOOCH_2CH_2CH_2$), 1.66-1.52 (m, 6 H, $CH_2CH_2C{\equiv}CCH_2CH_2$/$HCOOCH_2CH_2$), 1.41-1.30 (m, 1 H, CH of cyclopropyl), 1.0-0.89 (m, 2 H, 2 × CH of cyclopropyl) ppm; ^{13}C NMR (150 MHz, $CDCl_3$): δ, 169.8, 156.9, 153.4, 153.0, 141.0, 136.3, 135.5 (quaternary carbons), 124.1 (CH of triazole), 98.8 ($CH_2C{\equiv}CCH_2$), 70.0-69.5 (4 C,

*CH₂*O(*CH₂)₂OCH₂*), 62.8 (*CH₂*OCONHR), 50.1 (N*CH₂*CH₂CH₂), 45.2 (N*CH₂*CH₂COOR), 42.9 (N-*CH₂*-triazole), 40.6, 39.3 (NH*CH₂*CH₂O), 33.9 (NCH₂*CH₂*COOR), 32.8 (HOOC*CH₂*), 29.2, 29.0 (3 C, NCH₂*CH₂*CH₂, *CH₂*C≡C*CH₂*), 21.5 (NCH₂CH₂*CH₂*), 21.3 (*CH₂*CH₂C≡CCH₂*CH₂*), 20.7, 20.1, 17.7 (CH of cyclopropyl) ppm; HRMS (ESI-TOF): m/z $[M+NH_4]^+$ calcd. for $C_{32}H_{47}N_8O_9Br_2$: 847.1807; found 847.1528.

2,5-dioxopyrrolidin-1-yl 5-(4-((2-(1-((1R,8S,9s)-bicyclo[6.1.0]non-4-yn-9-yl)-3,14-dioxo-2,7,10-trioxa-4,13-diazahexadecan-16-yl)-4,5-dibromo-3,6-dioxo-3,6-dihydropyridazin-1(2H)-yl)methyl)-1H-1,2,3-triazol-1-yl)pentanoate (19)

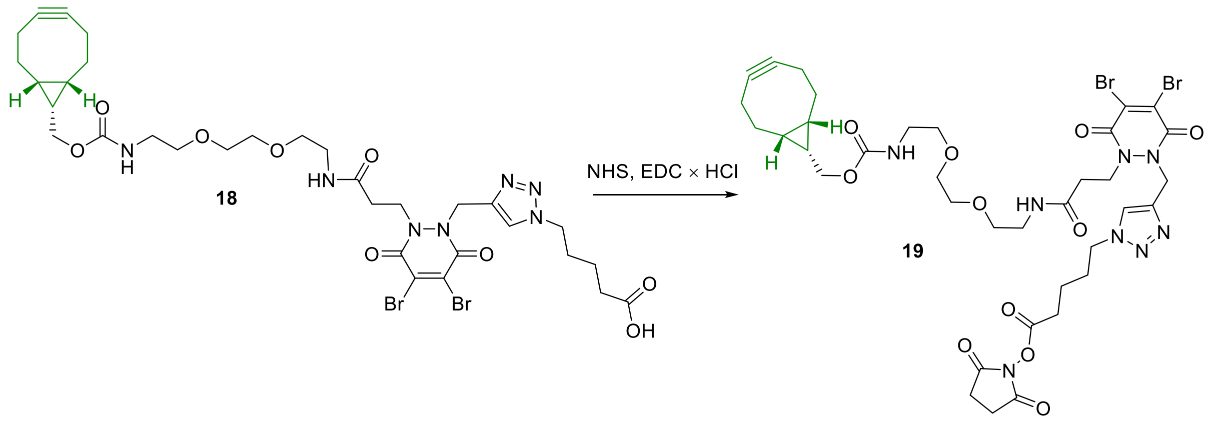

Compound **18** (20 mg; 24 µmol) was dissolved in dry DCM (1.5 mL) under Argon followed by the addition of NHS (5.5 mg; 48 µmol) and EDC × HCl (9.2 mg; 48 µmol). The reaction mixture was stirred for 2 h, before it was diluted with EtOAc and then washed with aqu. satd. NH_4Cl and twice with water. The organic phase was dried over Na_2SO_4, the solvent was removed *in vacuo* and the crude product was purified with silica gel chromatography (DCM/MeOH = 20/0 → 20/1) to obtain product **19** (13 mg; 58 %) as colorless oil. R_f : 0.33 (DCM/MeOH = 20/1); ^1H NMR (600 MHz, $CDCl_3$): δ = 7.75 (s, 1 H, CH of triazole), 7.64 (bs, 0.24 H, NH), 6.48 (bs, 0.78 H, NH), 5.50 (s, 2 H, N-*CH₂*-triazole), 5.26 (bs, 0.72 H, NH), 4.64 (t, *J* = 6.6 Hz, 2 H, N*CH₂*CH₂COOR), 4.37 (t, *J* = 7.1 Hz, 2 H, N*CH₂*CH₂CH₂), 4.20-4.11 (m, 2 H, *CH₂*OCONHR), 3.67-3.51 (m, 8 H, *CH₂*O(*CH₂)₂OCH₂*), 3.45-3.32 (m, 4 H, 2 × OCH₂*CH₂*N), 2.84 (bs, 4 H, *CH₂* of pyrrolidine), 2.69 (bt, *J* = 6.9 Hz, 2 H, NH(C=O)*CH₂*), 2.64 (t, *J* = 7.9 Hz, 2 H, pyrrolidine-OOC*CH₂*), 2.31-2.17 (m, 4 H, *CH₂*C≡C*CH₂*), 2.08-2.00 (m, 2 H, pyrrolidine-OOCCH₂CH₂*CH₂*), 1.81-1.52 (m, 6 H, *CH₂*CH₂C≡CCH₂*CH₂*/HCOOCH₂*CH₂*), 1.41-0.91 (m, 3 H, 3 × CH of cyclopropyl) ppm; ^{13}C NMR (150 MHz, $CDCl_3$): δ, 169.1, 167.9, 153.3, 153.0, 141.2, 136.7, 136.3 (quaternary carbons), 123.9 (CH of triazole), 98.9 (*CH₂*C≡C*CH₂*), 70.3-69.7 (4 C, *CH₂*O(*CH₂)₂OCH₂*), 62.8 (*CH₂*OCONHR), 49.8 (N*CH₂*CH₂CH₂), 45.6 (N*CH₂*CH₂COOR), 42.8 (N-*CH₂*-triazole), 40.8, 39.5 (NH*CH₂*CH₂O), 34.1 (NCH₂*CH₂*COOR), 30.4 (HOOC*CH₂*), 29.1, 28.8 (3 C, NCH₂*CH₂*CH₂, *CH₂*C≡C*CH₂*), 25.7 (*CH₂* of pyrrolidine), 21.6 (NCH₂CH₂*CH₂*), 21.4 (*CH₂*CH₂C≡CCH₂*CH₂*), 20.2, 17.8 (3 C, CH of cyclopropyl) ppm; HRMS (ESI-TOF): m/z $[M+H]^+$ calcd. for $C_{36}H_{46}Br_2N_8O_{11}$: 925.1726; found 925.1738.

((1R,8S,9s)-bicyclo[6.1.0]non-4-yn-9-yl)methyl (2-(2-(2-(3-(2-((1-(5-((4-((4-amino-2-butyl-1H-imidazo[4,5-c]quinolin-1-yl)methyl)benzyl)amino)-5-oxopentyl)-1H-1,2,3-triazol-4-yl)methyl)-4,5-dibromo-3,6-dioxo-3,6-dihydropyridazin-1(2H)-yl)propanamido)ethoxy)ethoxy)ethyl)carbamate

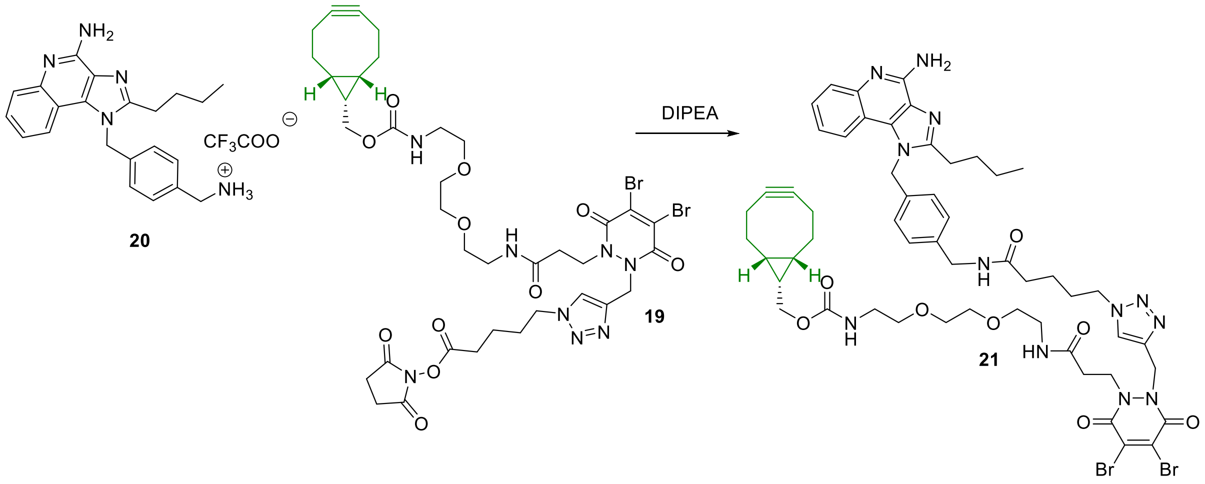

Imidazocholin **20** (2.3 mg; 4.9 µmol) was dissolved in dry DMF (0.2 mL) under Argon, followed by the addition of DIPEA (1.0 µL; 5.8 µmol). It was stirred at 22 °C for 5 min and then **19** (3.6 mg; 3.9 µmol) dissolved in dry DMF (0.4 mL) was added dropwise. Stirring was continued and after full consumption of substrate **19** (16 h) the solvent was removed *in vacuo*. The crude product was purified by HPLC (C18 column; water/MeCN/AcOH = 80/20/0.1 → 20/80/0.1) to obtain compound **21** (1.5 mg; 33 %) as colorless oil. ^{1}H NMR (600 MHz, $CDCl_3$): δ = 7.85 (d, *J* = 8.8 Hz, 1 H, Ar), 7.78 (d, *J* = 8.4 Hz, 1 H, Ar), 7.64 (s, 1 H, CH of triazole), 7.45 (t, *J* = 7.87, 1 H, Ar), 7.25-7.14 (m, 7 H, Ar), 7.01 (d, *J* = 6.6 Hz, 2 H, Ar), 6.61 (bs, 1 H, NH), 5.88 (bs, 1 H, NH), 5.72 (s, 2 H, N-CH_2-Phenyl), 5.43 (s, 2 H, N-CH_2-triazole), 4.65 (t, *J* = 7.8 Hz, 2 H, N$CH_2$$CH_2$COOR), 4.39 (d, *J* = 7.8 Hz, 1 H, PhCH_2NHCO), 4.32 (t, *J* = 6.6 Hz, 2 H, N$CH_2$$CH_2$$CH_2$), 4.19-4.10 (m, 2 H, CH_2OCONHR), 3.66-3.49 (m, 8 H, $CH_2O(CH_2)_2OCH_2$), 3.45-3.32 (m, 4 H, 2 × O$CH_2$$CH_2$N), 2.86 (t, *J* = 10.3 Hz, 2 H, $CH_2(CH_2)_2CH_3$), 2.67 (bt, *J* = 6.9 Hz, 2 H, NH(C=O)CH_2), 2.31-2.17 (m, 6 H, NHCO$CH_2(CH_2)_3$/CH_2CH≡CHCH_2), 2.97-1.84 (m, 2 H, NHCO$CH_2CH_2CH_2$), 1.84-1.77 (m, 2 H, $CH_3CH_2CH_2$), 1.71-1.52 (m, 4 H, CH_2CH_2CH≡CHCH_2CH_2), 1.47-1.40 (m, 2 H, CH_2CH_3), 1.33-1.18 (m, 3 H, 3 × CH of cyclopropyl), 0.93 (t, *J* = 7.6 Hz, 1 H, CH_3) ppm; ^{13}C NMR (150 MHz, $CDCl_3$): δ 177.9, 171.8, 169.1 (*C*=ONH), 154.9 (*C*$(CH_2)_3CH_3$), 153.0 (2 × *C*OCBr), 138.5, 137.9, 134.0, 128.8, 128.0, 125.4, 123.9 (Ar), 123.6 (CH of triazole), 122.8, 120.0, 114.2 (Ar), 98.9 (CH_2C≡CCH_2), 70.3-69.7 (4 C, $CH_2O(CH_2)_2OCH_2$), 62.7 (CH_2OCONHR), 50.2 (N$CH_2CH_2CH_2$), 48.8 (NCH_2Ph), 45.1 (NCH_2CH_2COOR), 43.0 (PhCH_2NHCO), 42.5 (N-CH_2-triazole), 40.5, 39.4 (NHCH_2CH_2O), 35.5 (CO$CH_2(CH_2)_3$), 33.6 (NCH_2CH_2COOR), 30.2 , 29.8, 29.7, 29.2, (4 C, CH_2CH_2-triazole, N$CH_2CH_2CH_2$, CH_2C≡CCH_2), 27.1 (NC$CH_2(CH_2)_2CH_3$) 22.9-17.8 (residual aliphatic C), 13.6 (CH_3) ppm; HRMS (ESI-TOF): m/z [M+Na]$^+$ calcd. for $C_{54}H_{66}Br_2N_{12}NaO_8$: 1191.3386; found 1191.3364

2.3. Synthesis of bifunctionalized linker

Strain-promoted click reaction

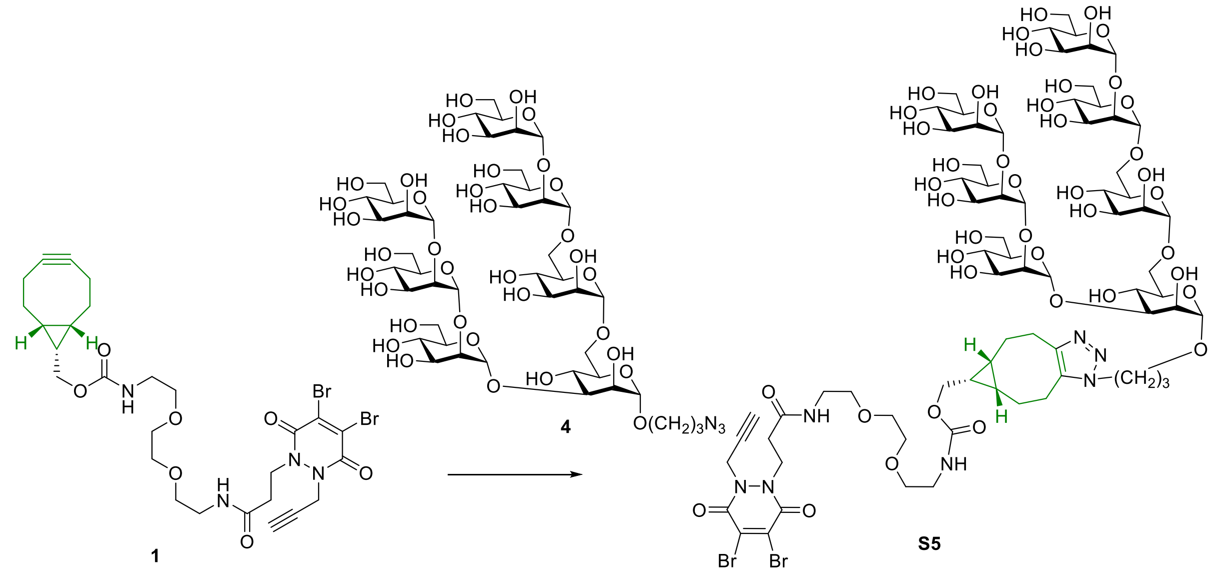

1 (1.2 mg; 1.7 µmol) and **4**[4] (2.1 mg; 1.7 µmol) were dissolved in a mixture of MeCN/H_2O (0.6 mL; 2/1) and it was stirred at 22 °C for 16 h until HPLC analysis revealed full consumption of the starting materials. The reaction mixture was then directly submitted to HPLC chromatography (C8 column; MeCN/H_2O = 5/95 → 100/0) to yield product **S5** (1.5 mg; 45 %) as colorless oil; ^{1}H NMR (600 MHz, D_2O): δ = 5.34, 5.30, 5.13, 5.03, 5.02 (5 × s, 5 H, anomeric H), 4.99 (s, 2 H, NCH_2C≡CH), 4.84, 4.74 (2 × s, 2 H, anomeric H), 4.54 (t, J = 7.7 Hz, 2 H, N$CH_2$$CH_2$C=O), 4.42 (t, J = 6.8 Hz, 2 H, CH_2-triazole), 4.21-3.58 (m, 51 H), 3.54 (t, J = 5.5 Hz, 2 H), 3.45-3.39 (m, 1 H, triazole-$CH_2CH_2CH_a$), 3.34-3.29 (m 5 H), 3.06-2.97 (m, 2 H), 2.87-2.75 (m, 2 H), 2.67 (t, J = 7.7 Hz, 2 H, NCH_2CH_2C=O), 2.28-2.09 (m, 5 H), 1.65-1.52 (m, 3 H), 1.26-1.17 (m, 1 H), 1.07-0.97 (m, 2 H); ^{13}C NMR (150 MHz, D_2O, selected signals from HSQC): δ 102.2 (2 C), 100.8, 100.7, 100.1, 99.6, 98.0 (anomeric C), 79.0 (C-3^A from glycans), 78.8-78.3 (3 × C, C-2 from glycan); 61.4-60.5 (7 × CH_2 from glycan), 45.2 (CH_2-triazole), 44.8 (N$CH_2$$CH_2$C=O), 38.1 (N$CH_2$C≡CH), 33.8 (N$CH_2CH_2$C=O) ppm; HRMS (ESI-TOF): m/z $[M+2H]^{2+}$ calcd. for $C_{72}H_{113}Br_2N_7O_{43}$: 960.7613; found 960.7649.

Copper-Click reaction

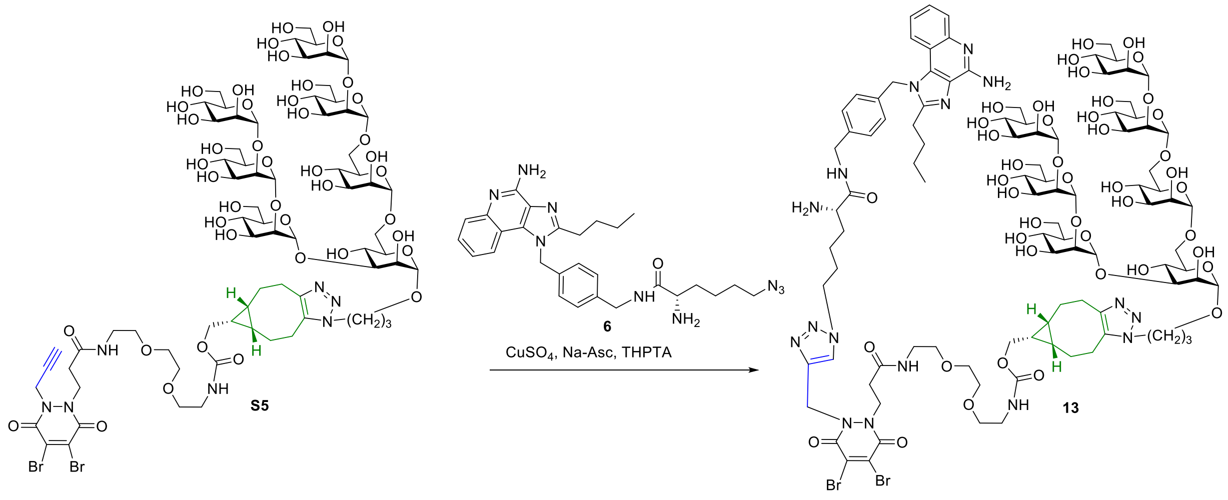

S5 (0.5 mg; 0.26 µmol) was dissolved in H_2O (150 µL) followed by the addition of **6** (0.27 mg; 0.52 µmol) dissolved in DMF (100 µL). Subsequently, a premixed solution of $CuSO_4$ and THPTA (12.5 µL; 20 mM of THPTA and 5 mM of $CuSO_4$) was added and the click reaction was then initiated by the addition of sodium ascorbate (26 µL; 100 mM in H_2O). It was stirred at 22 °C for 3 h and the reaction mixture was then subjected to HPLC purification (C18 column; MeCN/H_2O/AcOH = 5/95/0.1 → 70/30/0.1) and after lyophilization product **13** (0.4 mg; 63 %) was obtained as a colorless solid; HRMS (ESI-TOF): m/z $[M+2H]^{2+}$ calcd. for $C_{72}H_{113}Br_2N_7O_{43}$: 1217.4096; found 1217.4099.

3. Analyctical data of azide and linker compounds

^1H, ^{13}C, HSQC NMR-spectra and HPLC-ELSD (ZIC-HILIC-column) of compound **3**

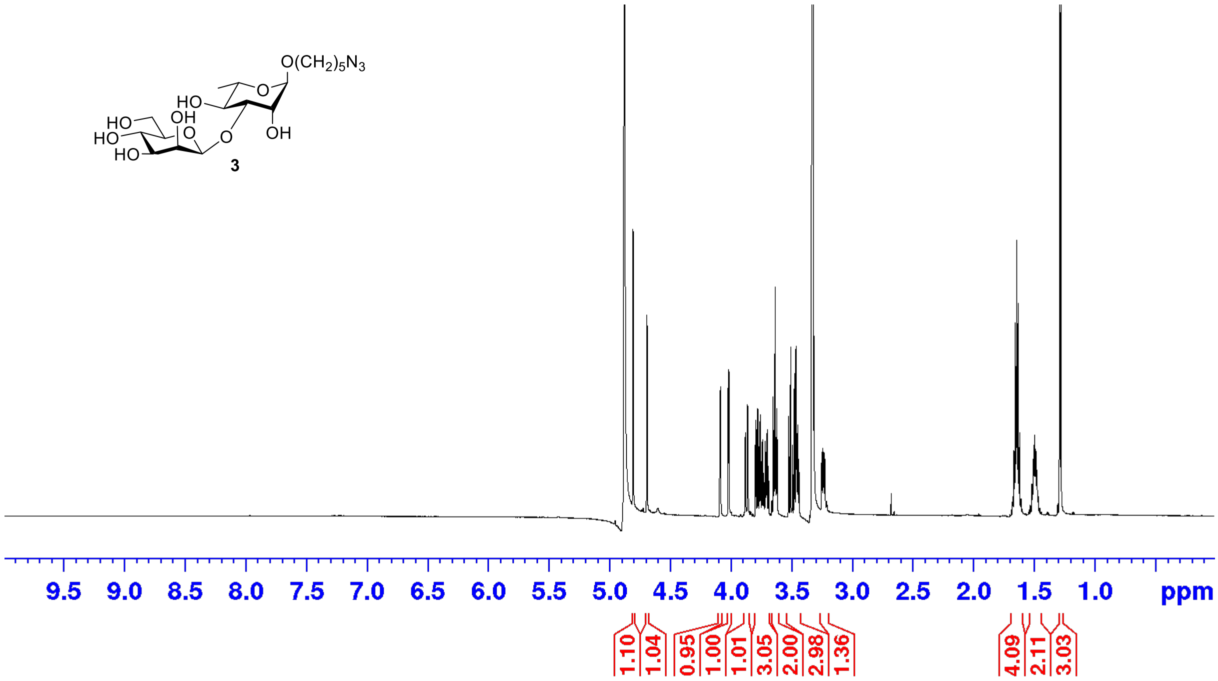

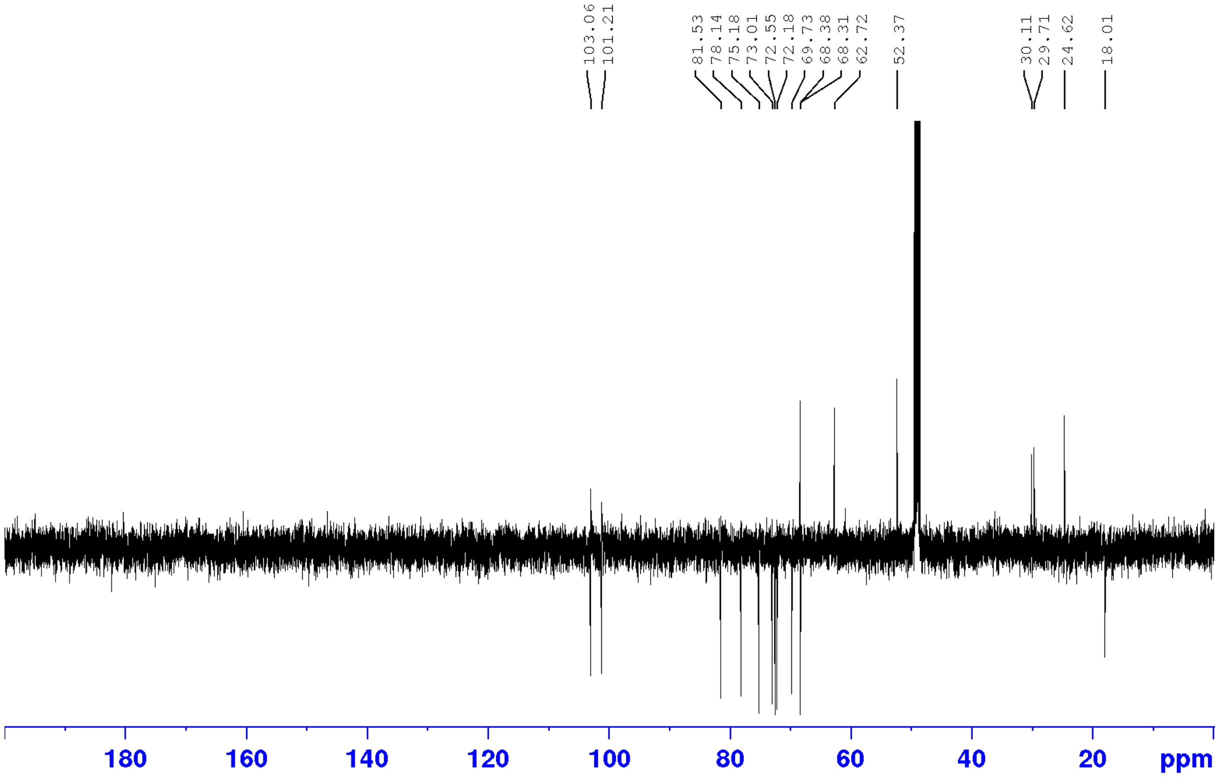

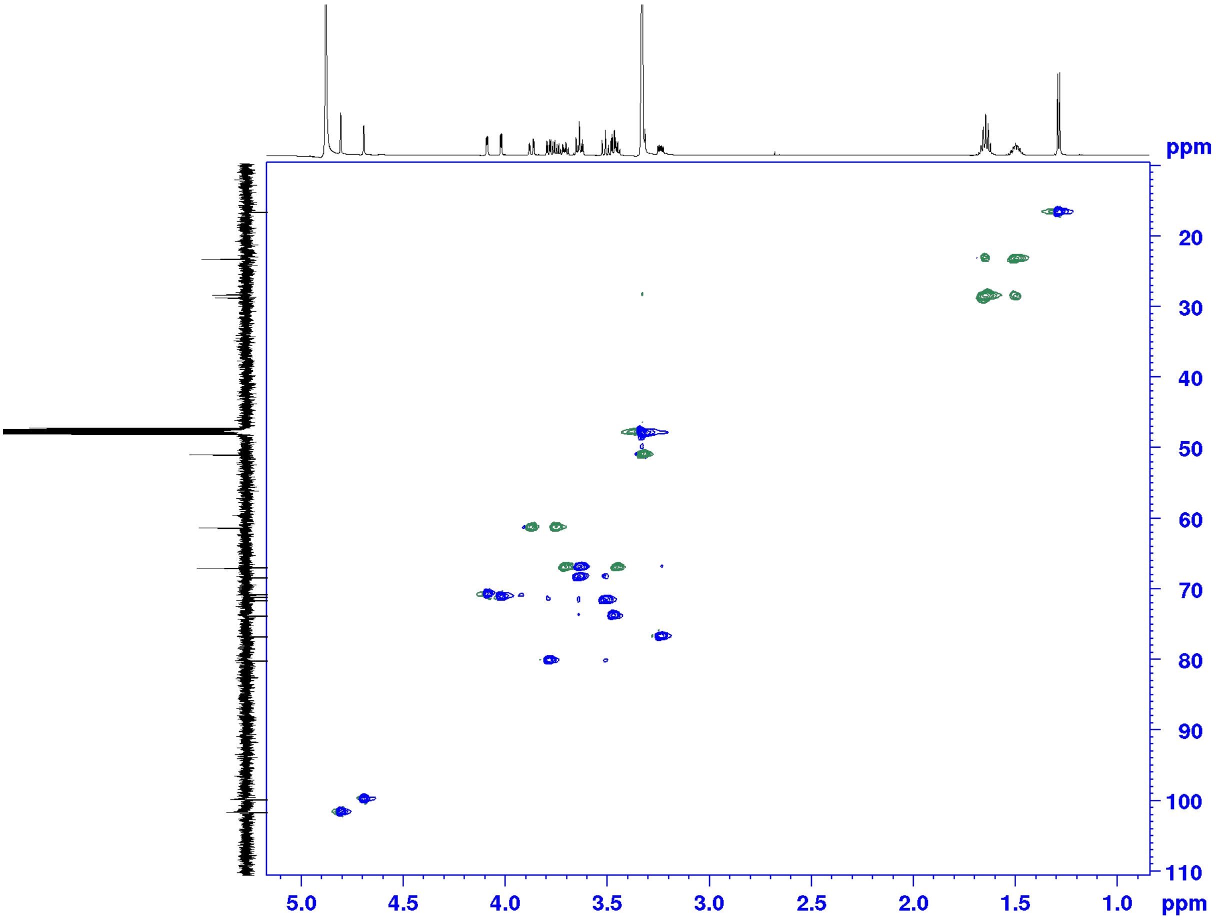
ppm
20
30
40
50
60
70
80
90
100
110
5.0
4.5
4.0
3.5
3.0
2.5
2.0
1.5
1.0
ppm

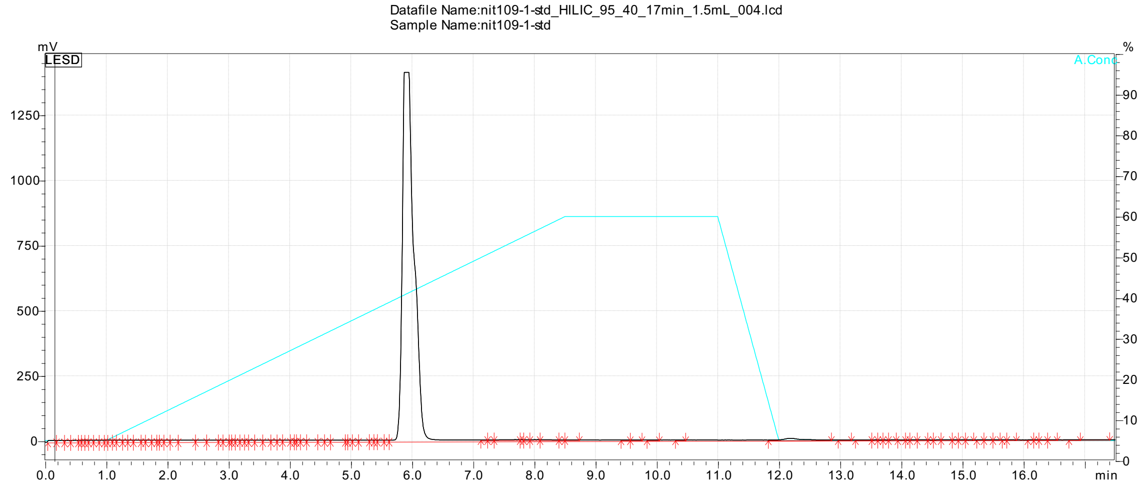
Datafile Name:nit109-1-std_HILIC_95_40_17min_1.5mL_004.lcd
Sample Name:nit109-1-std
mV
LESD
%
A.Conc
1250
1000
750
500
250
0
90
80
70
60
50
40
30
20
10
0
0.0
1.0
2.0
3.0
4.0
5.0
6.0
7.0
8.0
9.0
10.0
11.0
12.0
13.0
14.0
15.0
16.0
min

^{1}H and HSQC-NMR spectra of compound **5**

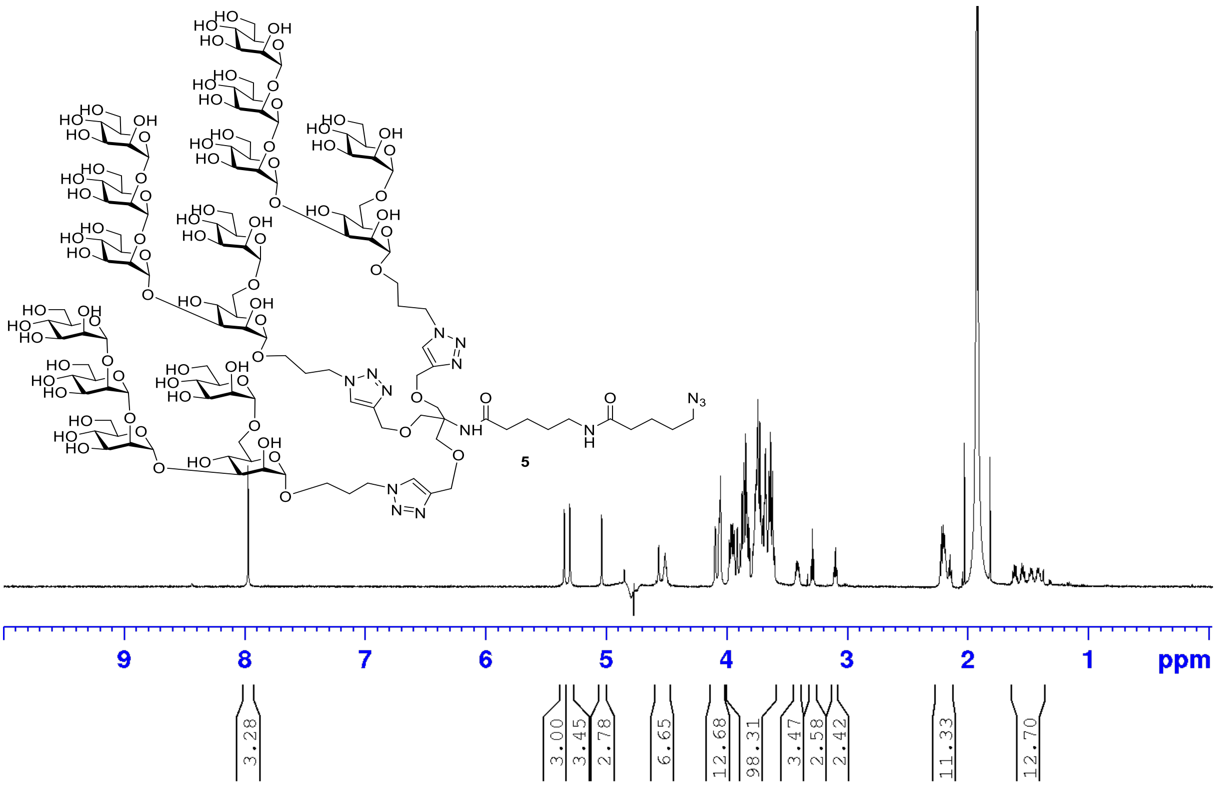

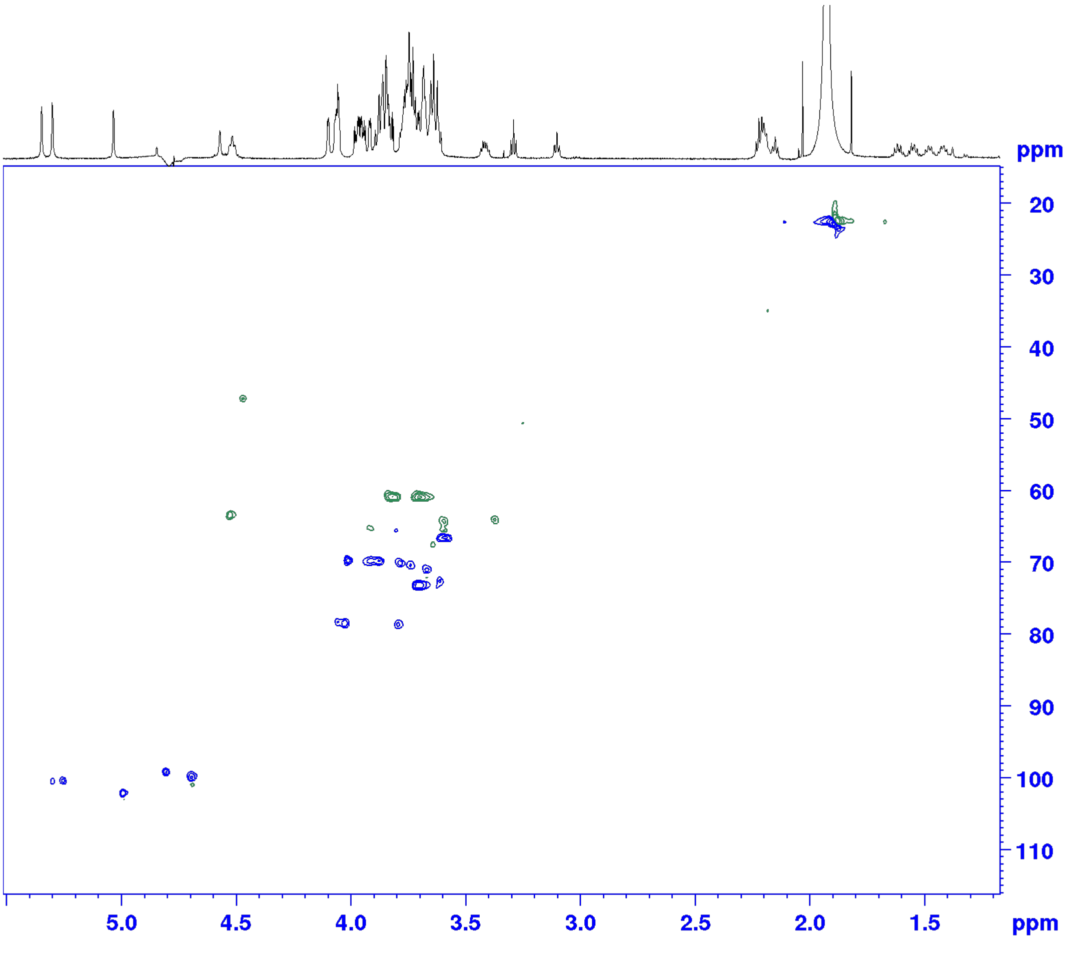

^{1}H and ^{13}C NMR-spectra of compound **S3**

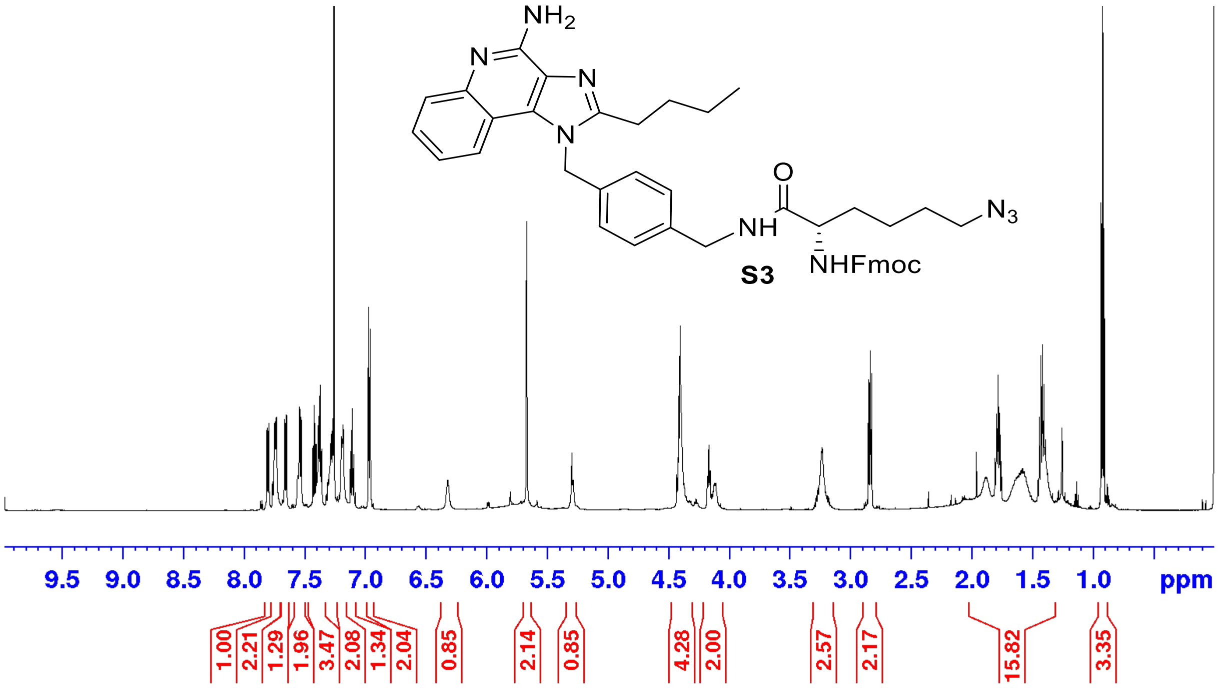

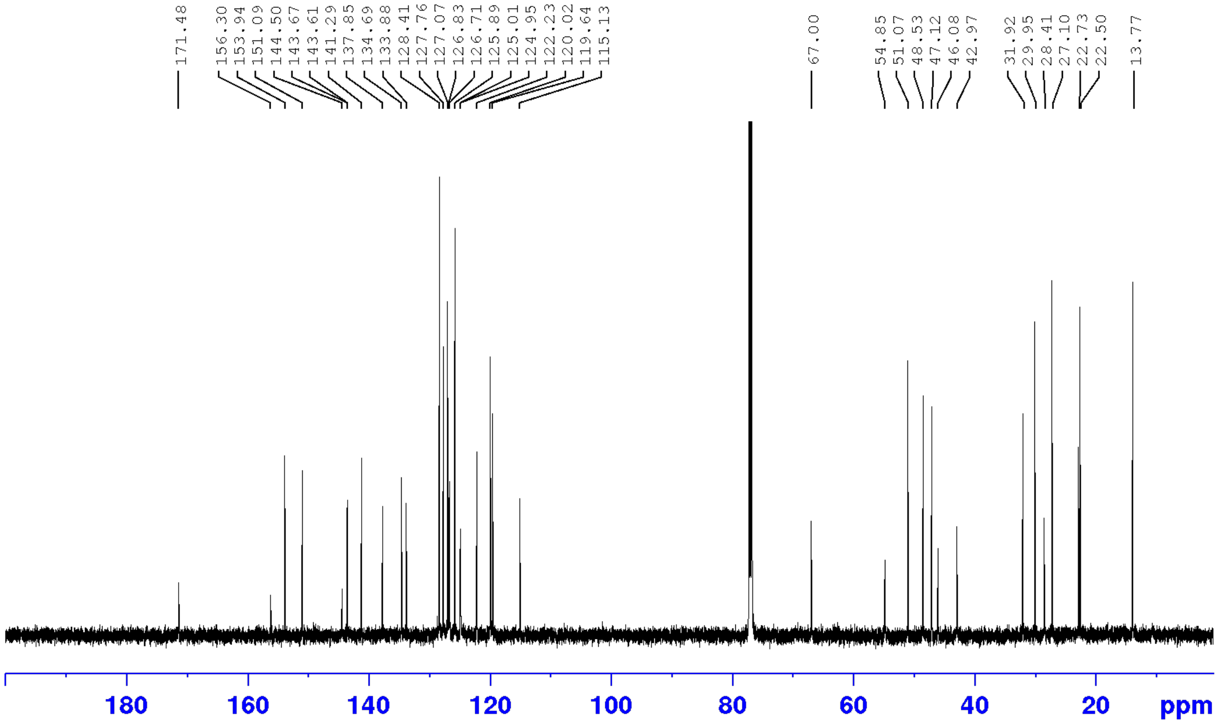

^{1}H, ^{13}C, HSQC-spectra and HPLC-ELSD (ZIC-HILIC-column) of compound **6**

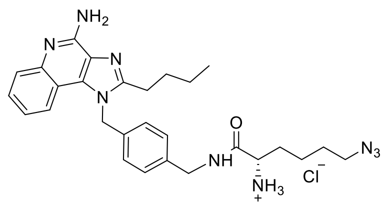

6

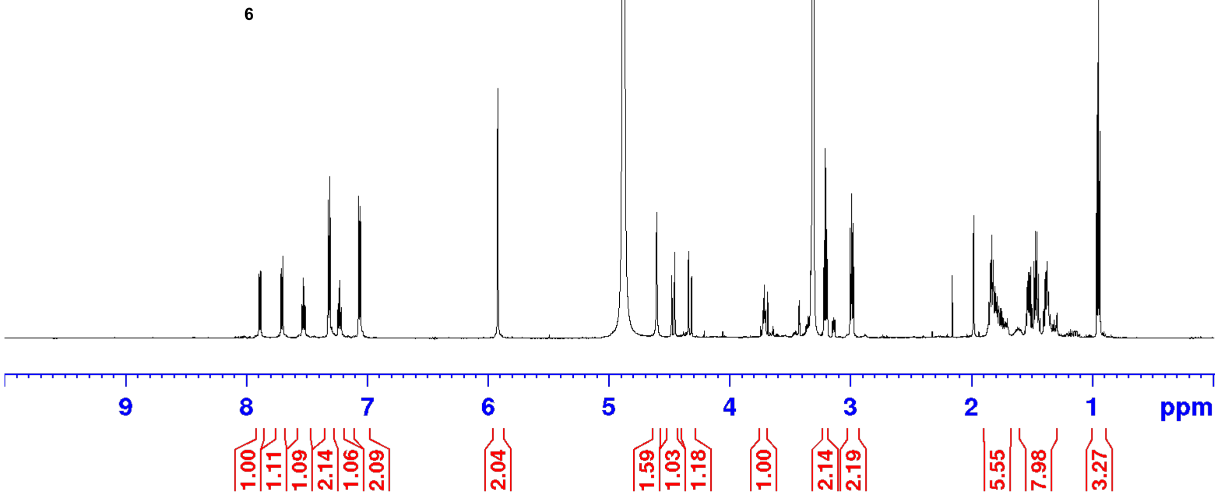

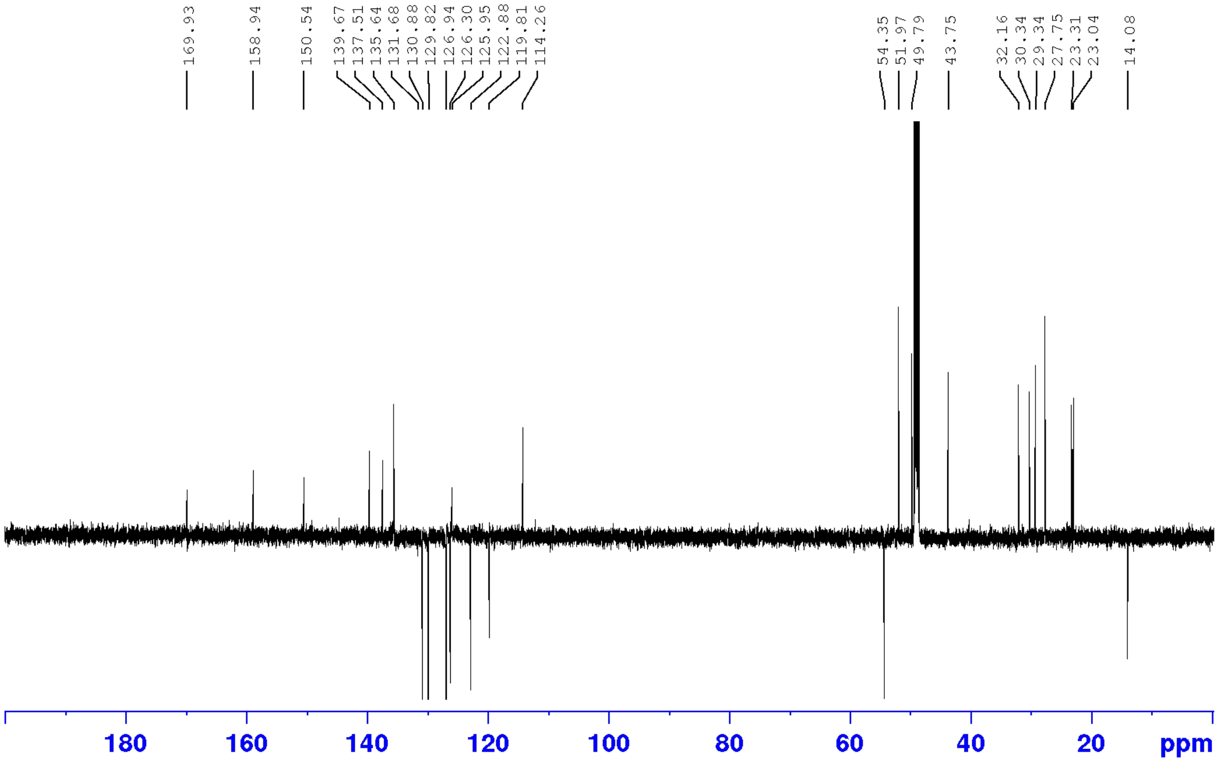

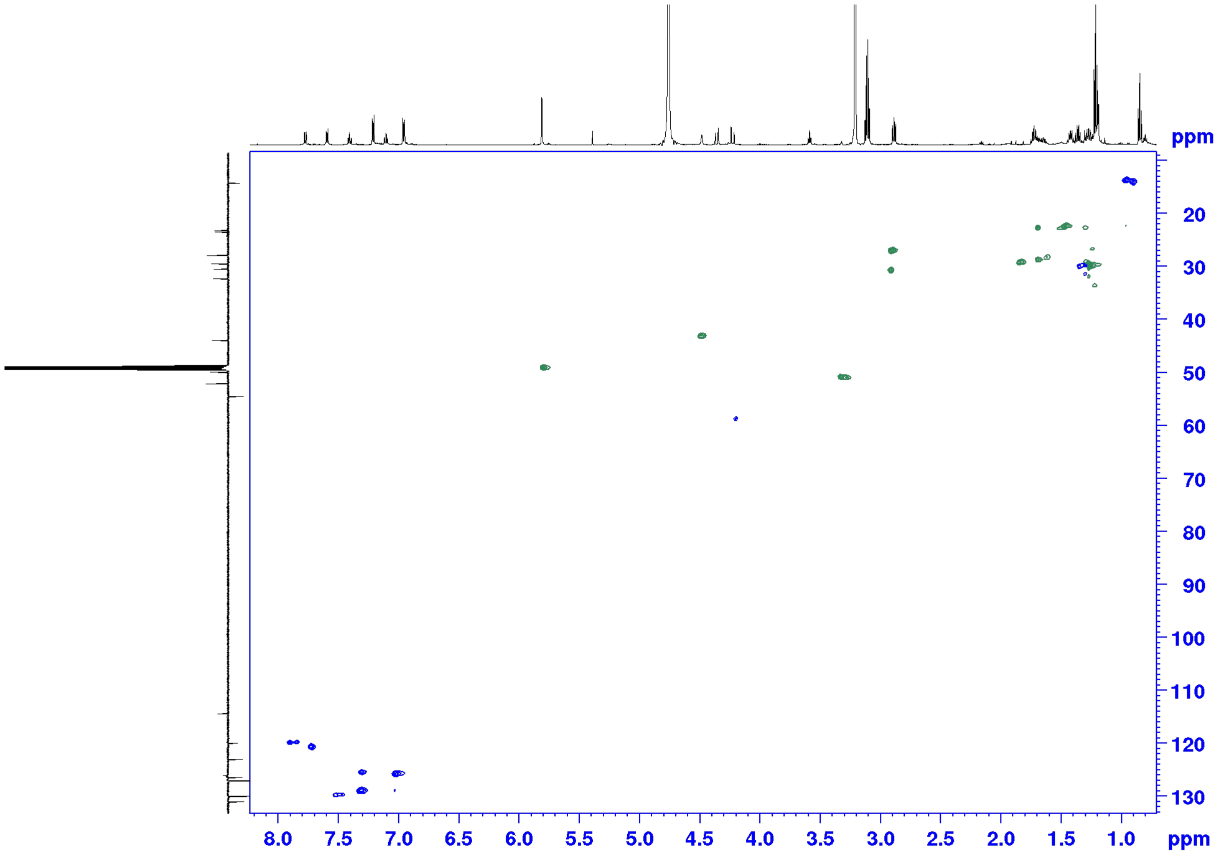
ppm
8.0
7.5
7.0
6.5
6.0
5.5
5.0
4.5
4.0
3.5
3.0
2.5
2.0
1.5
1.0
ppm
20
30
40
50
60
70
80
90
100
110
120
130

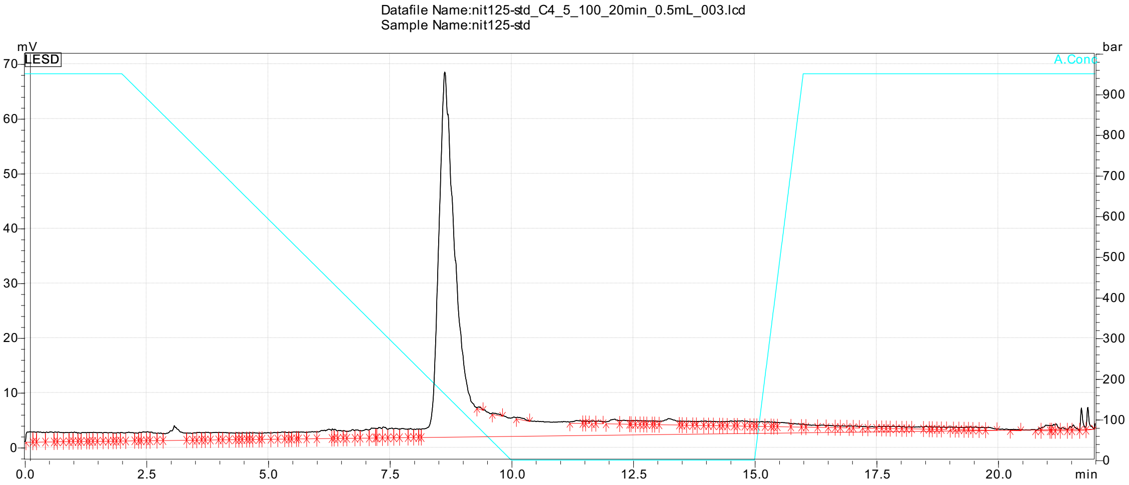
Datafile Name:nit125-std_C4_5_100_20min_0.5mL_003.lcd
Sample Name:nit125-std
mV
ELSD
A.Conc
bar
min

^{1}H, HSQC NMR-spectra and HPLC-ELSD (C4-column) of compound **7**

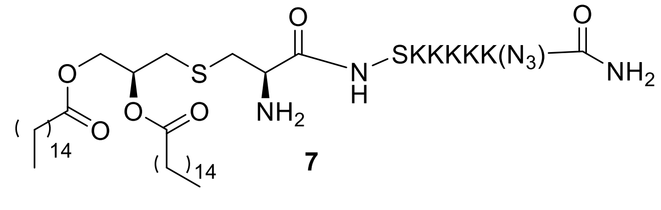

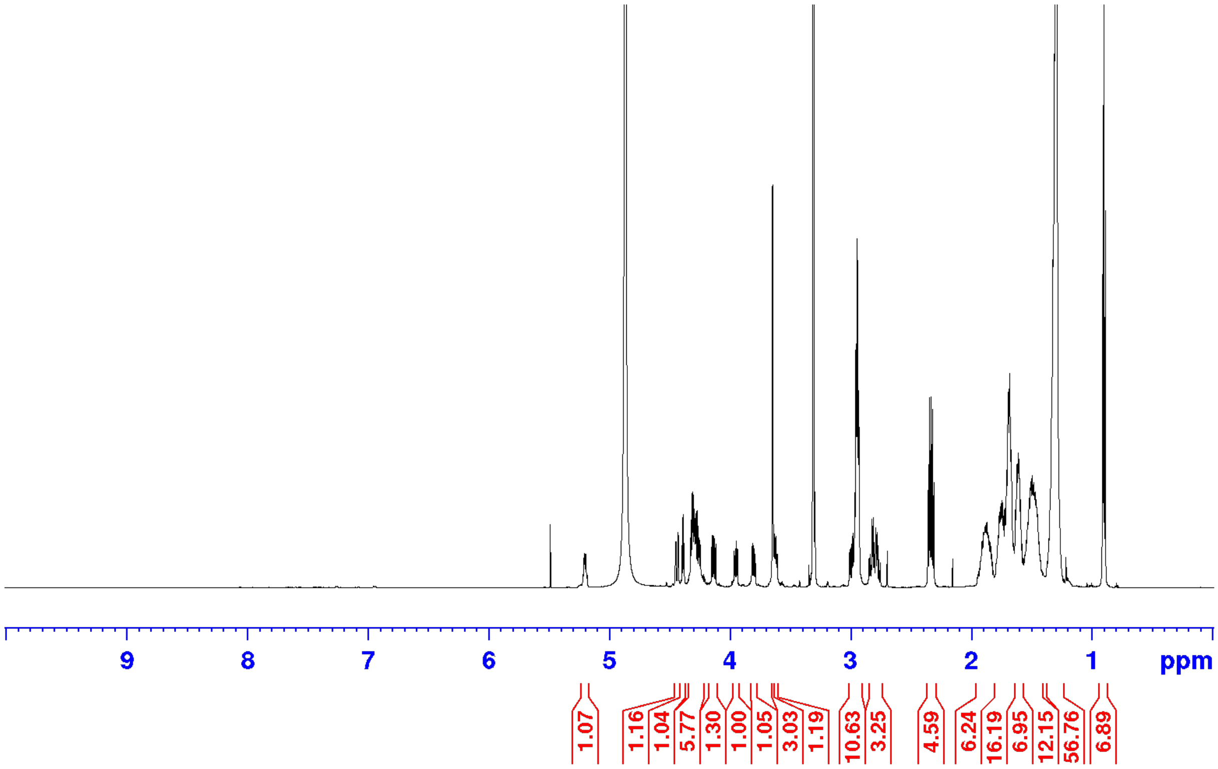

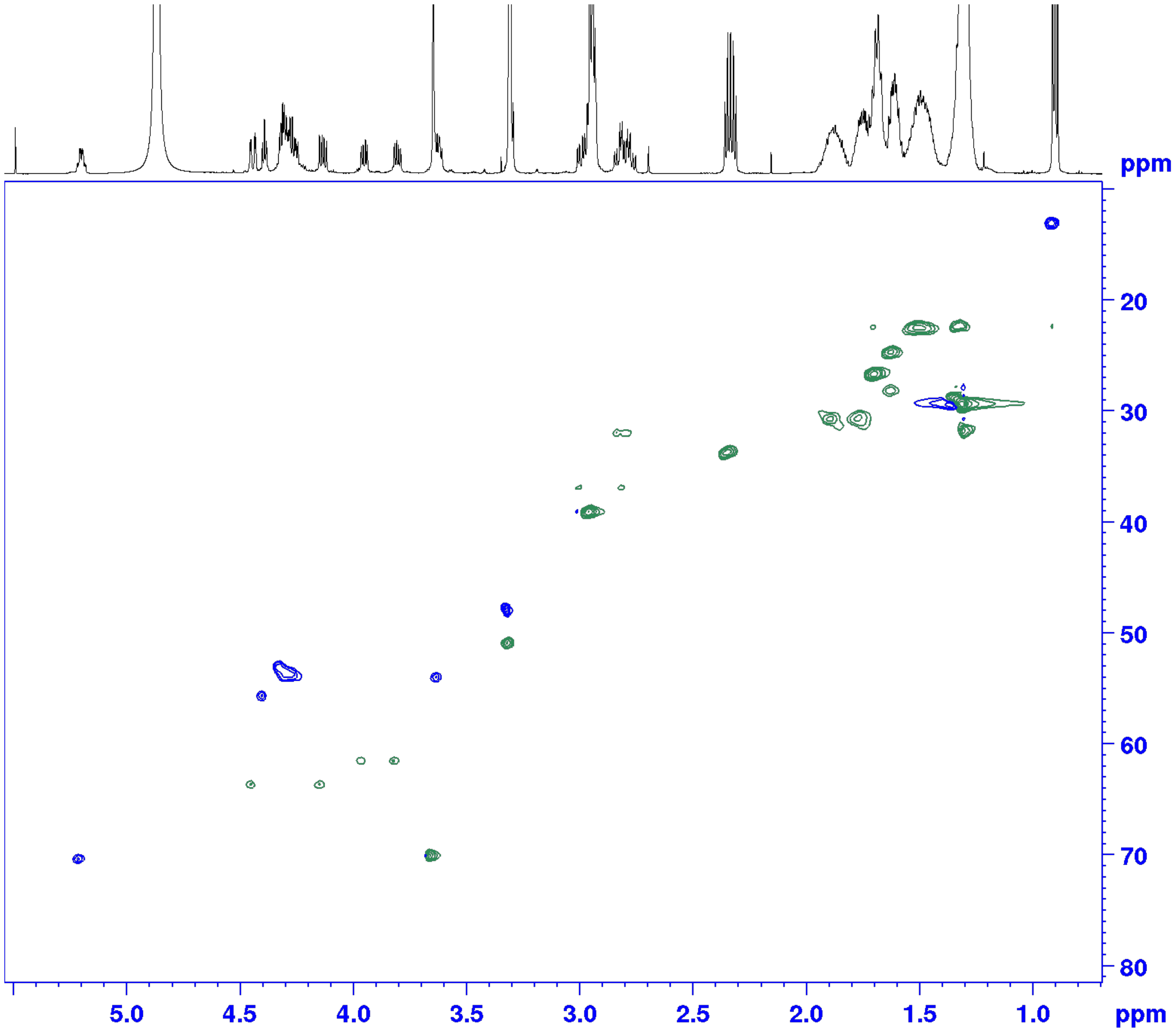

Datafile Name:nit148-DMSO_C4_5_100_20min_0.5mL_004.lcd
Sample Name:nit148-DMSO

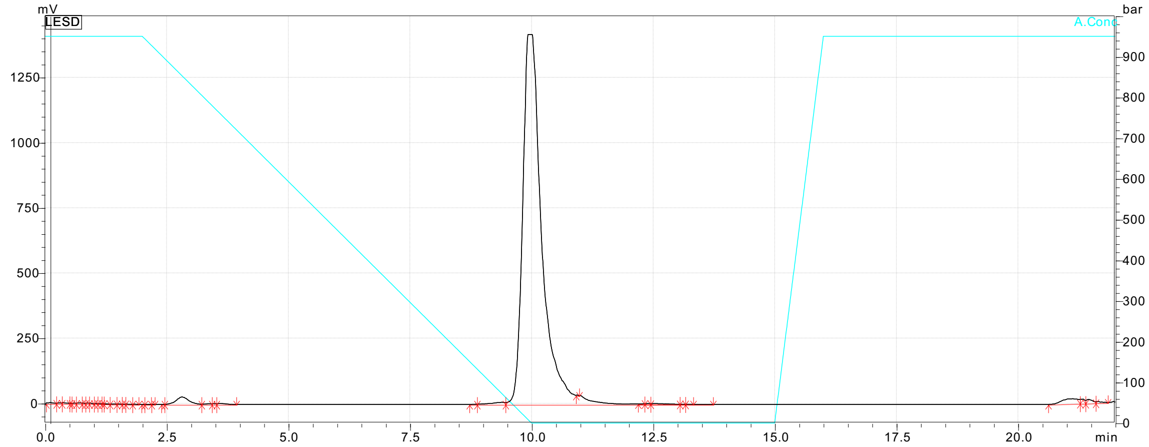

^1H and ^{13}C NMR-spectra of compound **16**

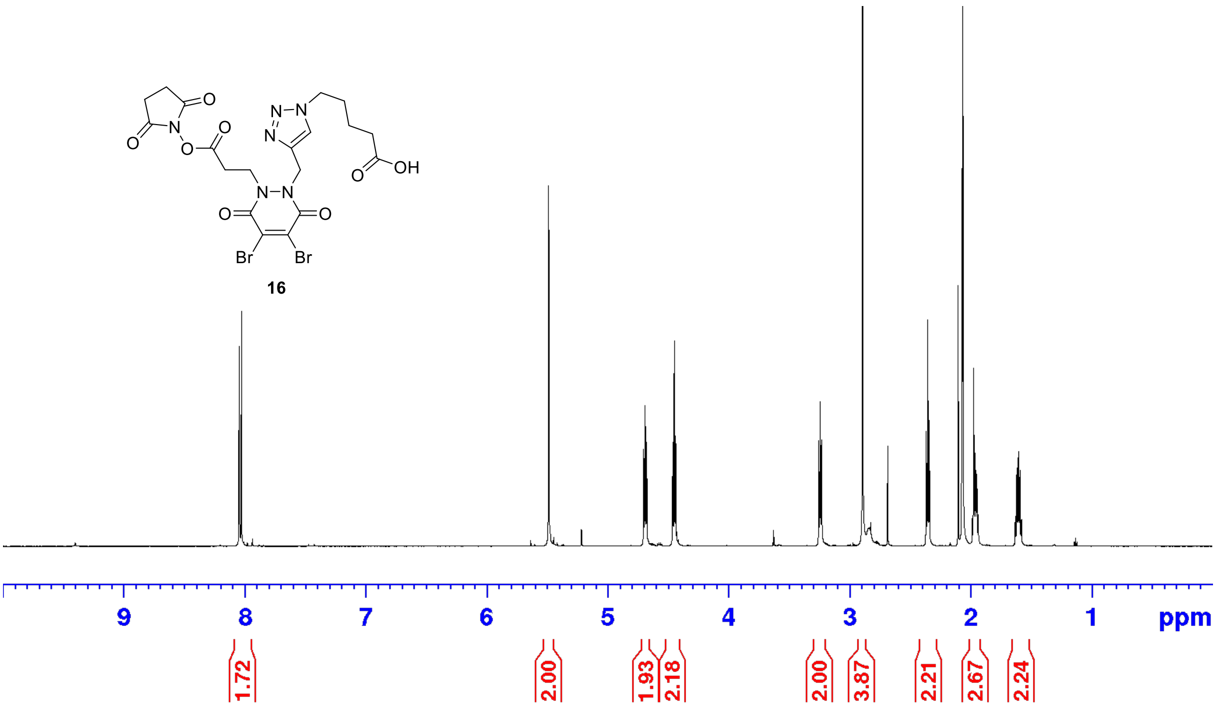

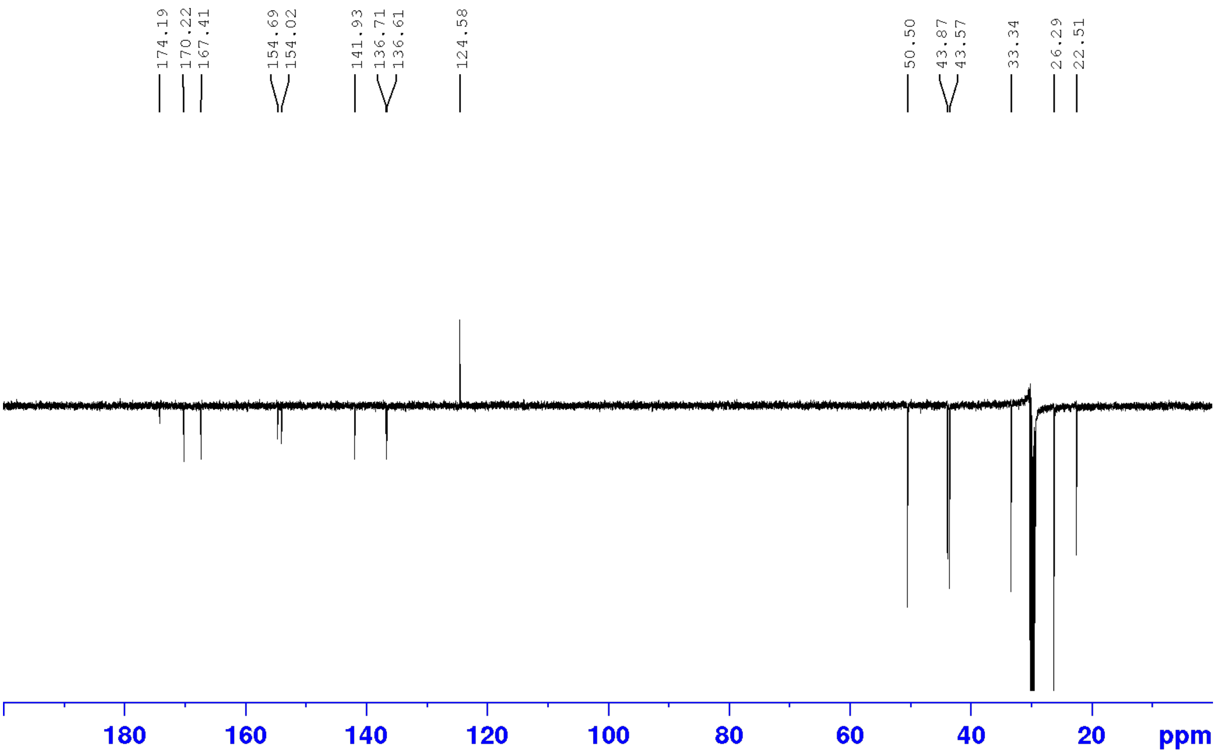

^{1}H and ^{13}C NMR-spectra of compound **18**

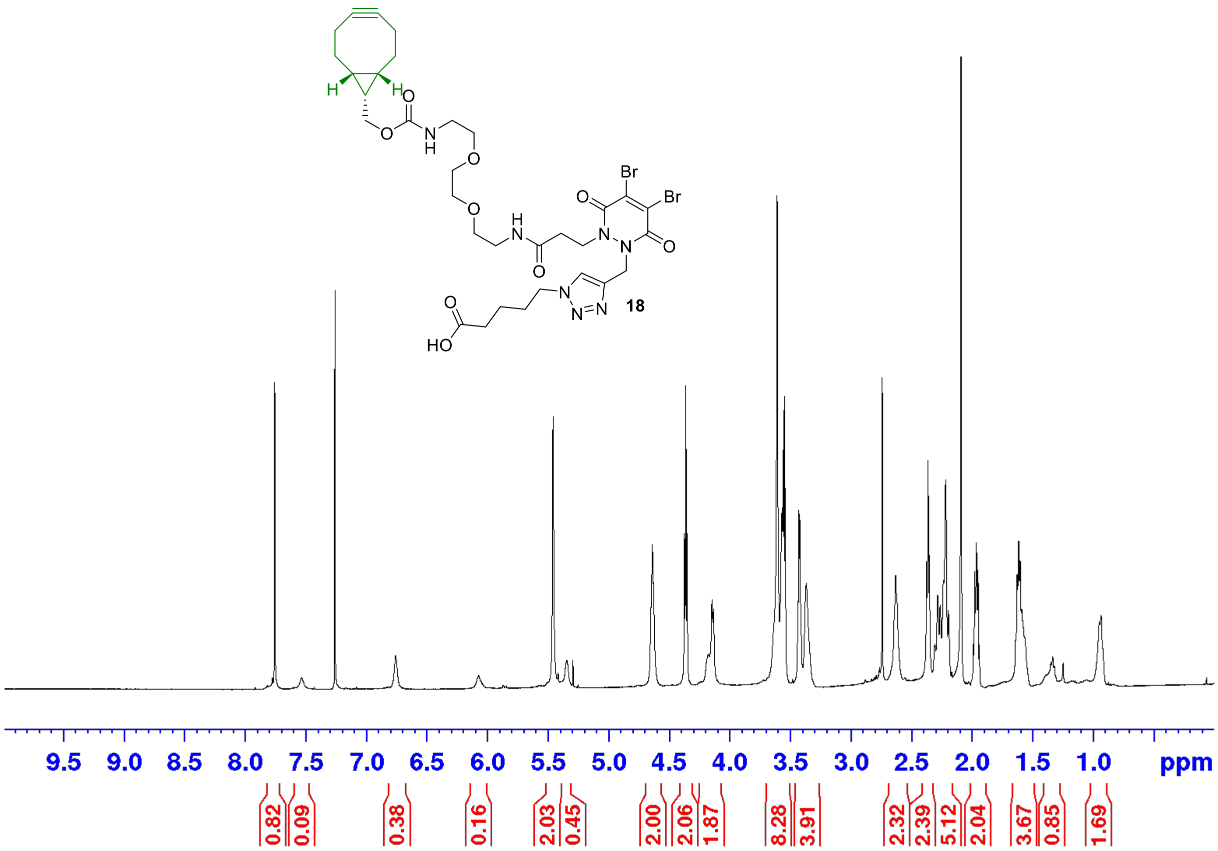

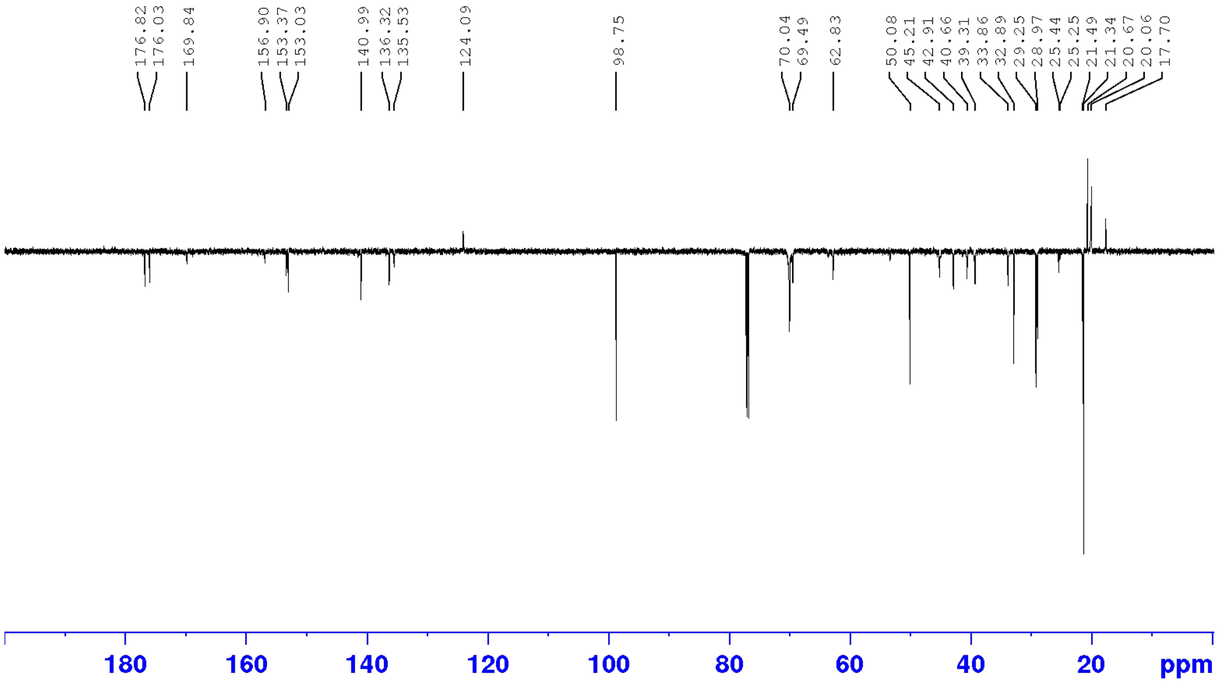

^{1}H and ^{13}C NMR-spectra of compound **19**

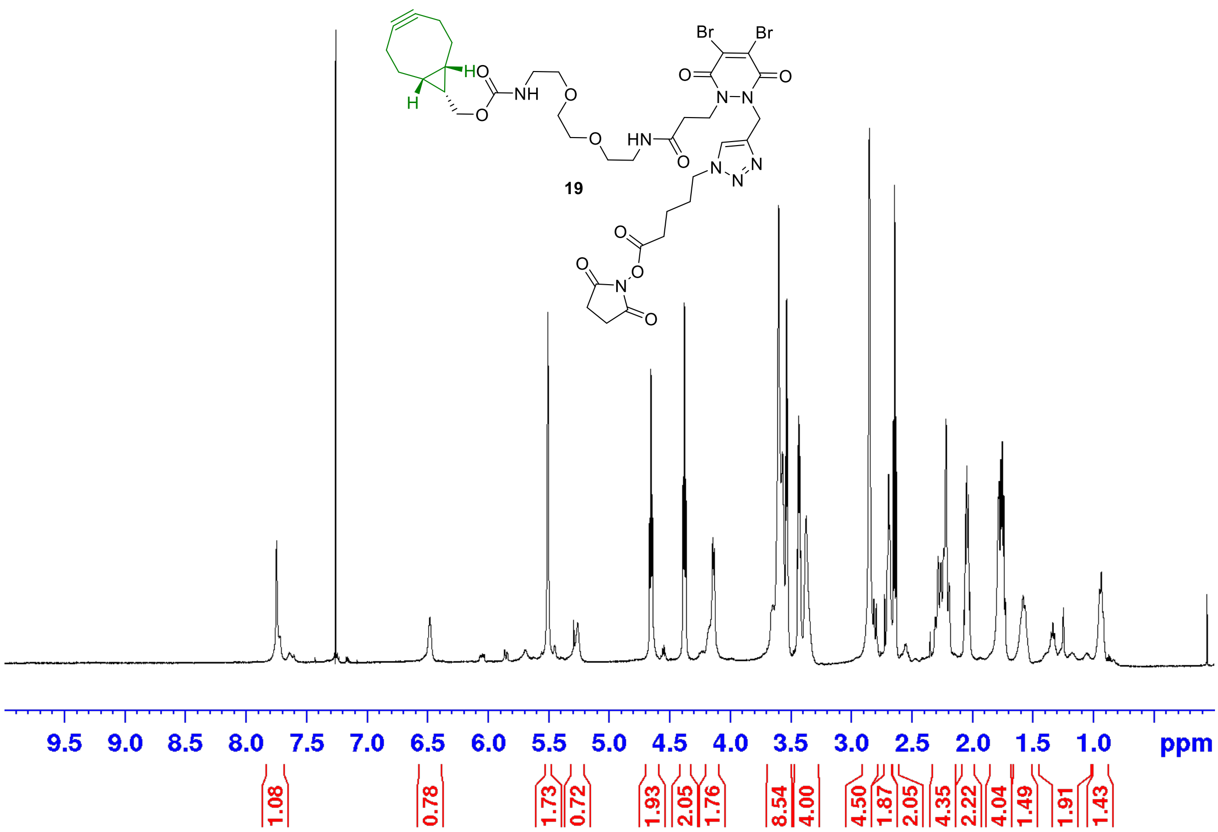

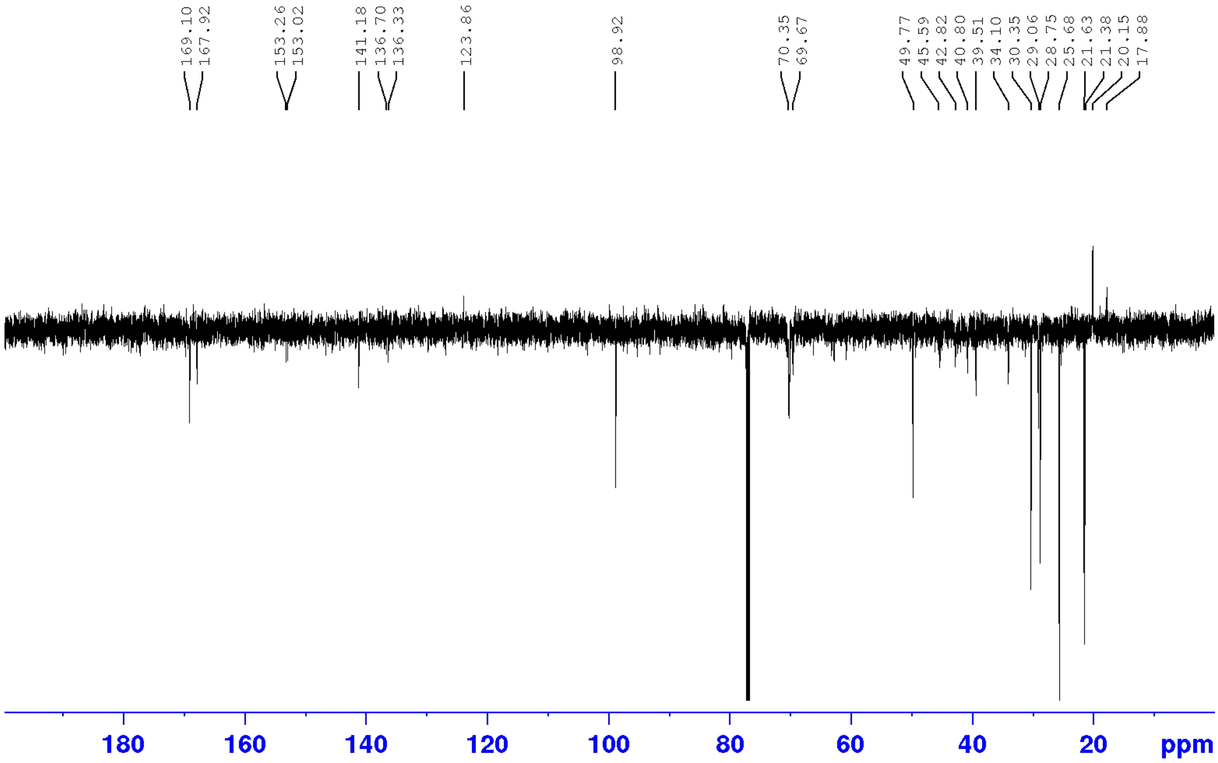

^{1}H, ^{13}C, HSQC NMR-spectra and HPLC-ELSD (C18 column) of compound **21**

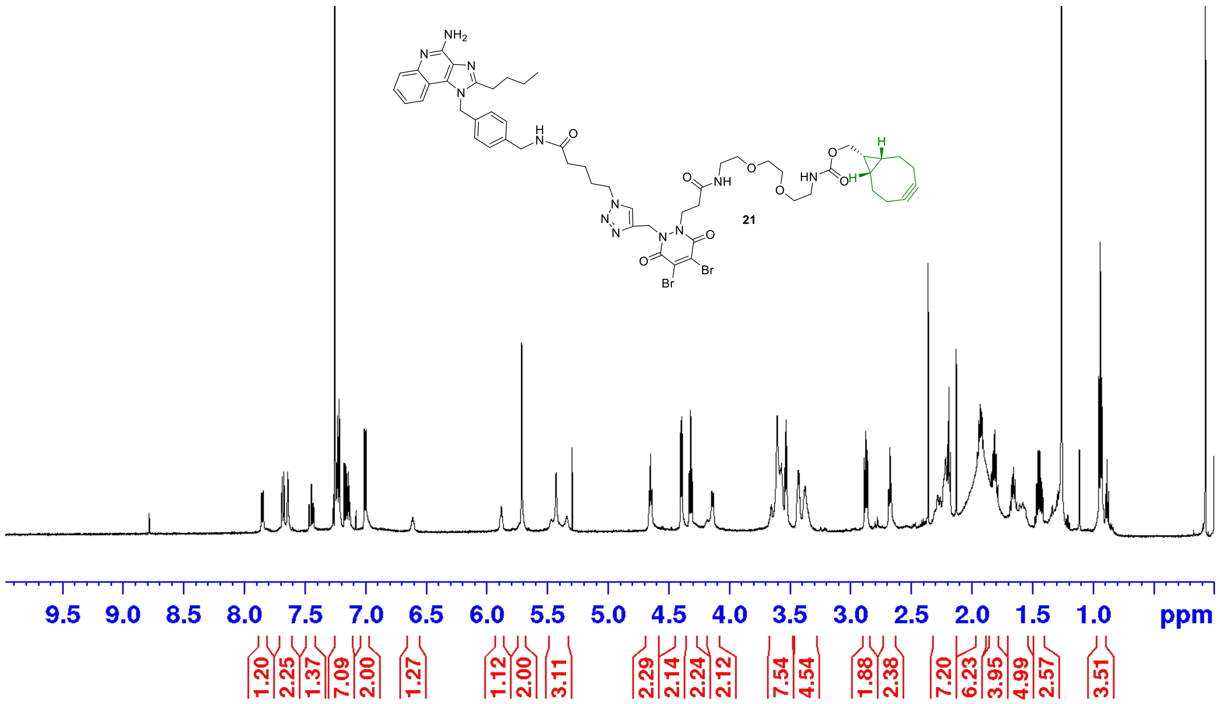

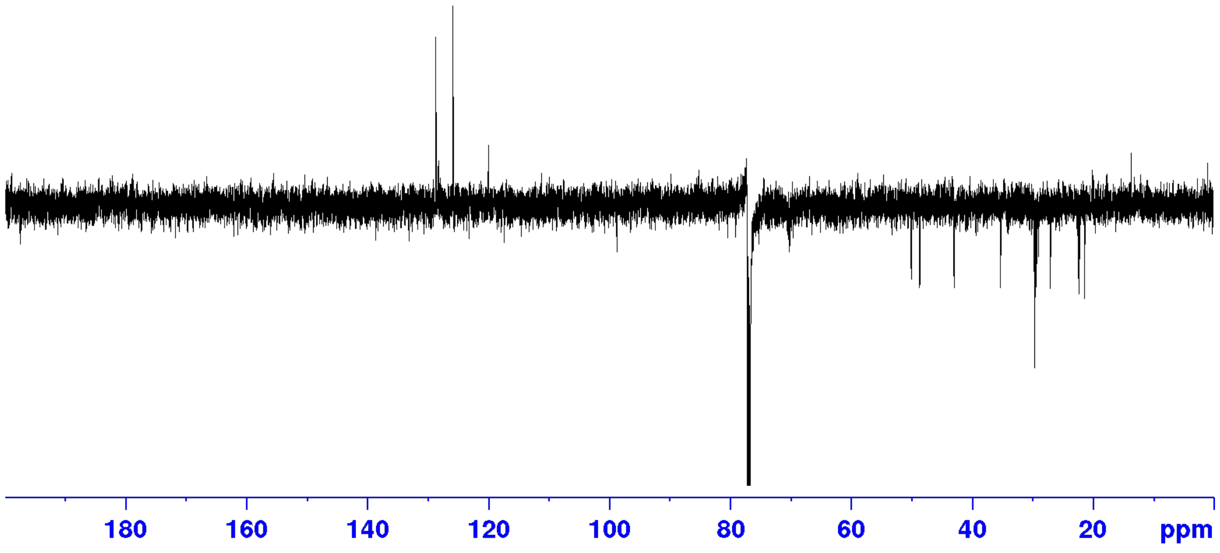

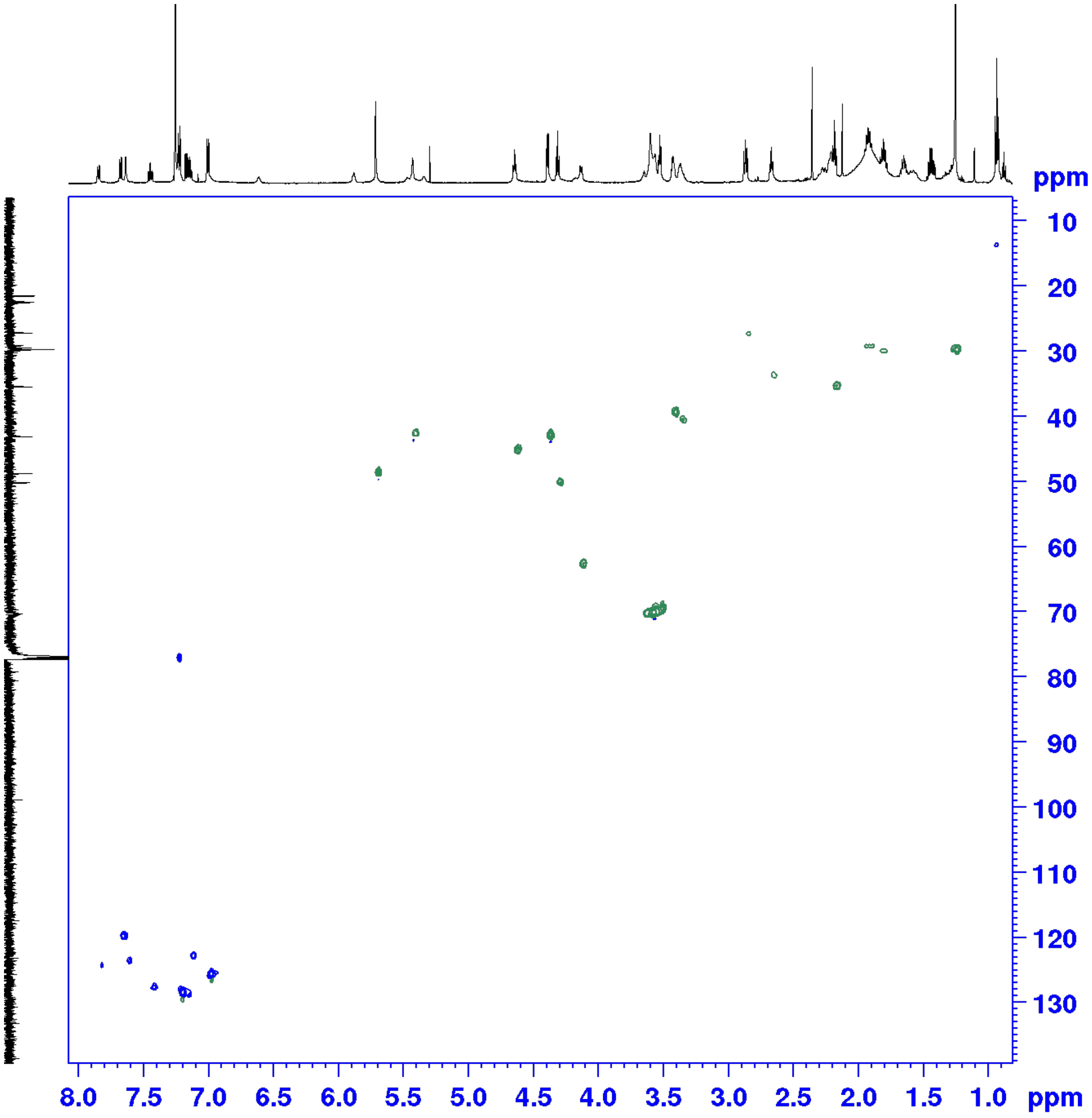
ppm
10
20
30
40
50
60
70
80
90
100
110
120
130
8.0 7.5 7.0 6.5 6.0 5.5 5.0 4.5 4.0 3.5 3.0 2.5 2.0 1.5 1.0
ppm

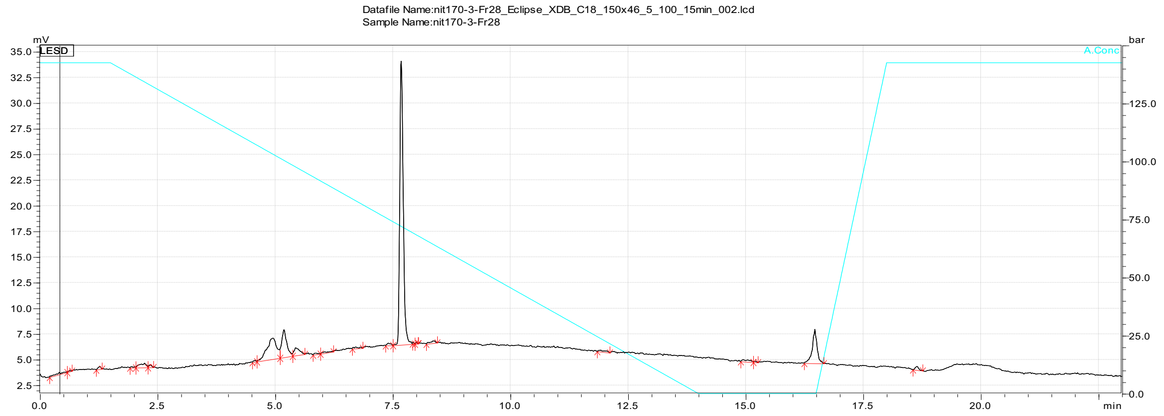
Datafile Name:nit170-3-Fr28_Eclipse_XDB_C18_150x46_5_100_15min_002.lcd
Sample Name:nit170-3-Fr28
mV
LESD
bar
A.Conc
35.0
32.5
30.0
27.5
25.0
22.5
20.0
17.5
15.0
12.5
10.0
7.5
5.0
2.5
125.0
100.0
75.0
50.0
25.0
0.0
0.0 2.5 5.0 7.5 10.0 12.5 15.0 17.5 20.0 min

^{1}H, HSQC NMR spectra and HPLC-ELSD (C4 column) of compound **S5**

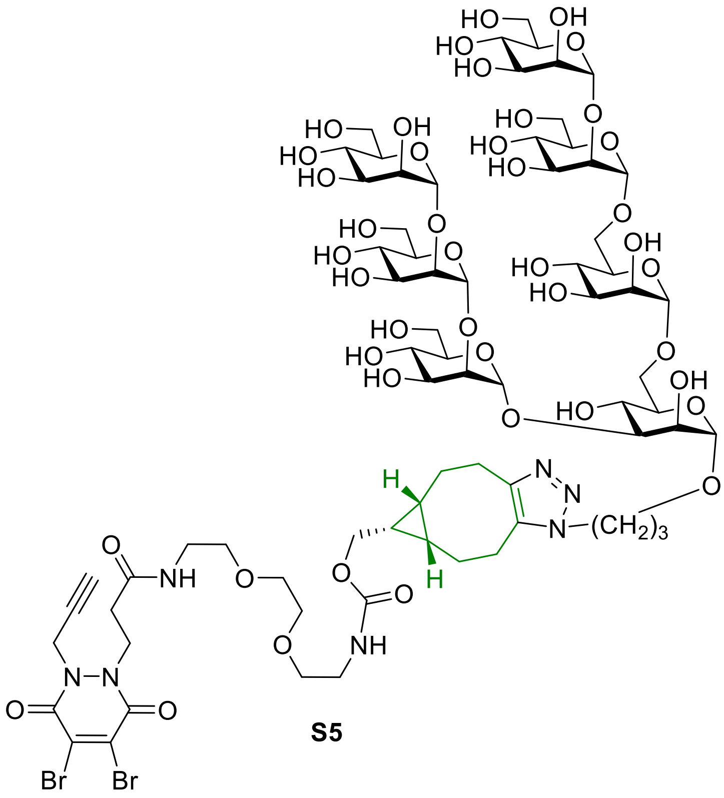

S5

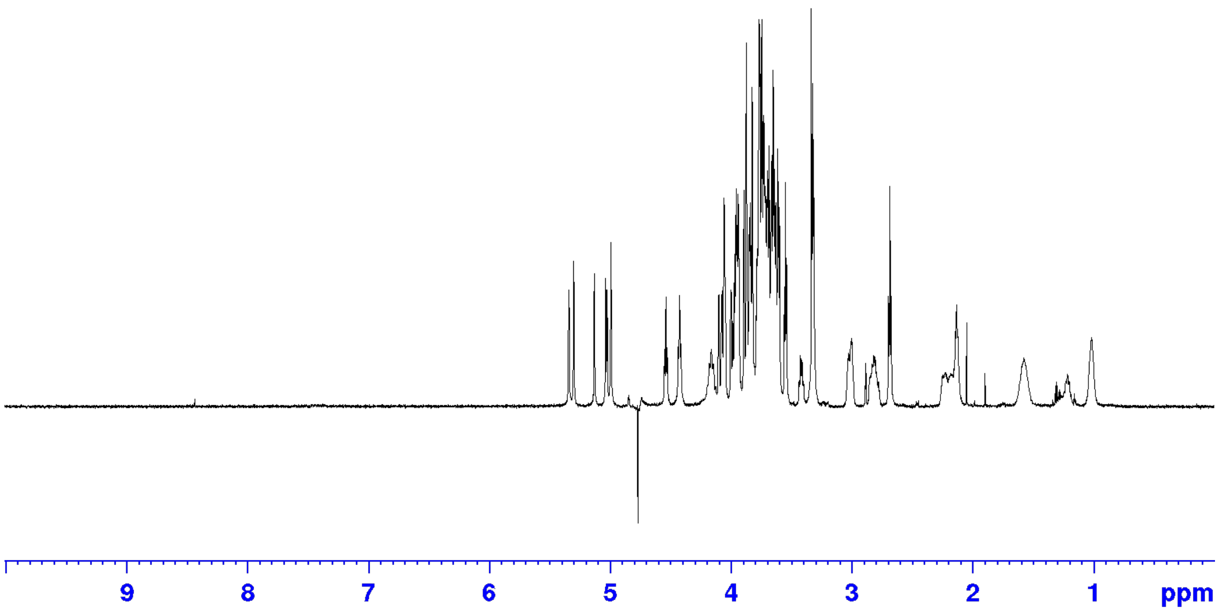

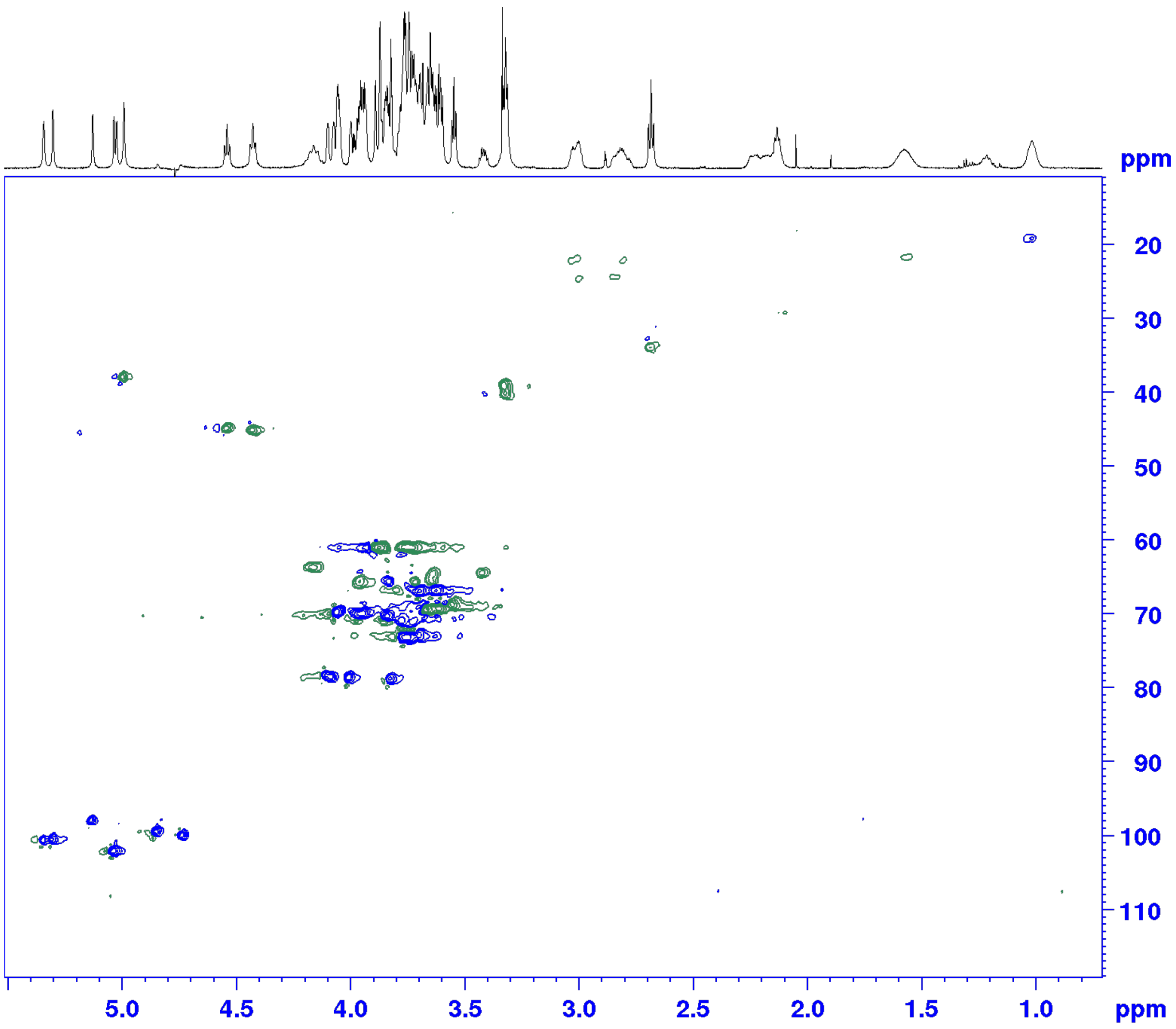
ppm
20
30
40
50
60
70
80
90
100
110
5.0
4.5
4.0
3.5
3.0
2.5
2.0
1.5
1.0
ppm

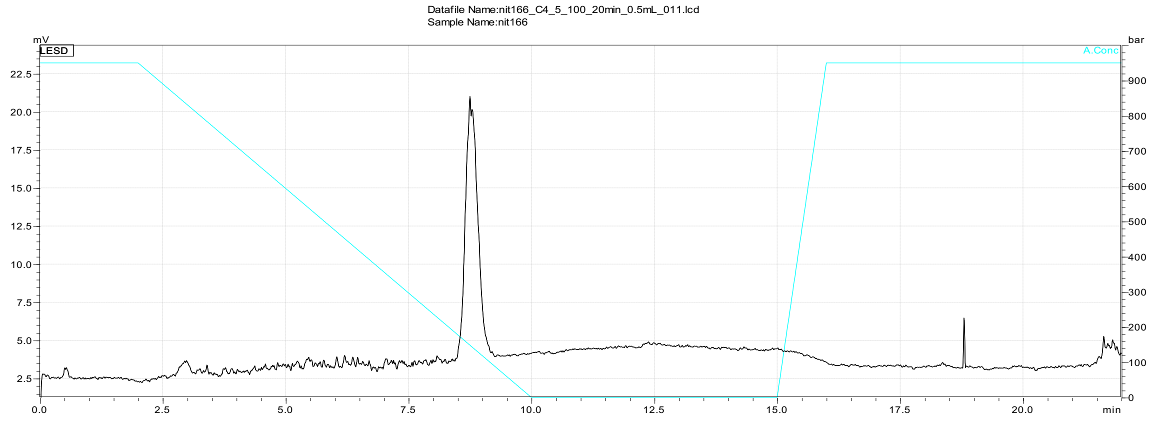
Datafile Name:nit166_C4_5_100_20min_0.5mL_011.lcd
Sample Name:nit166
mV
LESD
A.Conc
bar
22.5
20.0
17.5
15.0
12.5
10.0
7.5
5.0
2.5
900
800
700
600
500
400
300
200
100
0
0.0
2.5
5.0
7.5
10.0
12.5
15.0
17.5
20.0
min

^{1}H NMR spectrum and HPLC-ELSD (C4 column) of compound **23**

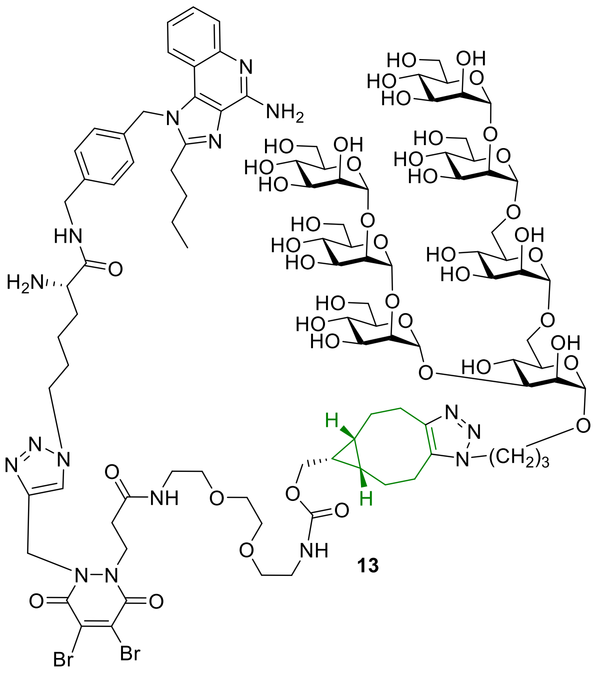

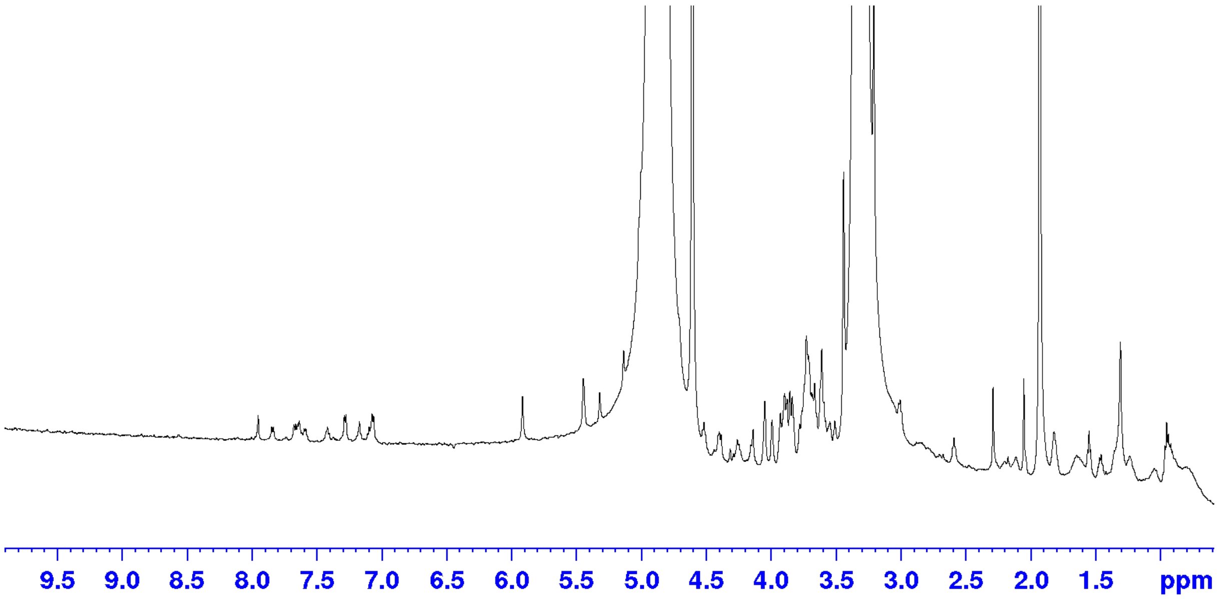

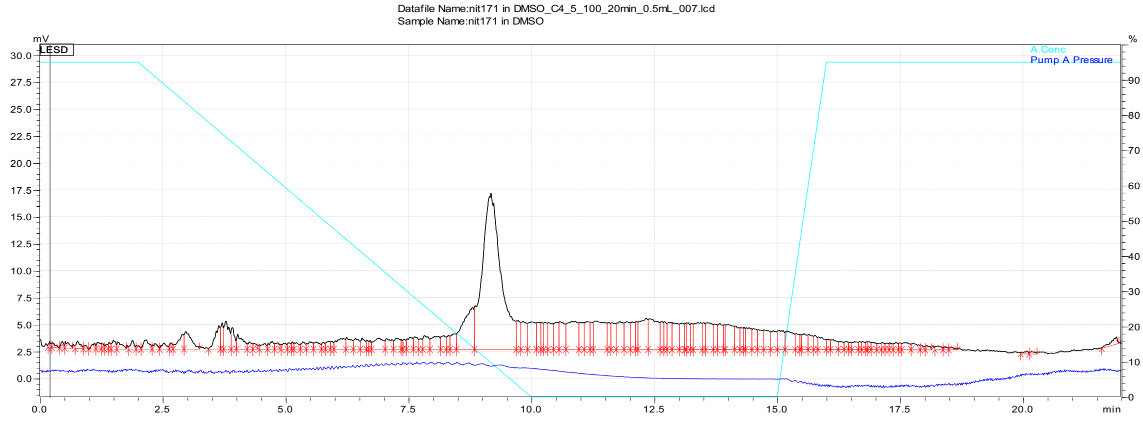

4. Site-selective protein conjugation

4.1. Conjugation of CRM_{197} with linker 1

4.1.1. Installation of linker 1 on CRM_{197}

For assessment of best reaction conditions, the linker introduction was screened with two different methods:

Method 1: CRM_{197} (100 µg; 1.7 nmol) was dissolved in 100 µL of buffer, followed by the addition of a solution of TCEP × HCl (5 µL, 3.5 mM in water) and is was stirred at 37 °C for 3 h. Then, the solution was spin filtered (cut-off 10 kDa, 3 times against fresh buffer) to a final Volume of 100 µL, linker **1** (2 µL; 17 mM in DMSO) was added and stirring was continued for the indicated time. The reaction mixture was spin filtered against water as described above and was then analyzed by ESI-QTOF-MS.

Method 2: CRM_{197} (100 µg; 1.7 nmol) was dissolved in 100 µL of buffer, followed by the addition of linker **1** (2 µL; 17 mM in DMSO) and after stirring for 5 min a solution of TCEP × HCl (5 µL, 3.5 mM in water; 10 eq.) was added. It was stirred at the indicated temperature and time and then the sample was spin filtered against water (cut-off 10 kDa, 3 times), followed by analysis of the reaction mixture by ESI-QTOF-MS.

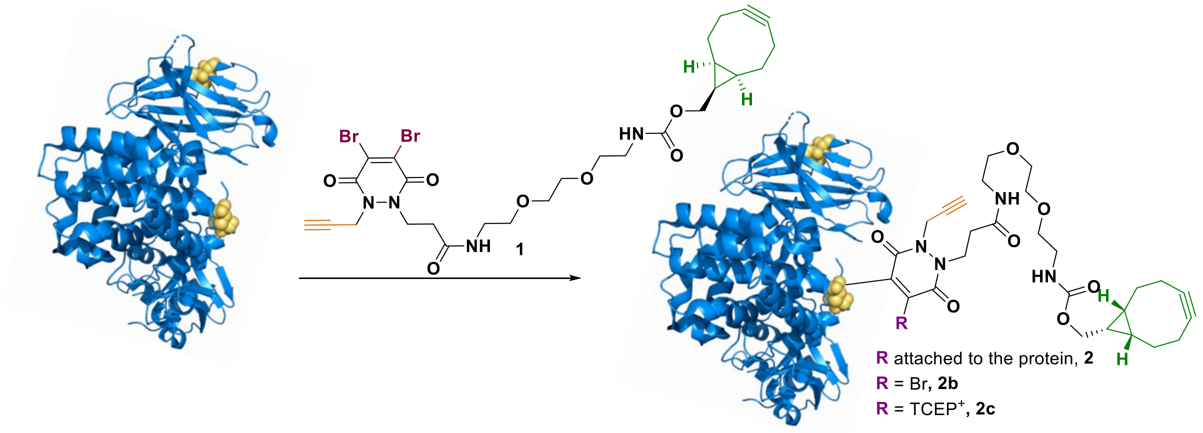

Table S1. Conditions screening towards **2**

Entry	method	Buffer (pH)	Additive	T [°C]	t [h]	Product distribution			
						**CRM$_{197}$**	**2**	**2b**	**2c**
**1**	1	PBS (8)	-	37	16	100	-	-	-
**2**	1	PBS (7.4)	-	37	16	100	-	-	
**3**	1	BBS (8)	-	37	16	100	-	-	
**4**	1	PBS (8)	5 % MeCN	37	16	66	-	34	
**5**	1	BBS (8)	5 % MeCN	37	16	very diverse 2-3 linker etc.			
**6**	2	PBS (8)	-	37	16	100	-	-	
**7**	2	BBS (8)	-	37	16	-	23	-	77
**8**	2	BBS (8)	-	22	16	-	30	-	70
**9**	2	BBS (8)	-	4	16	26	32	-	42
**10**	2	BBS (8)	5 % MeCN	37	16	not detectable/decomposition			
**11**[1]	2	BBS (8)	-	37	16	-	-	-	100
**12**	2	BBS (8)	-	37	16	-	-	-	100
**13**[2]	2	BBS (8)	-	37	16	28	-	42	30
**14**	2	BBS (8)	-	37	2	25	-	-	75
**15**	2	BBS (8)	$NaBH_4$	37	16	17	-	73	10
**16**	2	25 mM TRIS (8)	-	37	16	13	43	13	31
**17**	2	25 mM TRIS (8)	0.5 M Urea	37	16	-	-	-	100
**18**	2	25 mM TRIS (8)	0.5 M Sucrose	37	16	54	16	11	19
**19**	2	25 mM TRIS (8)	0.3 M NaCl	37	16	not detectable/decomposition			
**20**	2	25 mM TRIS (8)	0.2 M Glycine	37	16	100	-	-	-
**21**	2	25 mM TRIS (8)	0.5 % Tween 20	37	16	-	25	-	75
**22**	2	25 mM TRIS (8)	0.1 M Na_2SO_4	37	16	32	18	19	31
**23**	2	0.25 M TRIS (8)	-	37	16	-	95	5	-
**24**	2	0.25 M HEPES (8)	-	37	16	34	46	19	-
**25**	2	25 mM HEPES (8)	-	37	16	20	19	-	61
**26**	2	0.25 M TRIS (8)	-	37	7	16	63	13	-
**27**	2	0.25 M TRIS (8)	-	22	16	-	78	22	-
**28**	2	0.25 M TRIS (8)	-	4	16	6	85	9	-
**29**[3]	2	0.25 M TRIS (8)	-	4	40	-	98	-	2

1 50 eq. of Linker was added

2 2.5 eq. Of TCEP were sued

3 reaction was performed in a bigger scale (2 mg)

SDS-PAGE:

Entries **23** and **26-28** were additionally analyzed by SDS-PAGE

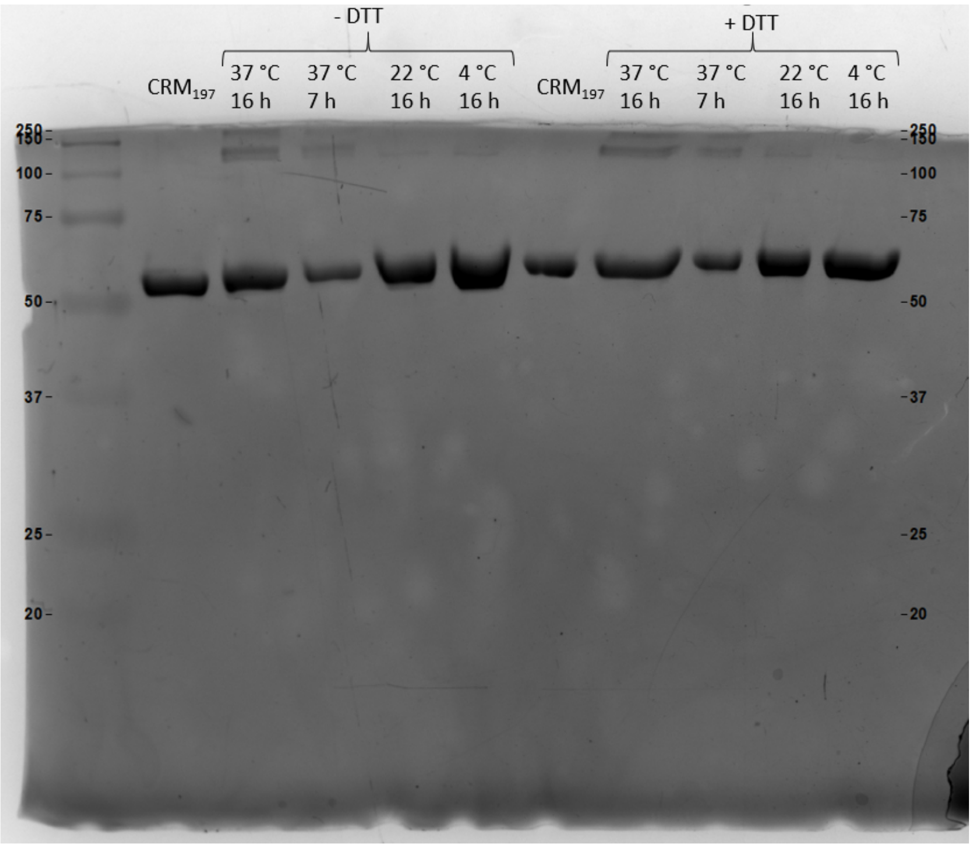

SI Figure 1 SDS-PAGE (8 %, 200 V, 40 min, stained with Coomassie) of **2** under selected reaction conditions with and without DTT.

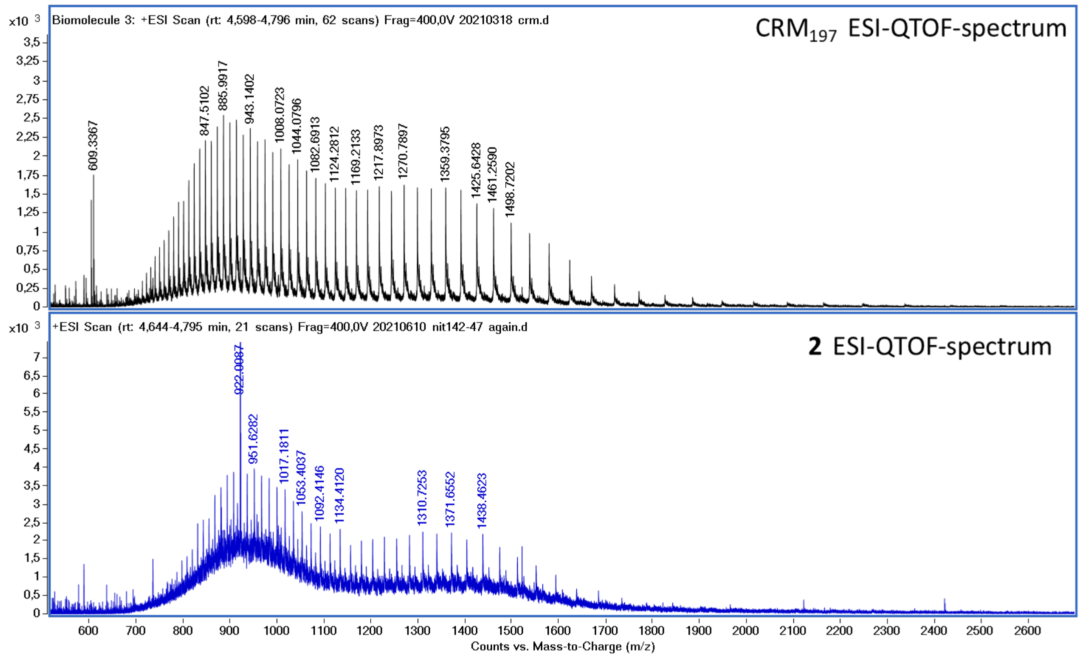

SI Figure 2 ESI-QTOF spectra of CRM_{197} and **2**

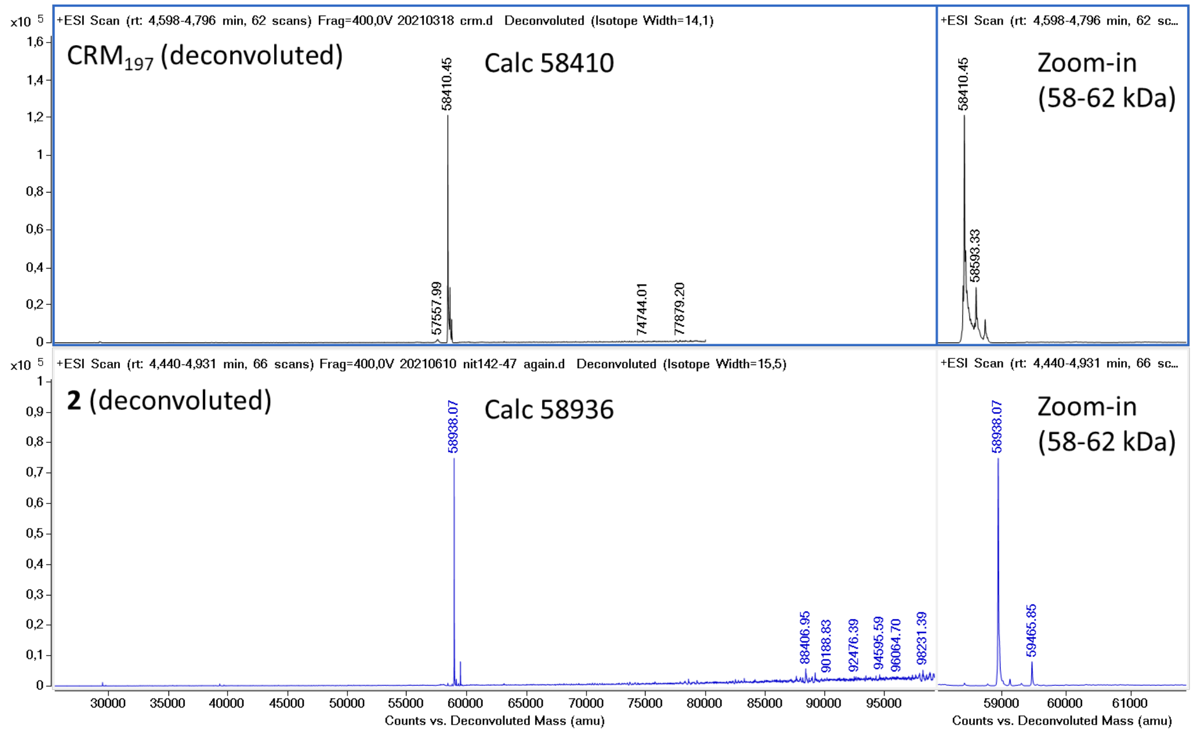

SI Figure 3 deconvoluted ESI-QTOF spectra of CRM_{197} and **2**

Optimized procedure: CRM_{197} (2 mg; 34.2 nmol) was dissolved in TRIS-buffer (2 mL; 250 mM, pH = 8), followed by the addition of the linker (40 µL; 10 mg/mL in DMSO; 684.9 nmol) and after stirring for 5 min it was cooled down to 4 °C. Then, a solution of TCEP × HCl (50 µL, 2 mg/mL in water; 342.5 nmol) was added and stirring was continued for 40 h at 0 °C. After that, the sample was spin filtered against

water (cut-off 10 kDa, 3 times) which afforded 380 µL aqu. solution of **2**. Nanodrop analysis (E 1% = 10.7) revealed a concentration of 5 mg/mL which refers to a total protein recovery of 1.9 mg (95 %) and analysis by ESI-QTOF-MS revealed product purity of > 95 %

4.1.2. Synthesis of conjugate 8

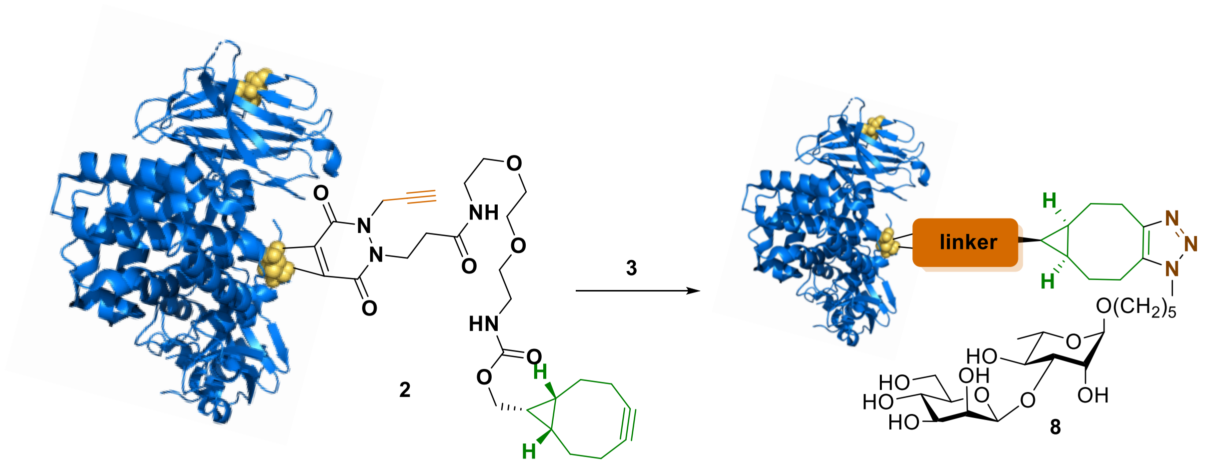

To a solution of linker conjugated CRM **2** (12 µL; 8.4 mg/mL in water; 1.69 nM) was added 90 µL PBS-buffer (pH = 7.4). Then, a solution of **3** (1.5 µL; 5 mg/mL in water; 16.9 nM) was added and it was stirred at room temperature for 16 h. After that, the sample was spin filtered against water (cut-off 10 kDa, 3 times) and analysed by ESI-QTOF-MS which revealed full conversion to the desired product.

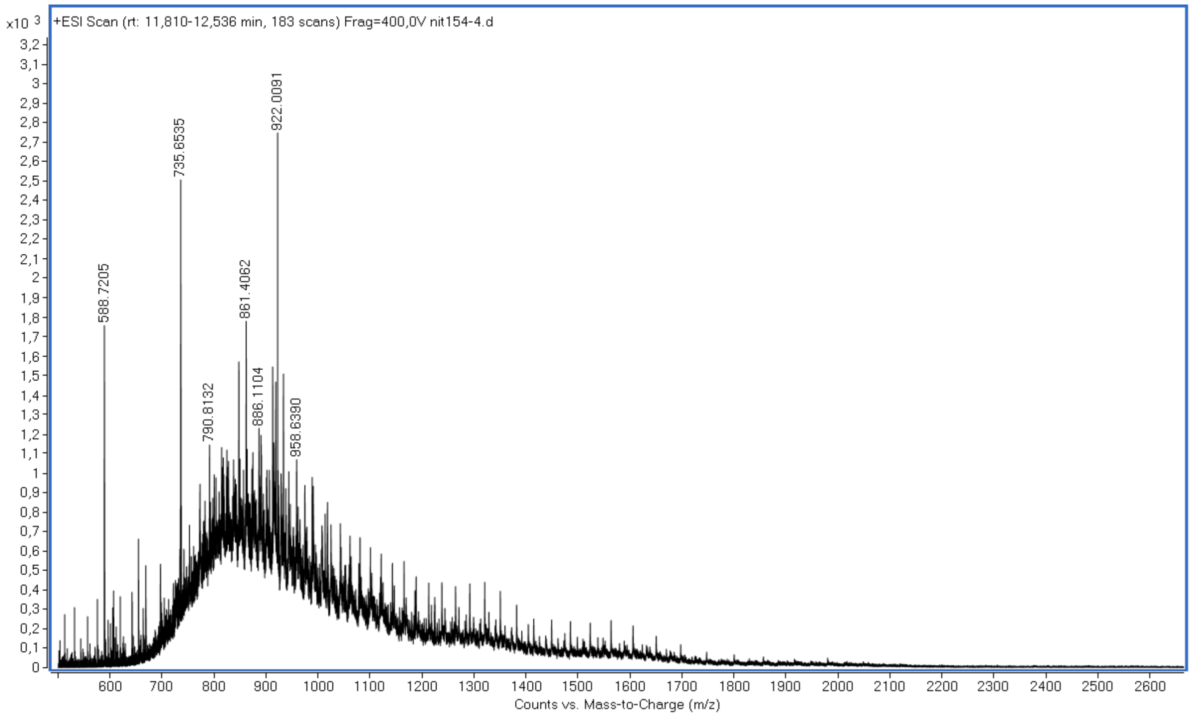

SI Figure 4 ESI-QTOF spectra of **8**

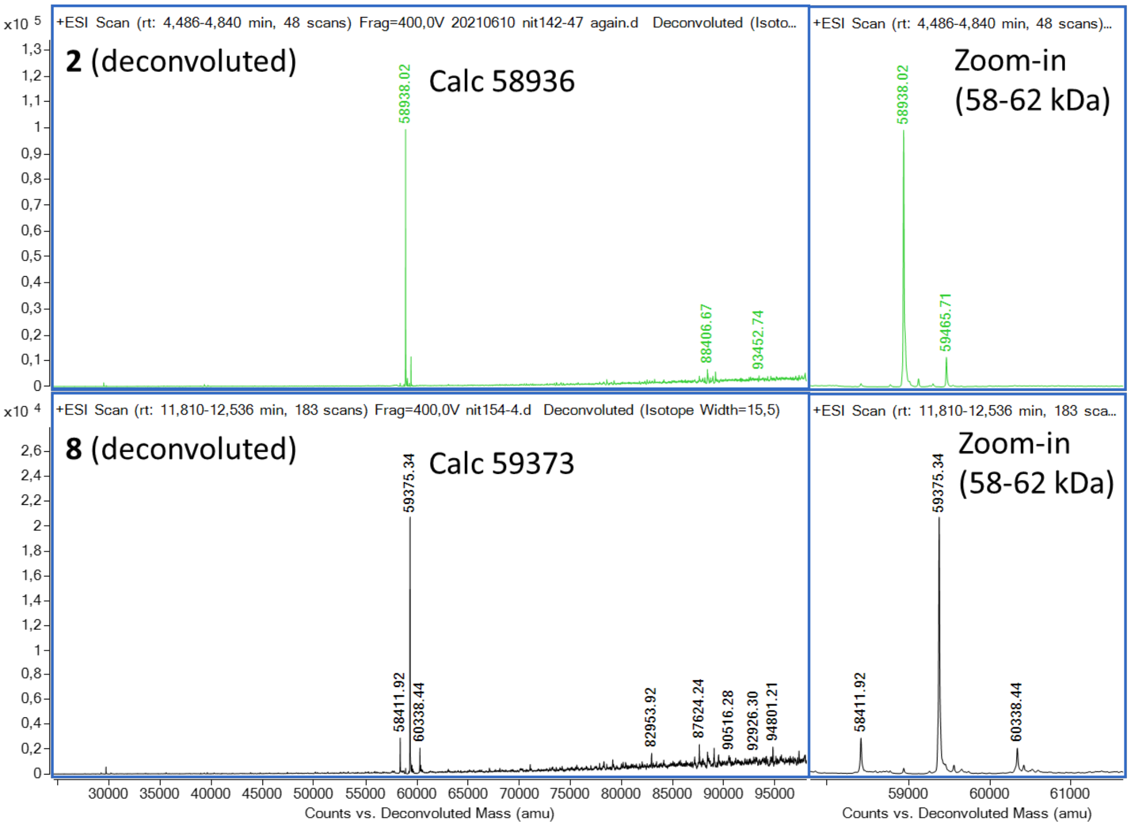

SI Figure 5 Deconvoluted ESI-QTOF spectra of **2** and **8**

4.1.3. Synthesis of conjugate 9

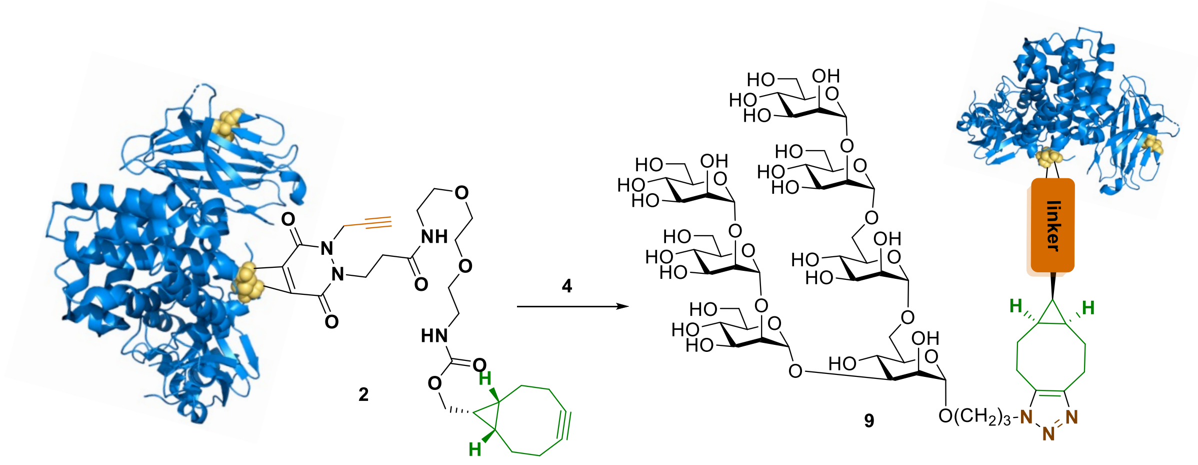

To a solution of linker conjugated CRM **2** (10 µL; 5 mg/mL in water; 0.85 nM) was added 40 µL PBS-buffer (pH = 7.4). Subsequently, a solution of **4** (2 µL; 10 mg/mL in water; 16.9 nM) was added and it was stirred at 37 °C for 16 h. After that, the sample was spin filtered against water (cut-off 10 kDa, 3 times) which afforded 90 µL aqu. solution. Nanodrop analysis (E 1% = 10.7) revealed a concentration

of 0.39 mg/mL which refers to a total protein recovery of 35 µg (70 %) and analysis by ESI-QTOF-MS revealed full conversion to the desired product.

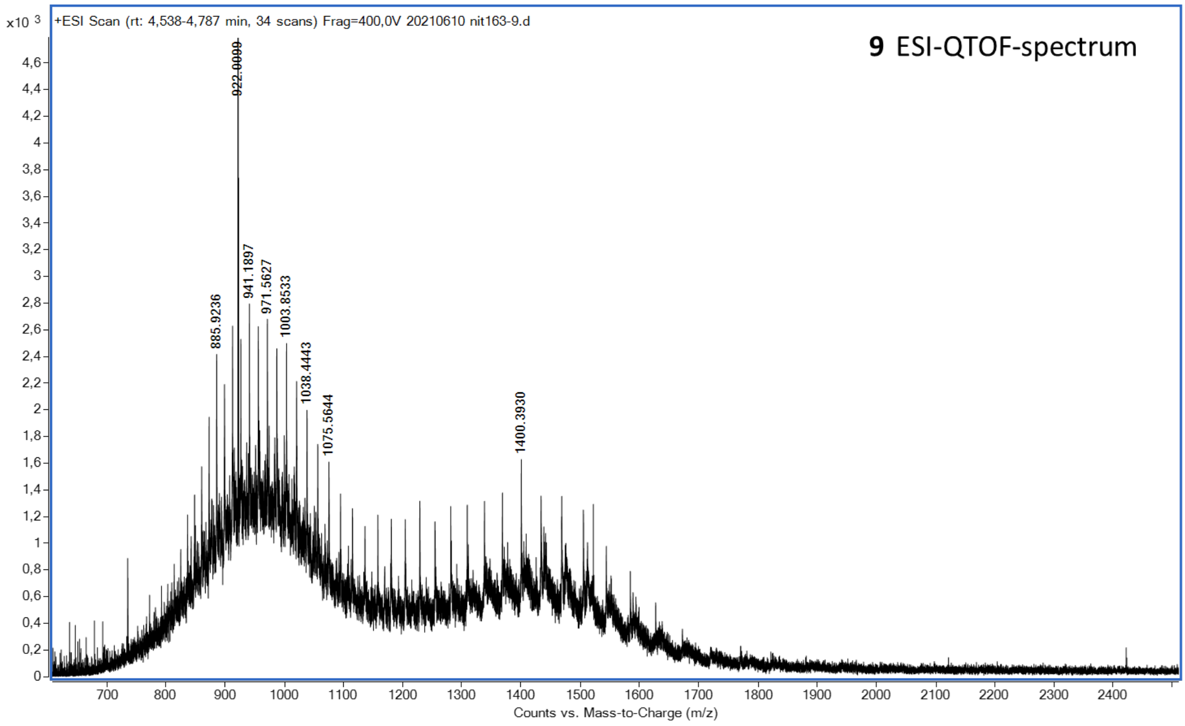

SI Figure 6 ESI-QTOF spectra of **9**

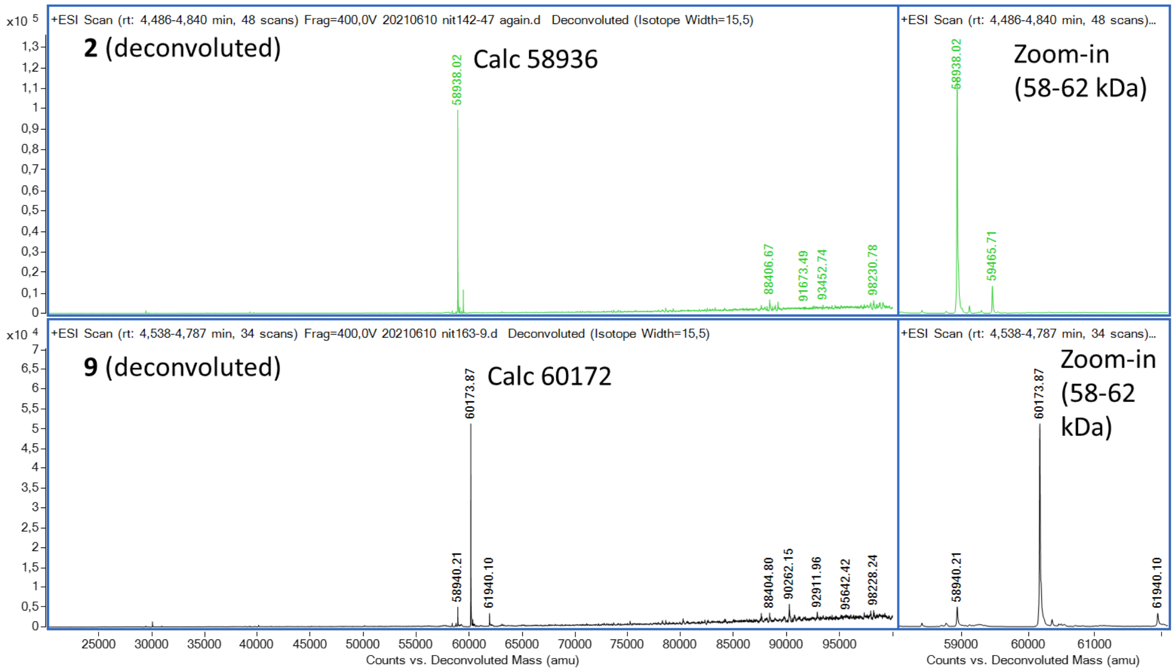

SI Figure 7 Deconvoluted ESI-QTOF spectra of **2** and **9**

4.1.4. Synthesis of conjugate 10

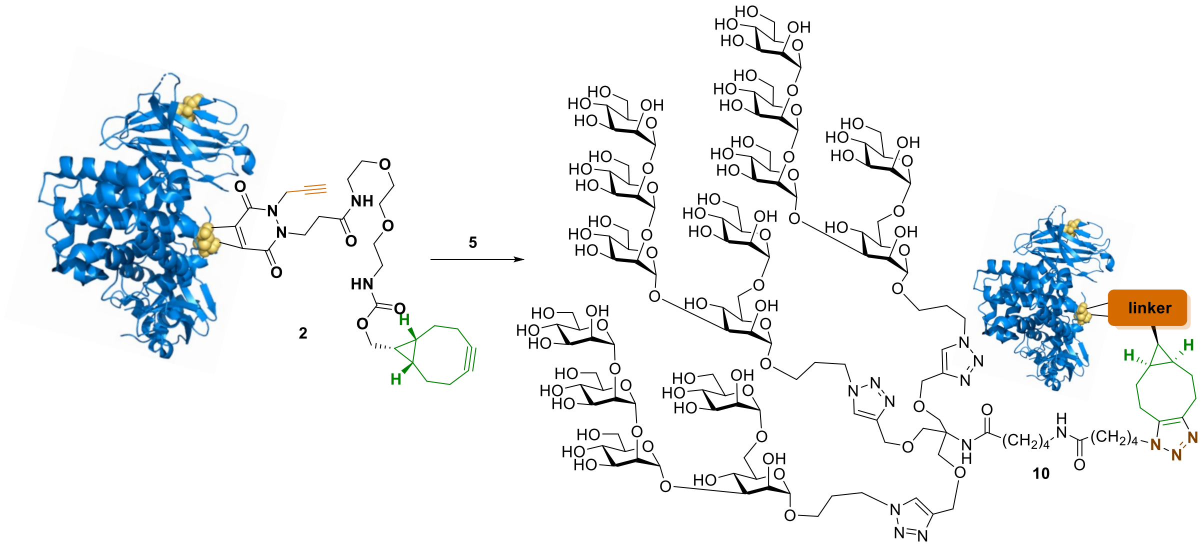

To a solution of linker conjugated CRM **2** (20 µL; 5 mg/mL in water; 1.69 nM) was added 80 µL TRIS-buffer (0.25 M, pH = 8). Subsequently, a solution of **5** (100 µL; 1 mg/mL in water; 33.79 nM) was added and it was stirred at 37 °C for 6 days. After that, the sample was spin filtered against water (cut-off 10 kDa, 3 times) which afforded 100 µL aqu. solution. Nanodrop analysis (E 1% = 10.7) revealed a protein concentration of 0.81 mg/mL which refers to a total protein recovery of 81 µg (81 %) and analysis by ESI-QTOF-MS revealed a substrate conversion of 94 %.

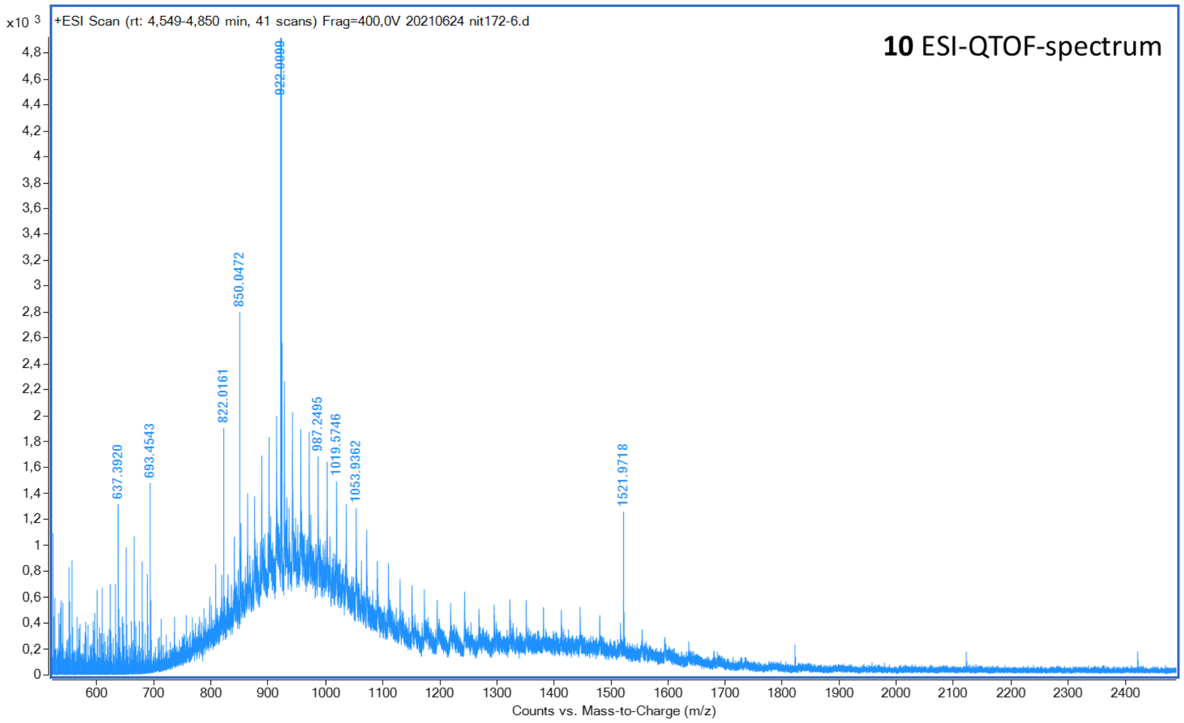

SI Figure 8 ESI-QTOF spectra of **10**

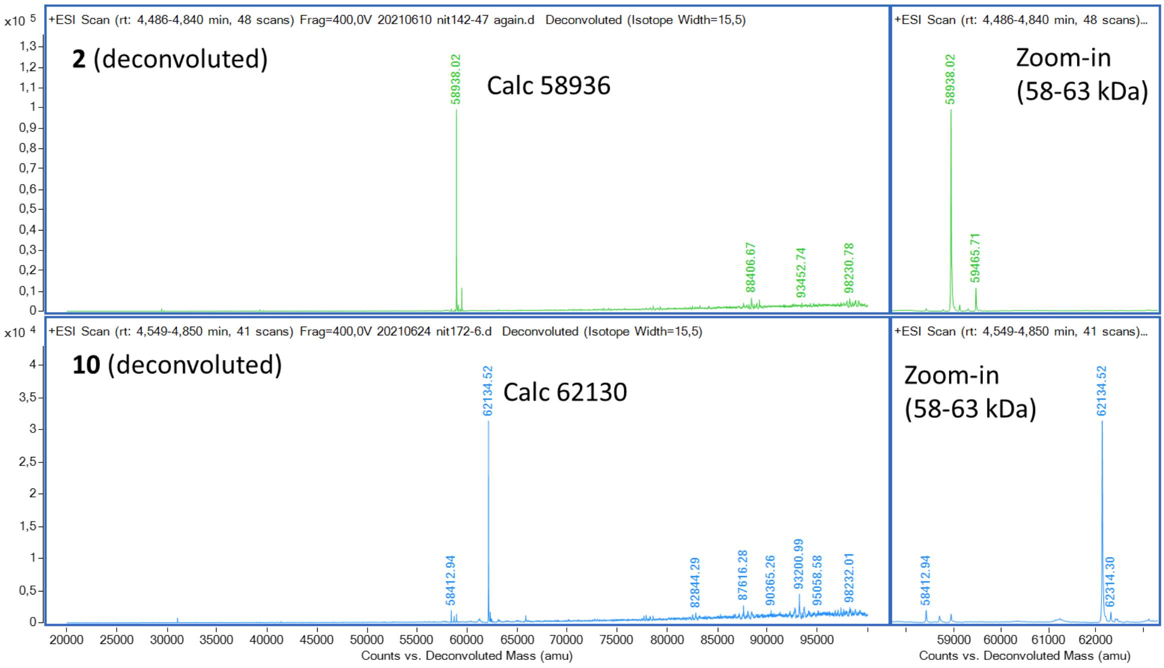

SI Figure 9 Deconvoluted ESI-QTOF spectra of **2** and **10**

4.1.5. Synthesis of conjugate 11

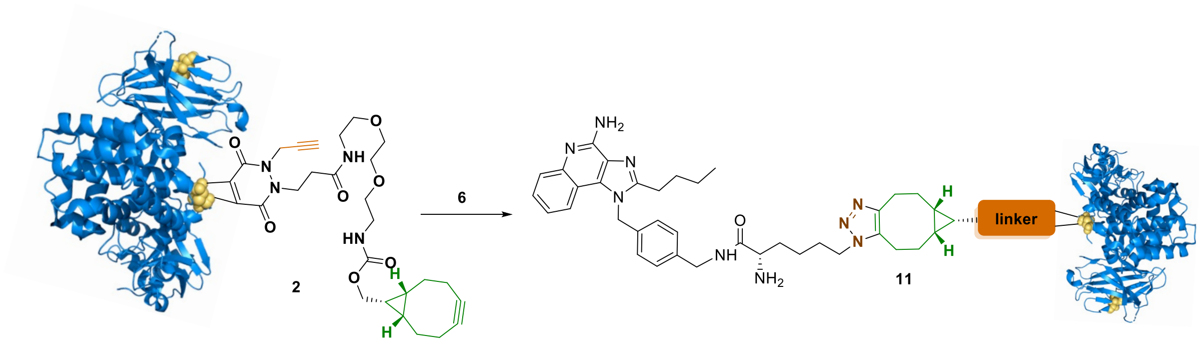

To a solution of linker conjugated CRM **2** (80 µL; 8 mg/mL in water; 11.0 nM) was added 500 µL PBS-buffer (pH = 7.4). Then, a solution of **6** (8.6 µL; 5 mg/mL in DMSO; 76.9 nM) was added and it was stirred at 22 °C for 16 h. After that, the sample was spin filtered against water (cut-off 10 kDa, 3 times) which afforded 300 µL aqu. solution. Nanodrop analysis (E 1% = 10.7) revealed a concentration of 2.0 mg/mL which refers to a total protein recovery of 600 µg (92 %) and analysis by ESI-QTOF-MS revealed full conversion to the desired product.

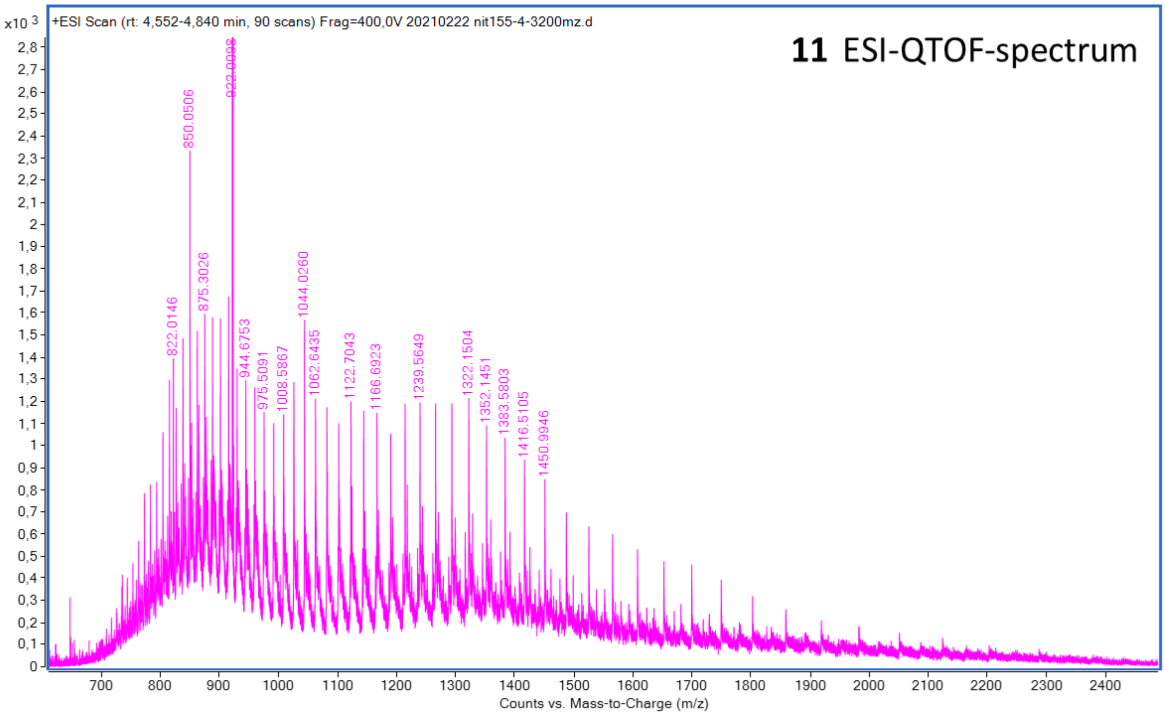

SI Figure 10 ESI-QTOF spectra of **11**

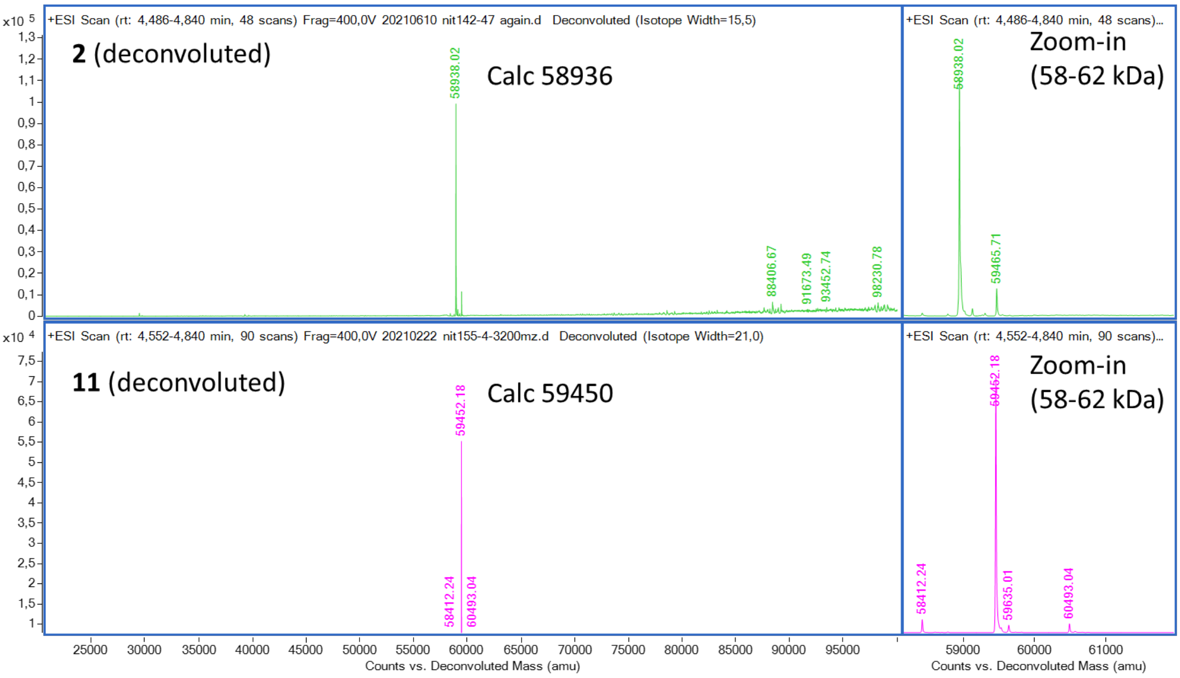

SI Figure 11 Deconvoluted ESI-QTOF spectra of **2** and **11**

4.1.6. Synthesis of conjugate 12

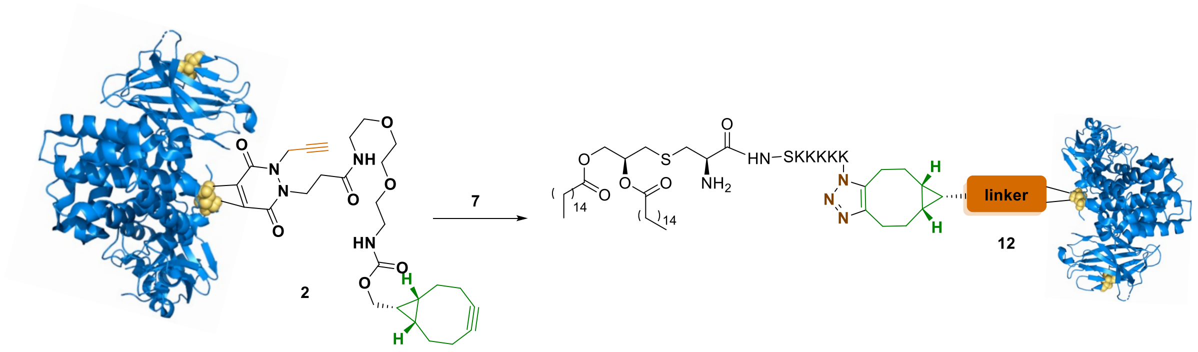

Table S2 Condition screening towards conjugate **12**

Entry	Buffer (pH)	additive (%)	**11** [eq.]	Outcome	recovery
1	PBS (7.4)	DMSO (2.5)	5	precipitation	-
2	PBS (7.4)	DMSO (5)	10	precipitation	-
3	PBS (7.4)	DMSO (10)	10	precipitation	-
4	PBS (7.4)	DMSO (7)	5	no reaction	22%
5	TRIS (8)	DMSO (7)	5	full conversion	55%
6	MES (6)	DMSO (7)	5	full conversion	88%
7	MES (6)	DMSO (1)	5	full conversion	98%
8	MES (6)	DMSO (1)	25	full conversion	100%
9	MES (6)	DMSO (10)	5	full conversion	94%

Optimized procedure: To a solution of linker conjugated CRM **2** (100 µL; 5 mg/mL in water; 8.5 nM) was added 400 µL 0.1 M MES-buffer (pH = 6) . Subsequently, a solution of **7** (6 µL; 10 mg/mL in DMSO; 42.2 nM) was added and it was stirred at 22 °C for 16 h. After that, the sample was spin filtered against water (cut-off 10 kDa, 3 times) which afforded 150 µL aqu. solution. Nanodrop analysis (E 1% = 10.7) revealed a concentration of 1.93 mg/mL which refers to a total protein recovery of 290 µg (58 %) and analysis by ESI-QTOF-MS revealed full conversion to the desired product.

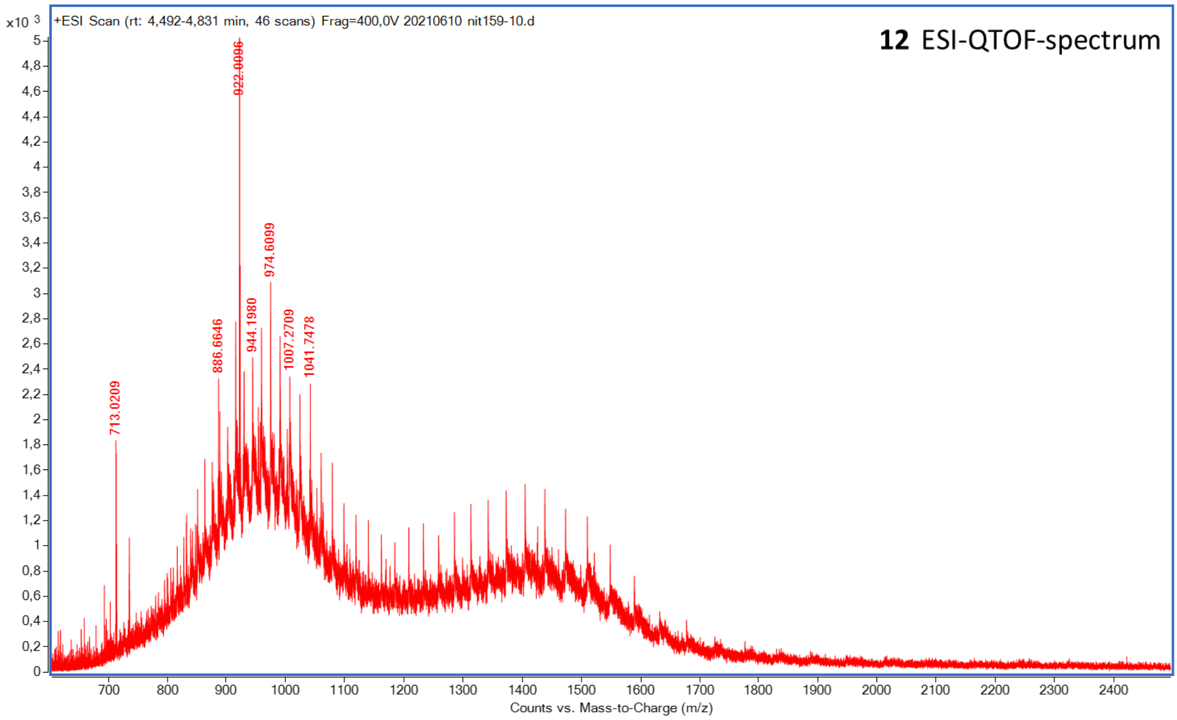

SI Figure 12 ESI-QTOF spectra of **12**

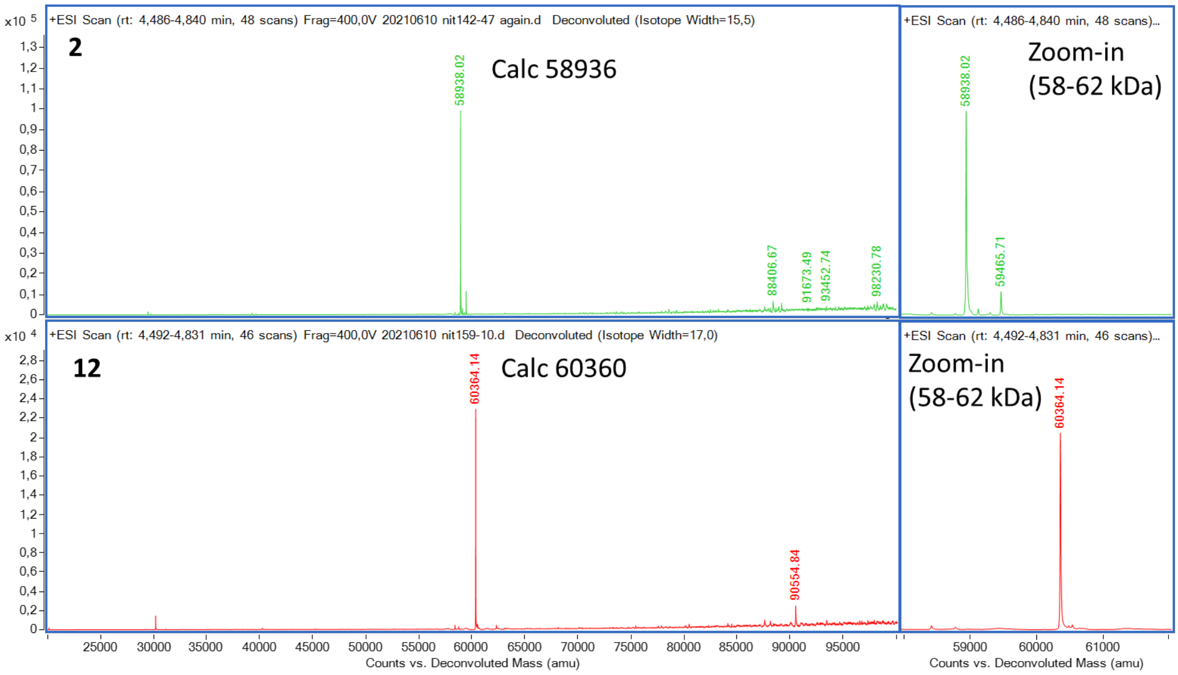

SI Figure 13 Deconvoluted ESI-QTOF spectra of **2** and **12**

4.1.7. Attempts with CuAAc and scaffold 11

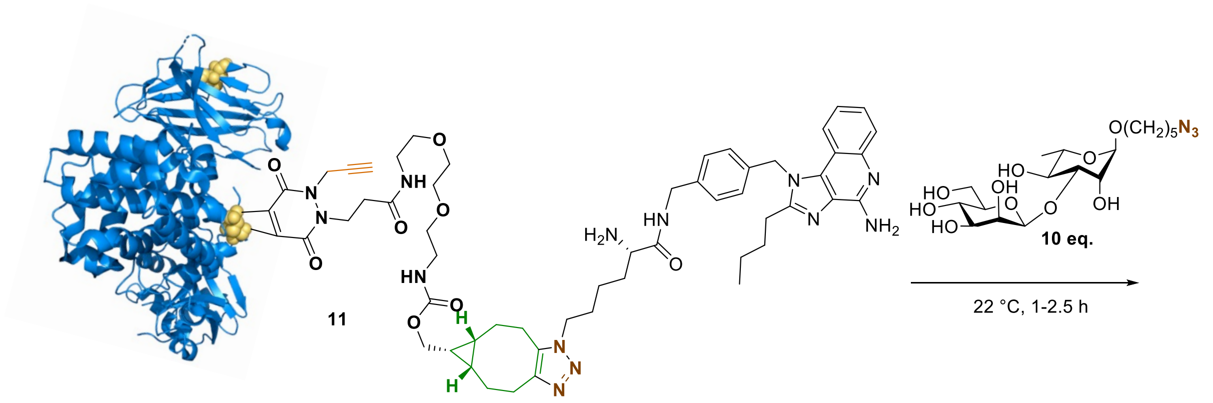

Table S3 Condition screening towards CuAAC with conjugate **11**

Entry	Buffer, pH	Cu [mM]	THPTA [mM]	NaAsc [mM]	Additive	recovery [%]	Products
**1**[1]	0.1 M PBS,7	0.4	-	-		7	not detectable
**2**[1]	0.1 M PBS,7	0.4	2	-		7	not detectable
**3**[1]	0.1 M PBS,7	0.4	2	0.7		10	not detectable
**4**[1]	0.1 M PBS,7	0.4	2	0.7		15	not detectable
**5**[1]	0.1 M PBS,7	0.05	0.25	0.4		30	not detectable
**6**[1]	0.1 M PBS,7	0.1	0.5	0.4		15	not detectable
**7**[1]	0.1 M PBS,7	0.05	0.25	0.4		98	linker cleavage, monooxygenation
**8**[1]	0.1 M PBS,7	0.05	0.25	4		100	diverse
**9**[1]	0.1 M PBS,7	0.05	0.25	0.4	Aminoguanidine	100	linker cleavage
**10**[1]	0.1 M PBS,7	0.05	0.25	4	Aminoguanidine	76	linker cleavage + ascorbic acid adduct
**11**[1]	0.1 M PBS,7	0.05	0.25	0.4	Aminoguanidine, DMSO	100	linker cleavage + ascorbic acid adduct
**12**[1]	0.1 M PBS,7	0.05	0.25	4	Aminoguanidine, DMSO	100	linker cleavage + ascorbic acid adduct
**13**[1]	0.1 M PBS,7	0.1	0.5	0.4	Aminoguanidine	100	linker cleavage
**14**[1]	0.1 M MES, 5.5	0.1	0.5	0.4	Aminoguanidine	3	unknown product (58772)
**15**[1]	0.1 M MES, 6	0.1	0.5	0.4	Aminoguanidine	10	unknown product (58772)
**16**[1]	0.25 TRIS, 8	0.1	0.5	0.4	Aminoguanidine	90	linker cleavage
**17**[1]	0.1 M PBS, 7	0.1	0.5	-	5 mM NH_2OH	39	linker cleavage + adduct (+177)
**18**[2]	1mM PBS, 7.4	5	10	-		22	adducts (+32 and + 376)
**19**[2]	1 mM PBS, 8	5	10	-		13	not detectable

**20**[2]	0.25 M TRIS, 8	5	10	-	12	not detectable

1 Cu(II)SO_4 was used as Cu source

2 Cu(I)Br was used as Cu source

4.2. Conjugation of CRM_{197} with monofunctionalized linker 21

4.2.1. Installation of the monofunctionalized linker 21 on CRM_{197}

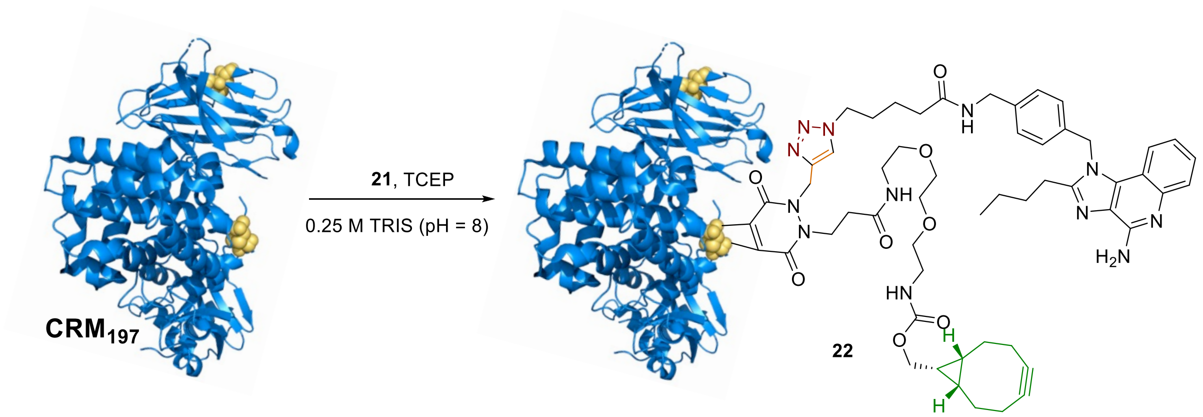

Table S4 Condition screening towards conjugate **22**

Entry	Buffer (pH)	additive	T [°C]	time [h]	CRM_{197}/**24**	recovery [%]
1	TRIS (8)	DMSO (4 %)	0	16	1/0.16	94
2	TRIS (8)	DMSO (4 %)	21	16	1/0.47	70
3	TRIS (8)	DMSO (10 %)	21	16	1/0.45	77
4	TRIS (8)	DMSO (4 %)	37	16	double substitution	48
5	PBS (8)	DMSO (4 %)	37	16	double substitution	19
6	PBS (7.4)	DMSO (4 %)	37	16	double substitution	10
7	TRIS (8)	DMSO (4 %)	21	40	0.14/1	100
8	TRIS (8)	DMSO (4 %)	21	88	0.11/1	72
9[a]	TRIS (8)	DMSO (4 %)	21	64	1/0.76	46
10[b]	TRIS (8)	DMSO (4 %)	21	64	1/0.43	84

a 10 eq. Reagent + 5 eq. TCEP

b 2.5 eq. Reagent + 1.5 eq. TCEP

Optimized reaction conditions: CRM_{197} (0.5 mg; 8.6 nmol) was dissolved in TRIS-buffer (0.4 mL; 250 mM, pH = 8), followed by the addition of linker **22** (20 µL; 10 mg/mL in DMSO; 171.2 nmol). After stirring for 5 min, a solution of TCEP × HCl (12.2 µL, 2 mg/mL in water) was added and stirring was continued for 40 h more at 22 °C. After that the sample was spin filtered against water (cut-off 10 kDa, 3 times) which afforded 200 µL aqu. solution. Nanodrop analysis (E 1% = 10.7) revealed a concentration of 1.8 mg/mL which refers to a total protein recovery of 0.36 mg (72 %) and analysis by ESI-QTOF-MS revealed a substrate conversion of ca. 90 %.

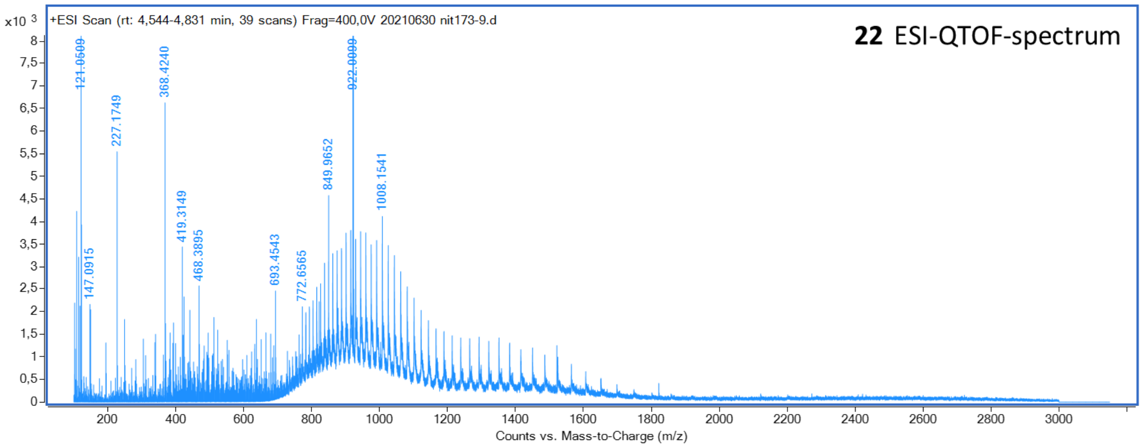

SI Figure 14 ESI-QTOF spectra of **22**

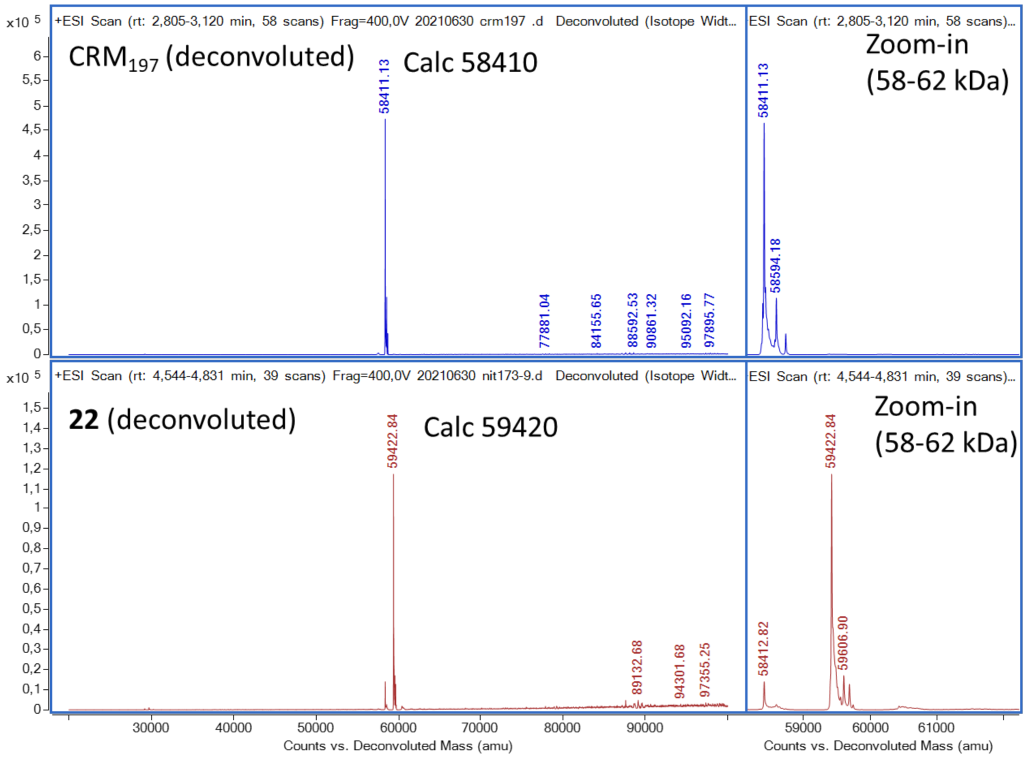

SI Figure 15 Deconvoluted ESI-QTOF spectra of CRM_{197} and **22**

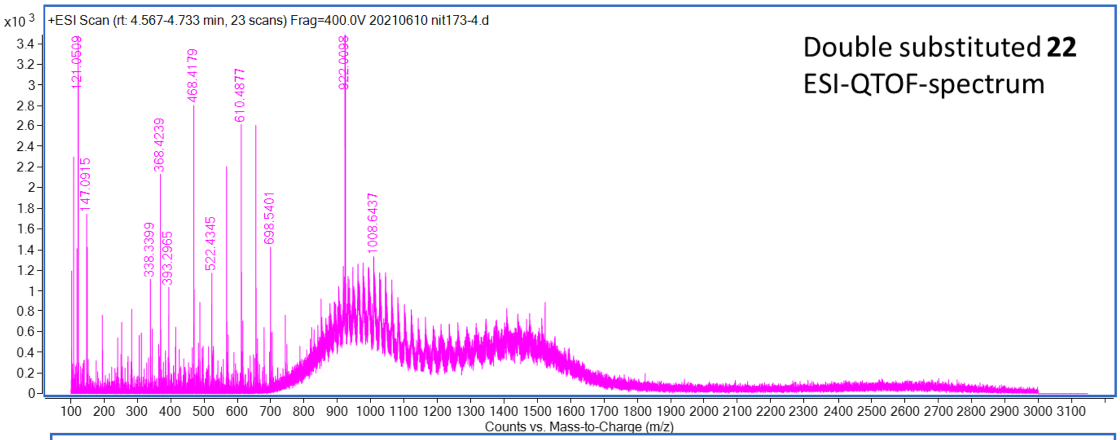

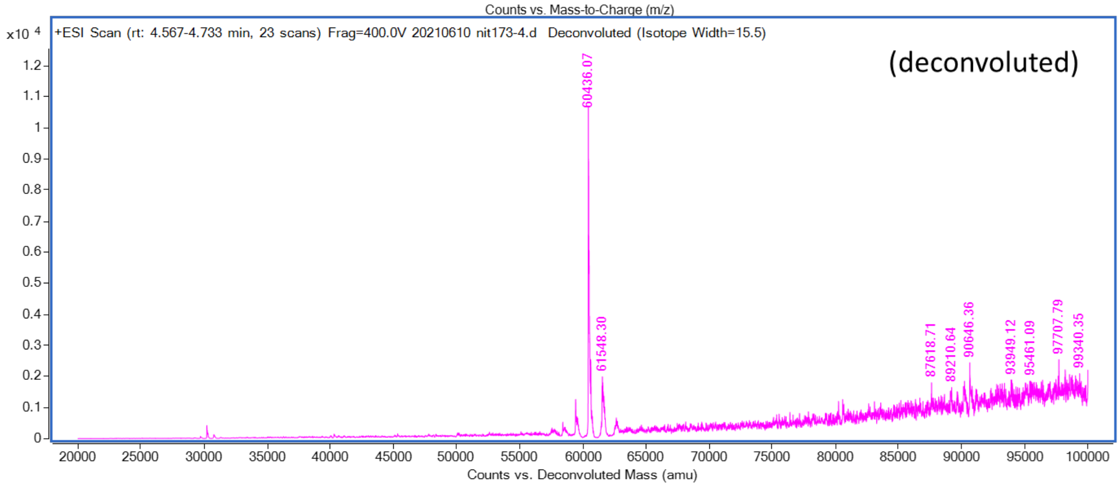

SI Figure 16 ESI-QTOF spectra and deconvoluted ESI-QTOF spectra of double substituted **22**

4.2.2. Synthesis of conjugate 23

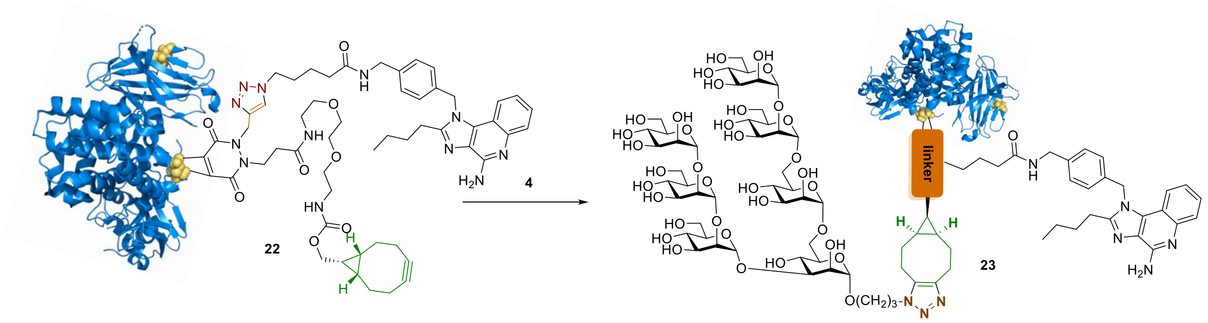

To a solution of linker conjugated CRM **22** (27 µL; 1.8 mg/mL in water; 0.85 nM) was added 27 µL PBS-buffer (pH = 7.4). Subsequently, a solution of **4** (2 µL; 10 mg/mL in water; 16.9 nM) was added and it was stirred at 37 °C for 16 h. After that, the sample was spin filtered against water (cut-off 10 kDa, 3 times) which afforded 100 µL aqu. solution. Nanodrop analysis (E 1% = 10.7) revealed a concentration of 0.37 mg/mL which refers to a total protein recovery of 37 µg (74 %) and analysis by ESI-QTOF-MS indicated product purity of > 80 %

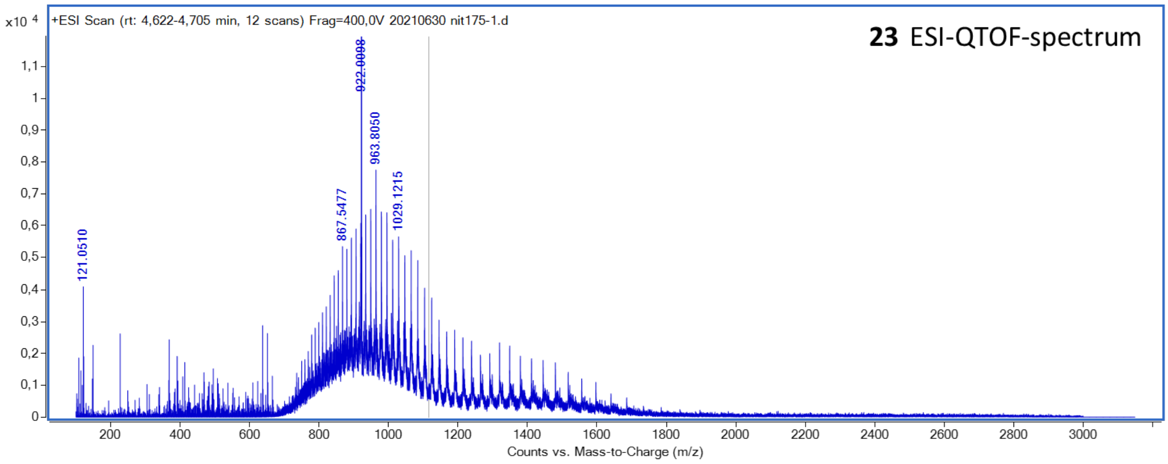

SI Figure 17 ESI-QTOF spectra of **23**

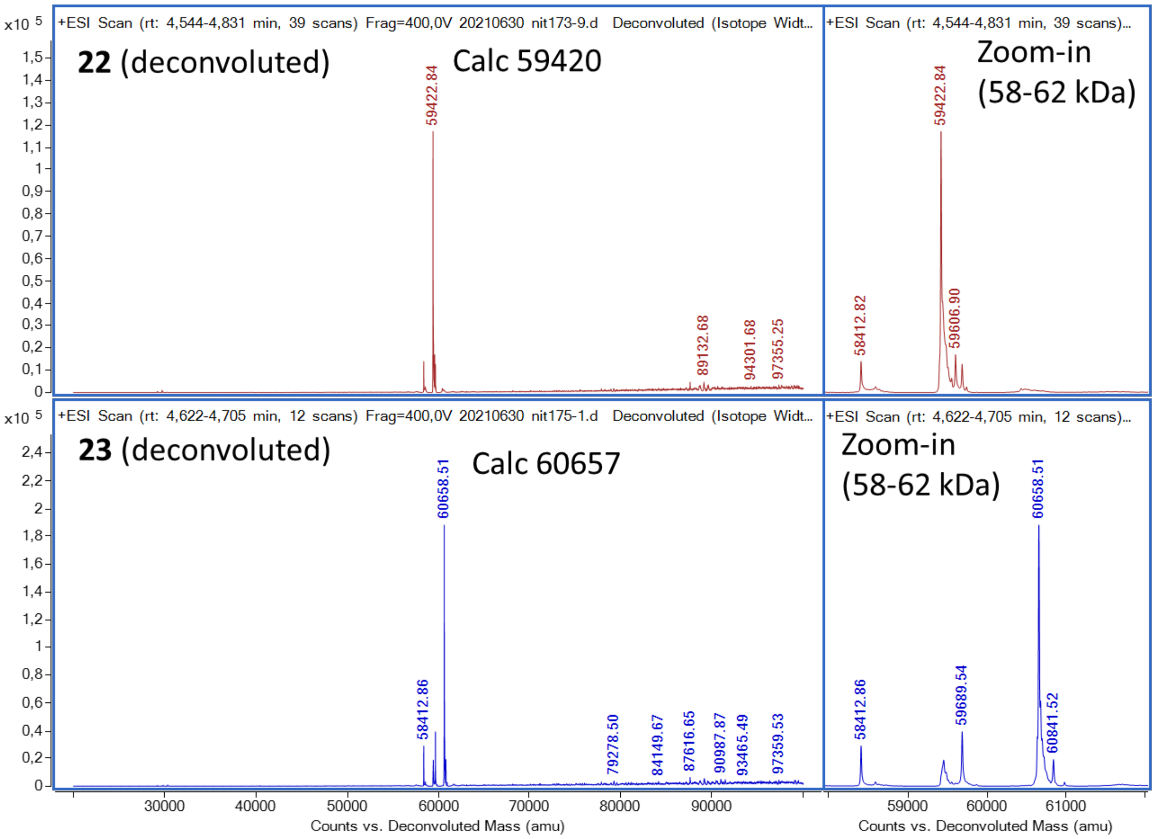

SI Figure 18 Deconvoluted ESI-QTOF spectra of **22** and **23**

4.2.3. Synthesis of conjugate 24

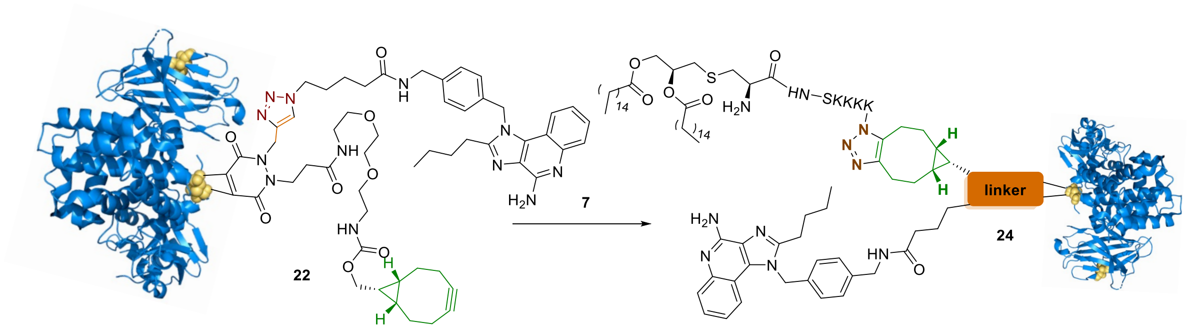

To a solution of linker conjugated CRM **22** (27 µL; 1.8 mg/mL in water; 0.85 nM) was added 30 µL 0.1 M MES-buffer (pH = 6). Subsequently, a solution of **7** (0.6 µL; 10 mg/mL in DMSO; 4.2 nM) was added and it was stirred at 22 °C for 16 h. After that, the sample was spin filtered against water (cut-off 10 kDa, 3 times) which afforded 100 µL aqu. solution. Nanodrop analysis (E 1% = 10.7) revealed a concentration of 0.29 mg/mL which refers to a total protein recovery of 29 µg (58 %) and analysis by ESI-QTOF-MS indicated product purity of > 80 %.

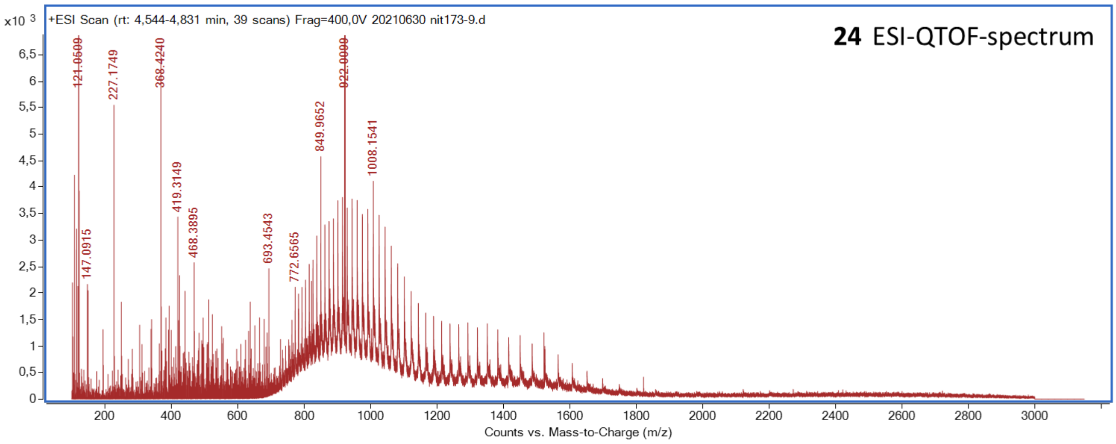

SI Figure 19 ESI-QTOF spectra of **24**

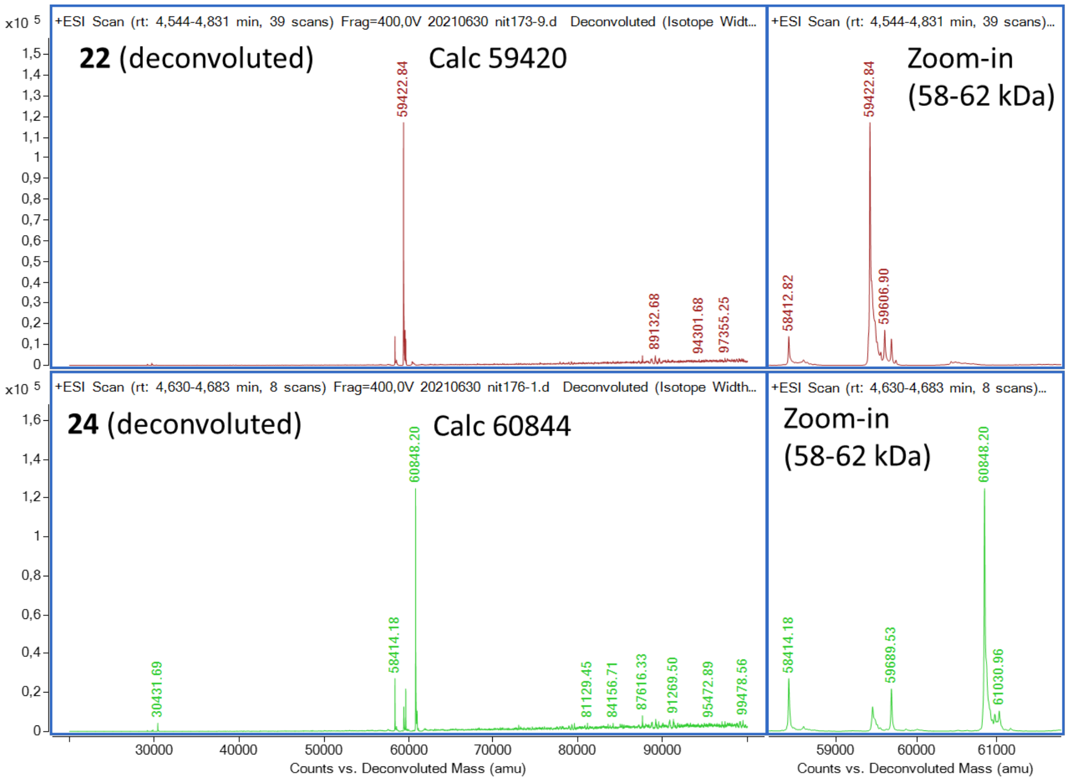

SI Figure 20 Deconvoluted ESI-QTOF spectra of **22** and **24**

4.3. Conjugation of CRM$_{197}$ with difunctionalized linker 13

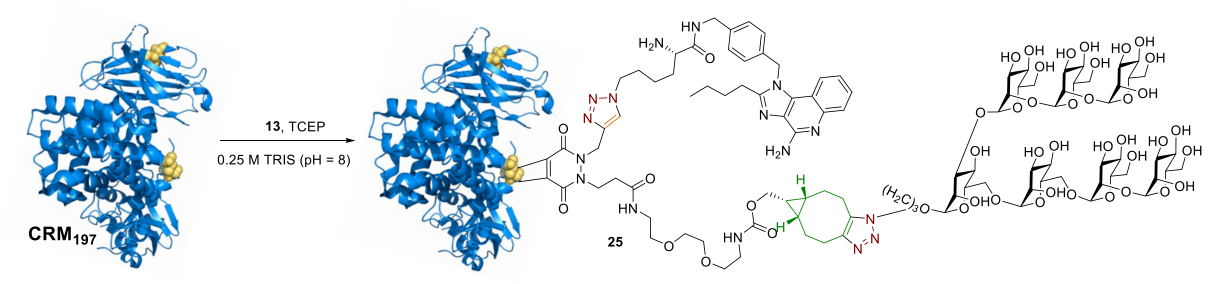

In two parallel reactions, CRM$_{197}$ (50 µg; 0.86 nmol) was dissolved in TRIS-buffer (50 µL; 250 mM, pH = 8), followed by the addition of linker **13** (4 µL; 10 mg/mL in DMSO; 17.1 nmol) and after stirring for 5 min, a solution of TCEP × HCl (1.2 µL, 2 mg/mL in water) was added and stirring was continued for 64 h at 22 (Entry 1) or 37 °C (Entry 2). After that, the sample was spin filtered against water (cut-off 10 kDa, 3 times) which afforded 50 µL aqu. solution. Nanodrop analysis (E 1% = 10.7) revealed a concentration of 0.97 mg/mL (Entry 1) and 0.5 mg/mL (Entry 2) which refers to a total protein recovery of 49 µg (98 %) and 25 µg (50 %) respectively. The samples were further analyzed by ESI-QTOF-MS which revealed a distribution of unconjugated/monoconjugated/diconjugated protein of 1/0.28/0 (Entry 1) and 0.17/1/0.65 (Entry 2).

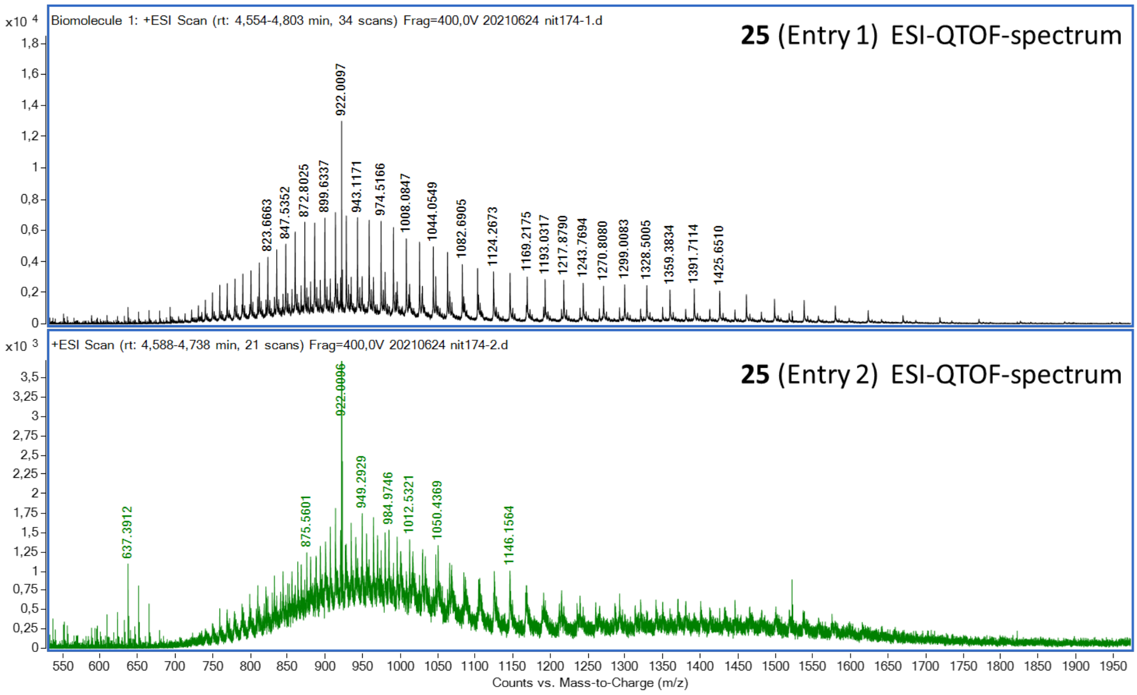

SI Figure 21 ESI-QTOF spectra of 25

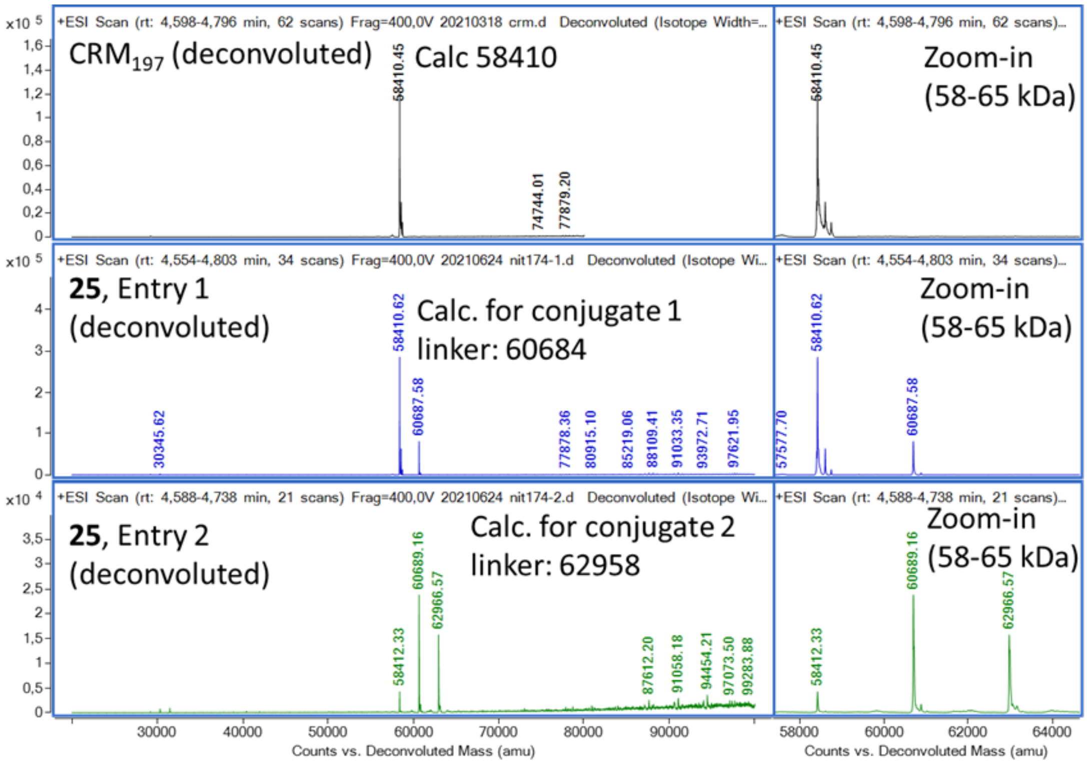

SI Figure 22 Deconvoluted ESI-QTOF spectra of CRM_{197} and 25

5. Random conjugation

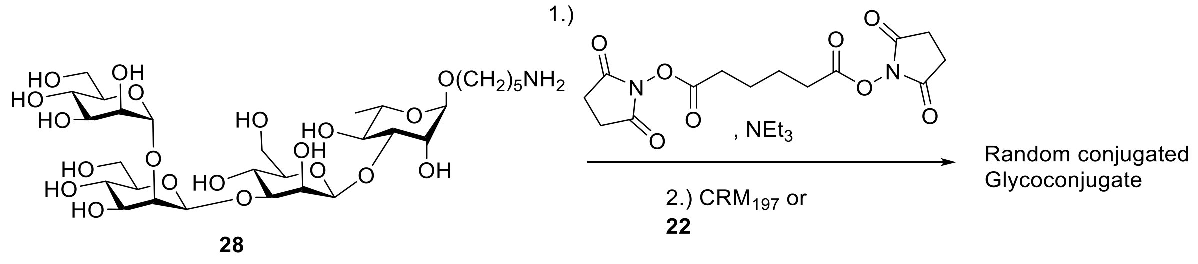

Glycan **28** (0.38 mg; 0.51 µmol) and Bis NHS ester of adipinic acid[5] (1.7 mg; 5.1 µmol) were dissolved in DMSO (0.3 mL) followed by the addition of NEt_3 (0.29 µL; 2.0 µmol) and the mixture was stirred for 4 h at 22 °C. Then, EtOAc (0.6 mL) and PBS buffer (0.3 mL; pH = 7.4) were added and after vigorous shaking the organic phase was removed with a pipette. The aqueous phase was washed two more times with EtOAc and residues of the organic solvent were then removed by bubbling air through the solution. The remaining solution was divided in three portions of which each was either added to a solution of CRM_{197} (0.2 mg in 0.1 mL water; 3.4 nM) or CRM-derivative **22** (0.2 mg in 0.1 mL water; 3.4 nM). The conjugation reaction were gently shook at 22 °C for 16 h and were then purified with spin filtration against water (cut-off 10 kDa, 3 times). Analysis of the conjugates with MALDI-TOF revealed a distribution of protein glycan/carrier: 7.4 (CRM_{197} based conjugate) and 6.9 (**22** based conjugate).

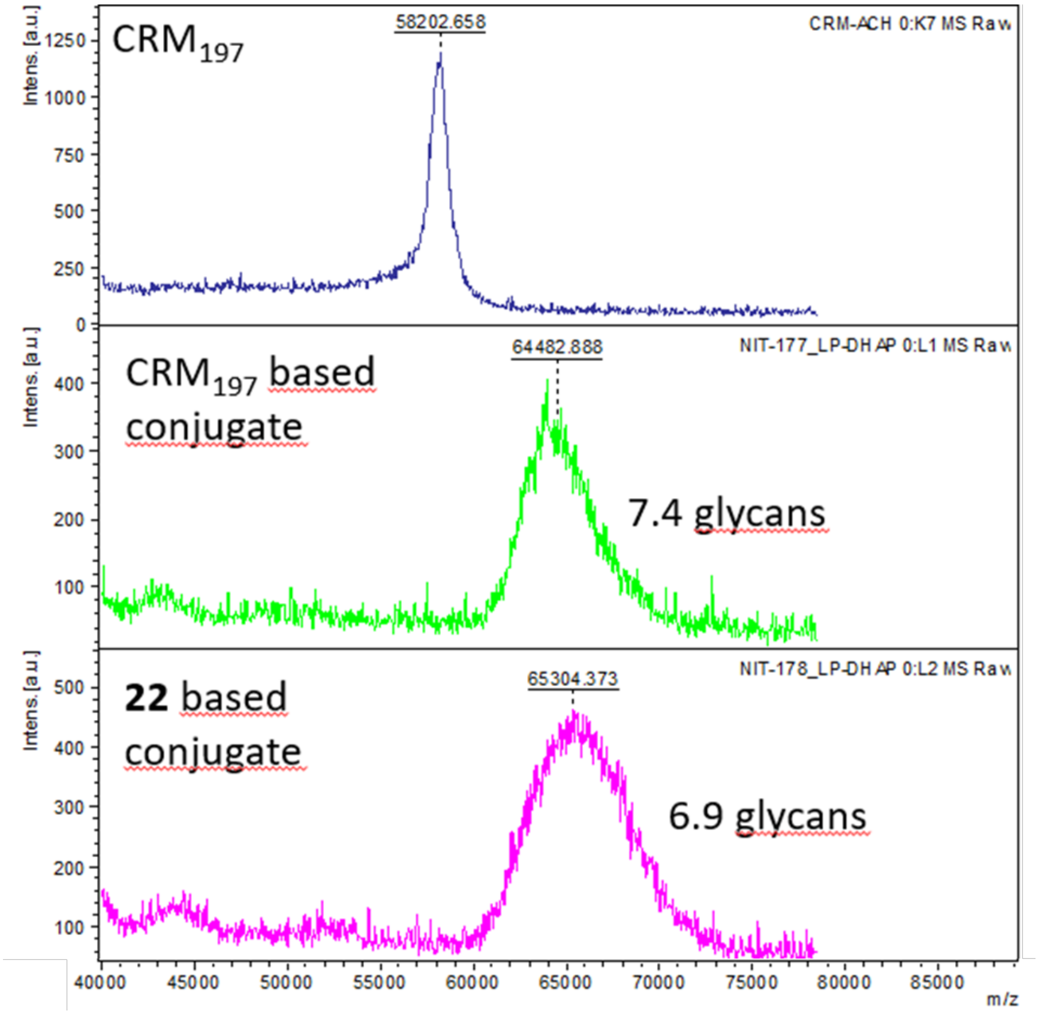

SI Figure 23 MALDI-TOF MS spectra of CRM_{197} and conjugation products

6. Stability studies with conjugate 22

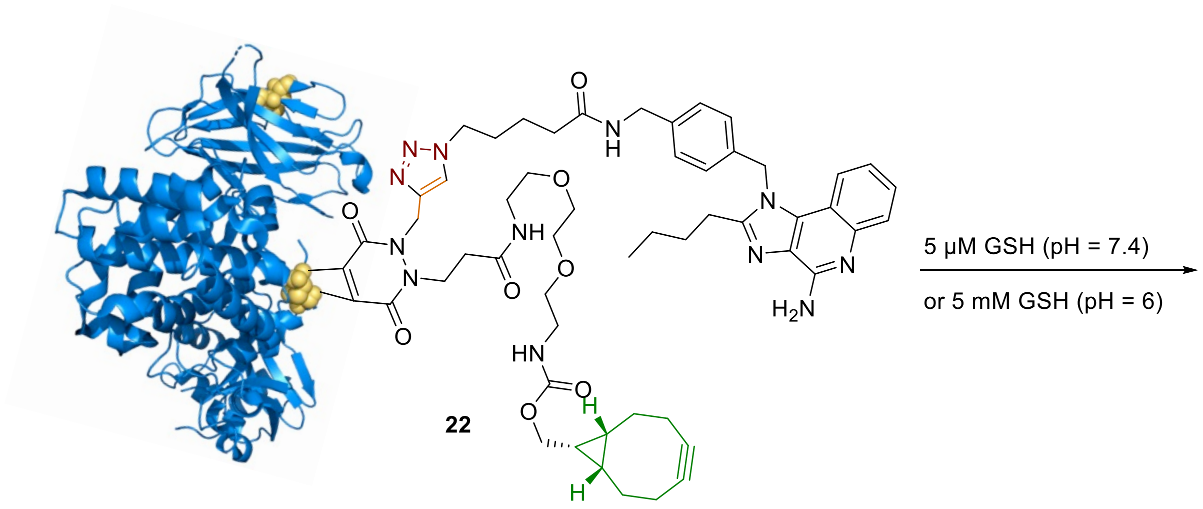

Conditions A: To conjugate **22** (70 µg in 60 µL water) was added GSH in PBS buffer (340 µL, 5 µL GSH, pH = 7.4) and the reaction mixture was incubated for 5 h at 37 °C. Then, 200 µL of the reaction mixture

were removed, spin filtered three times against water (cut-off 10 kDa) and analyzed by ESI-TOF MS. The remaining reaction mixture was incubated for 19 h more, before it was treated and analyzed in as described above.

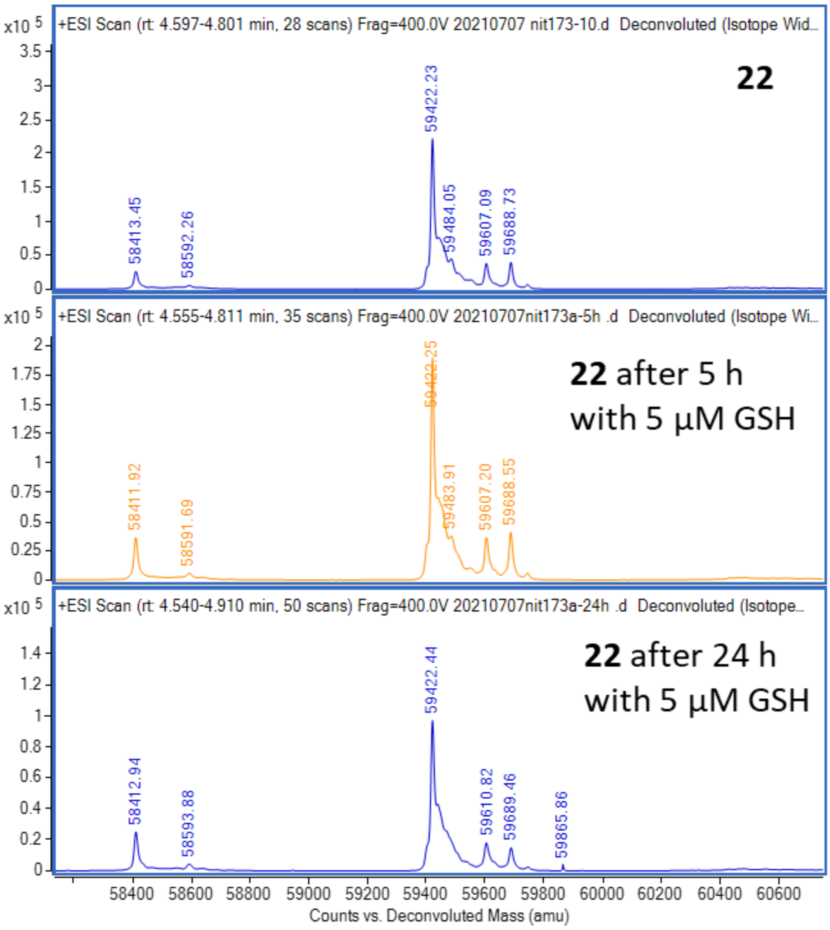

SI Figure 24 ESI-QTOF MS of pure conjugate **22** and treated with 5 µM GSH after 5 and 24 h

Conditions B: To conjugate **22** (70 µg in 60 µL water) was added GSH in PBS buffer (340 µL, 5 µL GSH, pH = 6.5) and the reaction mixture was incubated for 5 h at 37 °C. Then, 200 µL of the reaction mixture were removed, spin filtered three times against water (cut-off 10 kDa) and analyzed by ESI-QTOF MS. The remaining reaction mixture was incubated for 19 h more but was not analyzed further, since full linker cleavage was already detected after 5 h. ESI-QTOF MS of conjugate **22** treated with 5 µM GSH after 5 and 24 h

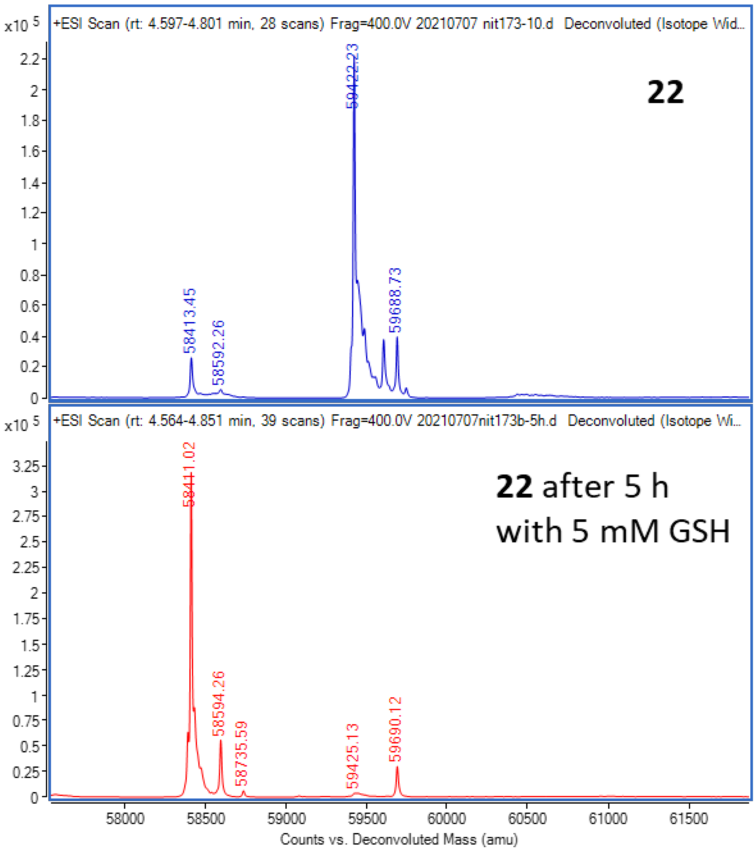

SI Figure 25 ESI-QTOF MS of pure conjugate **22** and treated with 5 mM GSH after 5 h

7. References

[1] E. D. Goddard-Borger, R. V Stick, *Org. Lett.* **2007**, *9*, 3797–3800.

[2] N. M. Shukla, C. A. Mutz, R. Ukani, H. J. Warshakoon, D. S. Moore, S. A. David, *Bioorganic Med. Chem. Lett.* **2010**, *20*, 6384–6386.

[3] A. Maruani, P. A. Szijj, C. Bahou, J. C. F. Nogueira, S. Caddick, J. R. Baker, V. Chudasama, *Bioconjug. Chem.* **2020**, *31*, 520–529.

[4] N. Trattnig, P. Mayrhofer, R. Kunert, L. Mach, R. Pantophlet, P. Kosma, *Bioconjug. Chem.* **2019**, *30*, 70–82.

[5] N. K. Mishra, R. N. V Krishna Deepak, R. Sankararamakrishnan, S. Verma, *J. Phys. Chem. B* **2015**, *119*, 15395–15406.
